# A century of anthropogenic perturbations impact genomic signatures of the iconic migratory Atlantic cod

**DOI:** 10.1126/sciadv.adp3342

**Published:** 2025-07-30

**Authors:** Cecilia Helmerson, Joël M. Durant, Siv Nam Khang Hoff, Marine Servane Ono Brieuc, Paul Ragnar Berg, Per Erik Jorde, Marius Filomeno Maurstad, Ian Bradbury, Olav Sigurd Kjesbu, Jane Aanestad Godiksen, Nils Chr. Stenseth, Bastiaan Star, Kjetill S. Jakobsen, Sissel Jentoft

**Affiliations:** ^1^ Centre for Ecological and Evolutionary Synthesis, University of Oslo, Oslo, Norway.; ^2^ Institute of Marine Research, Bergen, Norway.; ^3^ Norwegian Institute for Water Research, Oslo, Norway.; ^4^ Institute of Marine Research, Flødevigen, Norway.; ^5^ Fisheries and Oceans Canada, Newfoundland, St John’s, Canada.

## Abstract

Anthropogenic stressors have led to marked ecosystem perturbations, including population declines and shifts in habitat range for key marine fish species. Understanding how these changes affect genome-wide characteristics, causing long-term evolutionary responses, is still in its infancy. Genome-wide retrospective assessment of the iconic migratory Atlantic cod (*Gadus morhua*) from the Barents Sea, unraveled varying degree of admixture with the nonmigratory coastal ecotype throughout the 20th century, and intriguingly more intensified during recent decades. These genomic changes were supported by an increased number of individuals displaying the heterozygous state of the chromosomal inversion coupled to migratory behavior in Atlantic cod. Ecological models and genome-wide scans identified that some of the observed frequency shifts, coupled to neural development, metabolic, and growth regulation, covaried with intensified fisheries, reduced generation time, and ocean warming. Our results demonstrate how anthropogenic perturbations impact the dynamics between two well-known ecotypes of Atlantic cod and thus, their genomic signatures, with potential implications for future management programs.

## Teaser

Retrospective assessment of the iconic migratory Atlantic cod uncovers human-induced genome-wide changes during the last century.

## INTRODUCTION

Ocean warming and other anthropogenic stressors, including intensified harvesting, have been identified as environmental perturbations that are likely to affect species distributions, phenology, and demography with knock-on consequences on ecosystem functioning and services (*1*). Changes in habitat use, e.g., northward expansions during warmer years, reduced spawning stock biomass (SSB), and reduced size and age at sexual maturation have been documented for several economically important fish species, and linked to climatic changes and/or intensified fisheries (*2*–*5*). How and if such ecosystem alterations affect the genome-wide characteristics of a species, i.e., causing long-term evolutionary responses, have been heavily debated over the past decades and with the sparse data generated so far, this question is yet to be fully examined (*6*–*9*).

**Fig. 1.**
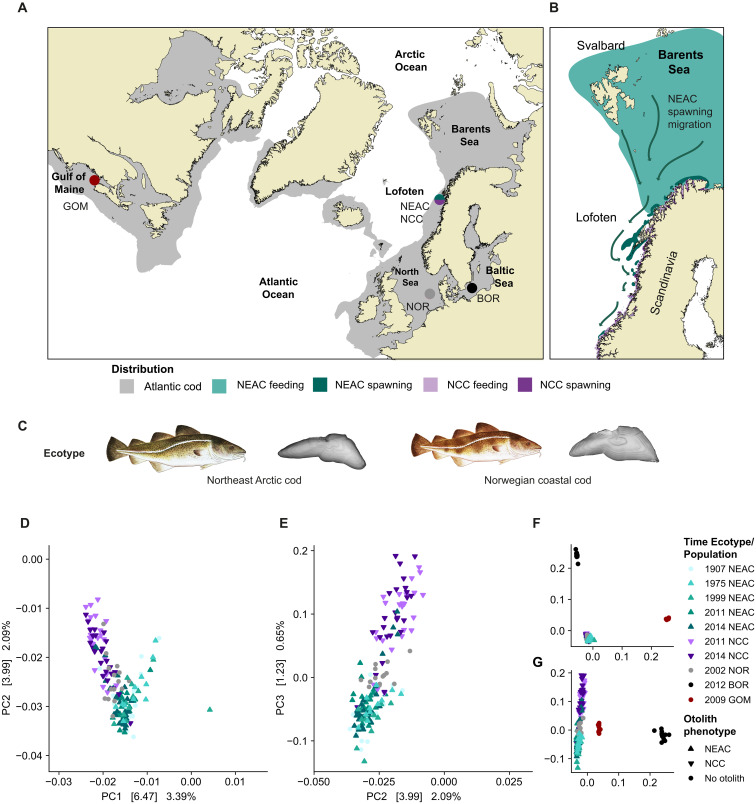
Sampling locations, ecotype/phenotype definitions and genome-wide differentiation of NEAC and NCC. (**A**) Map with geographical distribution of Atlantic cod marked in gray, and denotation of location and ecotype/population included in the study: Lofoten [Northeast Arctic cod (NEAC; blue-green) and Norwegian coastal cod (NCC; purple)], North Sea (NOR; gray), Bornholm Basin (BOR; black), and Gulf of Maine (GOM; brown-red). (**B**) Zoom-in of the Barents Sea and the Scandinavian peninsula, visualizing the feeding ground versus spawning ground for NEAC and NCC. The maps are made in ggOceanMaps (*118*). Distribution data obtained from FAO (*119*) and IMR (*120*). (**C**) Drawings of NEAC and NCC accompanied with cut throughs of respective otoliths, used for phenotypic discrimination between ecotypes (*101*). (**D**) Zoom-in of PCA plot (PC1 versus PC2) of the genome-wide differentiation for the reduced dataset (excluding the inversions and pruned for linkage). (**E**) Zoom-in of PCA plot (PC2 versus PC3) of the genome-wide differentiation for the reduced dataset. (**F**) Full plot PC1 versus PC2. (**G**) Full plot PC2 versus PC3. Upward triangle denotes samples defined as NEAC (based on the otolith reading) while triangle down denotes samples defined as NCC (based on the otolith reading). Circle denotes samples where otolith readings are missing. Fish illustrations by C.H. (UiO) and otolith photo taken by C. Denechaud (IMR).

One of the most iconic fish species in the world, the Atlantic cod (*Gadus morhua*), which is widely distributed in the North Atlantic Ocean (Fig. 1A) and constitutes several of the most economically important fish stocks in the world (*10*), has throughout the last century undergone larger fluctuations (*11*–*13*). From the late 1980s until mid-1990s, the migratory Northeast Arctic cod (NEAC) faced marked reduction in abundance accompanied with decline in size and age at maturation (*14*). These demographic shifts observed were speculated to be linked to intensified harvesting and/or coupled to environmental changes, such as increased sea surface temperature (*15*). In particular, the technological advancements made after the Second World War, including the developments within acoustic applications and navigation systems (*16*, *17*), accelerated the fishing efficiency tremendously and commercial fisheries moved away from a general focus on inshore spawning grounds to offshore feeding grounds (*18*, *19*). Such modifications in fishing selectivity, by increasing the catch of immature cod, and thus, removing a larger proportion of younger year classes, are suggested to have a severe negative impact on relative stock abundance (*20*, *21*).

The first attempts at analyzing whole-genome sequence data of Atlantic cod, collected before and after periods of intensive exploitation (early 20th century versus early 21st century) from both sides of the North Atlantic Ocean, found no larger genome-wide signatures of selection (*6*). In a follow-up study, however, signals of parallel polygenic adaptation to anthropogenic pressure were demonstrated (*22*). Expanding from the above-mentioned studies, we here take a closer look at the genome-wide characteristics of the migratory NEAC ecotype from multiple time periods successively throughout the last century. We also include the nonmigratory Norwegian coastal cod (NCC) in our study design, which is found to have overlapping spawning grounds with NEAC in the northern part of Norway (*23*). In contrast to the more cold-water adapted NEAC, well-known for its yearly spawning migration moving from the feeding grounds in the Barents Sea to the coast of Norway, the nonmigratory NCC ecotype stays near the coast all year around (*24*) (Fig. 1, B and C). Recent reports have documented that the spawning route for NEAC is affected by climatic variability (*25*, *26*), and skipped spawning by females is frequently observed (*27*). Moreover, the distribution of cod at the spawning grounds seems also to be dependent on local environmental conditions, such as vertical temperature stratification (*28*). How these behavioral alterations affect the dynamics and thus the degree of interbreeding events between the two ecotypes have not yet been investigated. Genome-wide analyses have so far shown that the two cod ecotypes are by large discriminated by four chromosomal inversions, while the rest of the genome displays subtle or low genetic differentiation (*29*). This and other studies highlight the impact of chromosomal inversions (hereafter inversions) in evolutionary processes, acting as “supergenes” and thus facilitate local adaptation, despite high connectivity and gene flow, due to lower recombination within these larger haplotype blocks (*29*–*31*). For Atlantic cod, there is evidence that the four larger inversions play a major role in local adaptation to environmental conditions linked to oxygen and salinity (*32*) as well as migratory behavior (*29*, *30*, *33*). The role of potential candidate genes coupled to functional shifts and thus involved in local adaptation should, however, not be neglected. For instance, the previously detected hemoglobin polymorphisms by Sick (*34*) were shown to discriminate between southern cod populations (*34*), and later associated with temperature adaptations across the species distribution range (*35*, *36*).

In this undertaking, the genome-wide retrospective screening of the migratory NEAC, spanning both colder phases (i.e., beginning of the century and in the 1960–70s) and warmer phases [in the 1940–50s and from the 1990s until nowadays (*37*)], uncovered cyclic trends of directional gene flow from nonmigratory coastal cod into the migratory ecotype during the last century. Especially, from the late 1990s and onward, covering periods of intensified fisheries and an overlaying trend of ocean warming (*11*), we detected an increased frequency of coastal cod ancestry at the whole genome–wide level accompanied with higher frequency of heterozygous individuals (HET) for the major inversions associated with migratory behavior in Atlantic cod (*29*, *38*, *39*). Our findings combined indicate that the two ecotypes are interbreeding more frequently in modern times than historically or that the HET individuals experience a higher survival rate than the homozygous individuals, e.g., displaying more favorable behavior in terms of avoidance of being fished upon and/or higher degree of phenotypic plasticity in response to warmer waters. Together, we demonstrate that genome-wide shifts have occurred in the NEAC population during the past century, where the dynamics between the two cod ecotypes are seemingly playing an important role in their response to the ongoing anthropogenic perturbations.

## RESULTS

### Temporal population genomic differentiation and signals of admixture

Temporal population genomic differentiation of NEAC was investigated using the “neutral” genome-wide datasets (i.e., pruned for linkage and excluding the inversions), from both the reduced (excluding year 1940 and 1958) and full dataset (including all years: 1907, 1940, 1958, 1975, 1999, 2011, and 2014); see Materials and Methods for more information. For the three significant principal components (PC1 versus PC2 and PC2 versus PC3), the historical NEAC individuals clustered (by large) together with their modern counterparts (year 2011 and 2014; Fig. 1, D and E, and fig. S1, A and B). The Canadian samples [Gulf of Maine (GOM)] and the Baltic samples [Bornholm Basin (BOR)] were uniquely assigned in two separate clusters (Fig. 1F and fig. S1C). However, a slight differentiation was detected between some of the historical years versus the modern samples. Within the reduced dataset, individuals from year 1975 were found to be significantly different from those from 2014, while in the full dataset, individuals from years 1958 and 1975 were found to be significantly different from those captured in year 2014 (for more details, see text S1 and tables S1 to S6). Moreover, along the third principal component (PC3) for both datasets separation between NCC, the North Sea (NOR) samples and NEAC was observed (Fig. 1, E and G, and fig. S1, B and D). The majority of the Eigbest single-nucleotide polymorphisms (SNPs) defined by the Smartpca (*40*, *41*) analysis as contributing the most to principal components analysis (PCA) differentiation were shared (70%) between the two datasets (table S3 and table S6).

**Fig. 2.**
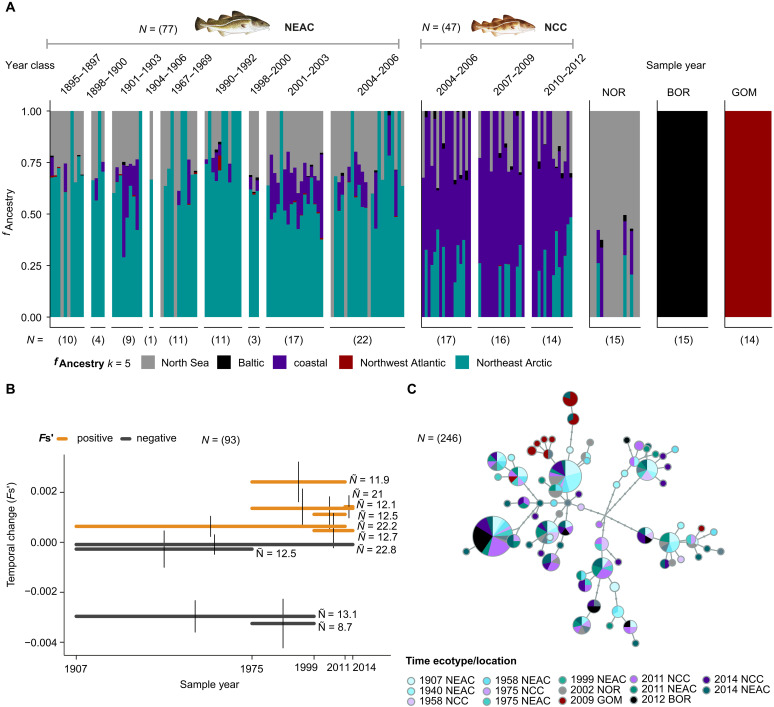
Signature of admixture, *F*_s_’ temporal frequency shifts, and mt haplotype network in NEAC over the past century. (**A**) Admixture analysis (*k* = 5) with denotation of the proportion of NOR (gray), Baltic (black), Coastal (purple), Northwest (brown-red), and Northeast Arctic (blue-green) ancestry for NEAC, NCC, NOR, BOR, and GOM. The time series for NEAC is displayed on the basis of year of birth (divided into 3-year classes). Number of SNPs used *n* = 141,549. Sample sizes shown within brackets. (**B**) *F*_s_’ temporal allele frequency shifts within sample intervals (pairs of sample years) with 95% confidence interval (vertical bars) and harmonic mean sample size (*Ñ*). Positive *F*_s_’ are colored orange, negative colored dark gray. Number of SNPs used *n* = 141,549. (**C**) mt haplotype network for the protein coding genes, with NEAC (blue-green), NCC (purple), NOR (gray), BOR (black), GOM (brown-red), and historical samples (shades of light blue-green and purple). Number of SNPs used *n* = 11,418, yielding 58 informative variable sites. Fish illustrations by C.H. (UiO).

The ADMIXTURE (*42*) analyses corroborated the results from the PCA, where the first group separating out was the GOM versus the rest of the samples, i.e., a separation between the West and East Atlantic ancestry (Figs. 1, F and G, and 2A, and figs. S2 and S3). The second group to separate out was BOR (Fig. 2A and figs. S2 and S3), i.e., corresponding to the separation seen on PC2 (Fig. 1, F and G). For the two next levels (*k* = 4 and *k* = 5), we were able to distinguish between NEAC ancestry versus NCC versus NOR ancestry. Intriguingly, for both datasets, we identified significant differences in degree of coastal cod ancestry within the migratory NEAC ecotype throughout the past century (Fig. 2A and fig. S4). For the reduced dataset the highest degree of admixture was detected for NEAC individuals with birth year 2001 to 2003 and 2004 to 2006, with a coastal cod ancestry estimated to 17 and 7% of the genome, respectively (Fig. 2A and table S8). In addition, we identified a higher degree of coastal cod ancestry (15%) for individuals with birth year 1901 to 1903, while lower levels before and after: spanning from 2% in 1895 to 1897 and 4% in 1898 to 1900; 2 to 3% in 1967 to 1969 and 1990 to 1992 and 4% in 1998 to 2000. Similar trends but less pronounced were observed in the full dataset (*P* value = 0.0005 and *P* value = 0.02, respectively). See text S2, tables S7 to S10, and figs. S2 to S4 for more information.

When analyzing the reduced dataset, the temporal allele frequency shifts [pairwise comparisons between older and more recent NEAC samples, *F*_s_’ (*43*), a measure of temporal frequency shift] were generally small and, when adjusted for sampling effects, close to zero or even negative (Fig. 2B). This near stability is foremost exemplified by the longest temporal interval, between 1907 and 2014, which had a point estimate of *F*_s_’ slightly below zero (−0.00009, Fig. 2B), implying that the observed allele frequency differences (AFDs) were no larger than expected solely due to sampling “noise” (i.e., within error margins). The corresponding estimate of effective population size (*N*_e_) was thus “infinity” (*44*). Nevertheless, some internal time intervals yielded positive estimates, even with 95% confidence intervals above zero. These intervals included all comparisons with samples from 2011 as well as most of the comparisons with samples from 2014, indicating that the allele frequencies for these years deviated not by genetic drift but by introgression of genes with coastal cod ancestry. Similar results were also found for the full dataset, with positive estimates for comparisons with 2011, 2014, and 1907, i.e., years when an increased coastal cod ancestry was detected (see above and text S3 and fig. S5). Besides, the NEAC stock appears to be of considerable effective size, with no larger shifts over the past century as also shown in a previous study by Pinsky *et al.* (*6*). Moreover, the mitochondrial (mt) network analysis uncovered a mixed clustering pattern for the historical NEAC versus modern NEAC samples and the other samples (NCC, NOR, and BOR; Fig. 2C). For more information on the mt clustering see text S4.

### Temporal inversion and hemoglobin frequency shifts

**Fig. 3.**
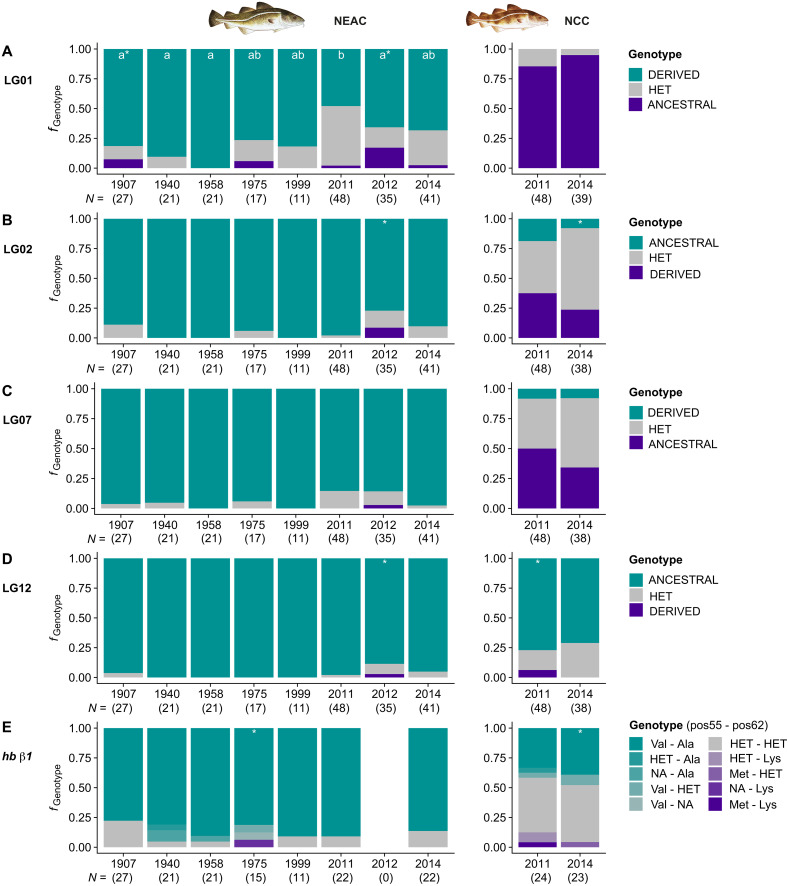
Inversion and hemoglobin genotype frequencies for NEAC and NCC. Inversion genotype frequencies at catch year for NEAC during the time series and NCC for two modern time points on (**A**) LG01, (**B**) LG02, (**C**) LG07, (**D**) LG12, and (**E**) for the hemoglobin locus *hb* β*1* genotype frequency. The inversion genotype plots include data from multiple SNP datasets (see Materials and Methods for more details). Sample sizes shown at bottom within brackets. Genotypes colored as NEAC dominating (blue-green) and NCC dominating (purple). Inversion genotypes given as homozygous DERIVED, heterozygous (HET), and homozygous ANCESTRAL. Hemoglobin genotypes given as the combination over pos55 and pos62, i.e., as homozygous Val-Ala, double HETs, and homozygous Met-Lys. Statistically significant groups of Fisher exact test marked with letters on bars. Groups deviating from HWE with χ^2^ test marked with *. For *hb* β*1*, the HWE deviations detected are for pos62. The NEAC inversion proportions throughout time are dominated by DERIVED genotype for LG01 (A) and LG07 (C) and ANCESTRAL for LG02 (B) and LG12 (D). Fish illustrations by C.H. (UiO).

For calculation of temporal frequency shifts for the well-known inversions (*29*, *31*), the majority of the historical NEAC individuals displayed the homozygous DERIVED state of the inversion on linkage group (LG)01 and LG07 and the homozygous ANCESTRAL state of the inversion of LG02 and LG12, which is consistent with the genotype typically observed for NEAC (*29*, *31*). However, significant shifts in frequencies for the LG01 inversion type over the time period tested were identified, with a pronounced increase in HETs during the past decades (see Fig. 3A, text S5, and table S11). For the inversion on LG02 a close to statistically significant differentiation (*P* = 0.06), with increase in HETs for the same time period as for the inversion on LG01, was observed (see Fig. 3B and table S11). For the other two inversions, LG07 and LG12, no significant differentiation (over the years tested) was detected (see Fig. 3, C and D, and table S11). It should be noted that deviations from Hardy-Weinberg equilibrium (HWE) were found for some of the years (marked with asterisk in Fig. 3, A, B, and D; and marked in red in tables S12 to S15).

For the investigation of the hemoglobin polymorphism previously detected in Atlantic cod, i.e., two nonsynonymous substitutions (*35*, *36*), the majority of the NEAC individuals was homozygous for the Val-Ala genotype. The Val-Ala is denoted as the cold water–adapted genotype, which is found in higher frequencies in the northernmost Atlantic cod populations versus the Met-Lys is denoted as the warm water-adapted genotype, which is found in higher frequencies in the southernmost populations (*35*, *36*). A few double HET (HET-HET) individuals were observed for some of the years, as well as one Lys individual in 1975. The subtle changes in hemoglobin genotype frequencies over the whole time period investigated were not significant (see Fig. 3E and table S16). However, for some of the years, deviations from HWE were detected (marked with asterisk in Fig. 3E, and marked red in tables S17 and S18).

### Inversion frequency shifts and hemoglobin genotypes over time coupled to demographic and environmental variables

**Fig. 4.**
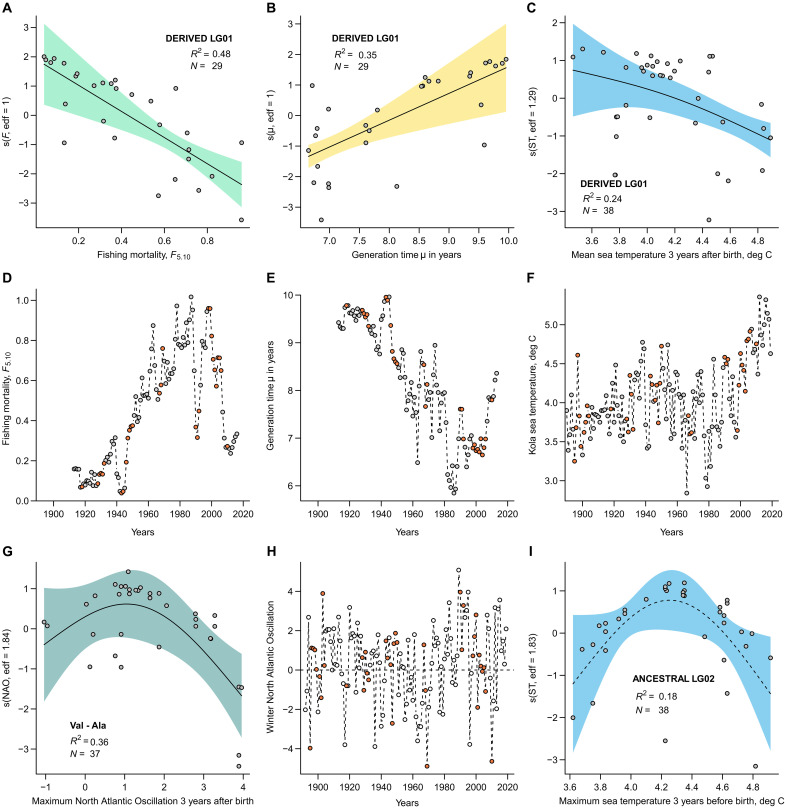
Impact of demographic environmental variables on shifts in inversion genotype and *hb* β*1* frequency during the last century in NEAC. Generalized additive model (GAM) prediction partial plots for different explanatory variables (**A**) Fishing mortality (*F*_5.10_), (**B**) Generation time (μ; estimated in years), (**C**) mean ST during the 3 years after birth affecting the frequency of DERIVED inversion genotype on LG01. (**G**) Maximum winter North Atlantic Oscillation (wNAO) affecting the frequency of Val-Ala during the 3 years after birth and (**I**) the maximum ST during the 3 years before birth affecting frequency of the ANCESTRAL inversion genotype on LG02. The *x* axes partial plots display the covariate and the *y* axes are the partial effect that each covariate has on the response variable. s(*X*, *y*) is the smoothing term, where *X* represents the explanatory variable and *y* is the estimated degrees of freedom (edf) of the smoothing term. Black line shows the smooth term effect of the considered covariate on the proportion of the inversion considered with the pointwise 95% confidence interval including the mean effects uncertainty for the smooth terms (shaded area). The dots indicate the residuals. The demographic and environmental variables used in the GAM (**D**) *F*_5.10_, (**E**) μ, (**F**) mean ST, and (**H**) wNAO. The red markings indicate years where the sampled cod were born and thus genomic information is available.

Using generalized additive models (GAM) (*45*), linking the shifts in inversion frequencies (as well as hemoglobin genotypes) over the years to demographic and other environmental variables (see text S6, fig. S6, and table S19), showed that increase in fishing mortality (*F*_5.10_) led to decreased abundance of the homozygous DERIVED genotype on LG01 (*R*^2^ = 0.48, Fig. 4; and the reverse for ANCESTRAL on LG01 *R*^2^ = 0.22 in fig. S7). Similarly, increase in Kola Sea temperature (ST) (*46*) during the 3 years following birth (period from birth to recruitment) led to decreased abundance of the homozygous DERIVED genotype on LG01 (*R*^2^ = 0.24, Fig. 4). Conversely, an increase in generation time (i.e., proxy of the age structure of the population, μ) was found to be positively correlated with an increased abundance of the homozygous DERIVED genotype on LG01 (*R*^2^ = 0.35, Fig. 4). Note that restraining the model to the years after 1980 (years after the NEAC stock recovery) also resulted in a general increase abundance of homozygous DERIVED genotype on LG01 with an increase of the generation time (see fig. S7). For the inversion on LG02, an increase in fishing mortality led to decreased abundance of the homozygous ANCESTRAL genotype (*R*^2^ = 0.31, fig. S7), while increase in maximum value of the winter North Atlantic Oscillation (wNAO) (*47*) recorded during the 3 years before birth led to an increased proportion of the ANCESTRAL genotype (*R*^2^ = 0.37, fig. S7). For inversion on LG12, increase in fishing mortality led to decreased abundance of the homozygous ANCESTRAL genotype (*R*^2^ = 0.34, fig. S7). For the inversion on LG07, no model was found (see table S19). For the relationships with the proportion of Val-Ala, an increase in the wNAO recorded 3 years after birth corresponded to a decreased proportion of Val-Ala (*R*^2^ = 0.36, Fig. 4), showing a similar trend as found for the ANCESTRAL genotype on LG02 when coupled to increased maximum ST 3 years after birth (albeit not significant Fig. 4).

### Inversion genotypes coupled to demographic variables at the individual level

The analyses linking the individual inversion genotypes to individual demographic variables uncovered a significant coupling of age at maturation and the inversion on LG01, where individuals displaying the ANCESTRAL variant were found to display younger age at maturation compared to individuals harboring the DERIVED variant (fig. S8). Significant results were also detected for the Fulton K (*48*) index (condition factor) when coupled to the inversion genotypes on both LG07 and LG01, where HET and ANCESTRAL individuals showed higher condition factor compared to the DERIVED individuals for these two inversions (fig. S9). For growth rate, no significant coupling to the inversion genotypes was detected (fig. S10). For the other tests conducted, see text S6.

### Detection of signatures of selection

**Fig. 5.**
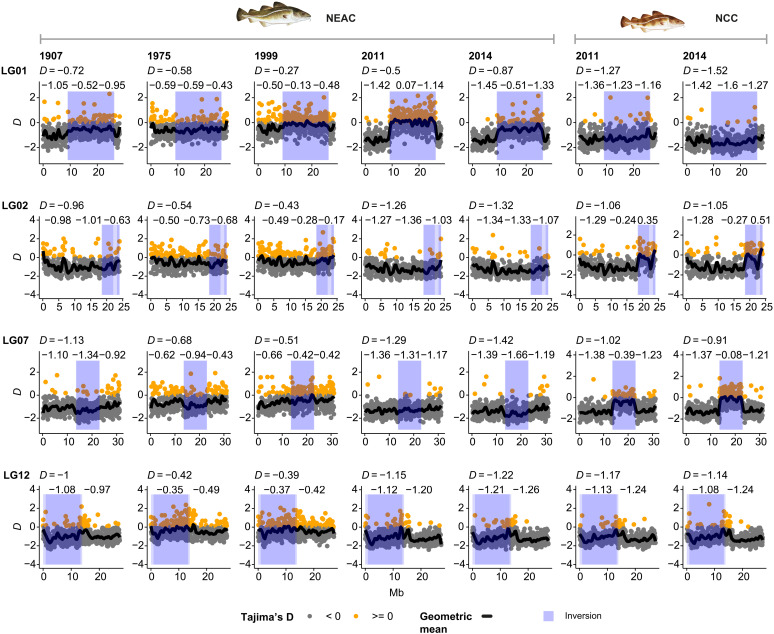
Signatures of selection over the four inversion regions for NEAC over the past century and NCC in modern times. Tajima’s D (using the reduced dataset) along the chromosomes over the inversion regions on LG01, LG02, LG07, and LG12 for NEAC and NCC for the different years. Tajima’s D over or equal to 0 colored in orange and negative colored in gray. Average Tajima’s D for entire chromosome given on top of each plot and averages inside and outside of inversion. Geometric mean shown with black line. Inversion regions marked in blue. Fish illustrations by C.H. (UiO).

The Tajima’s D (*49*) estimates uncovered elevated values inside versus outside of the genomic region covering the inversion on LG01 for NEAC (see Fig. 5) for all years investigated except for 1975. The most pronounced differentiation was observed in 2011. For this particular year, the calculated estimates averaged 0.07 (positive) inside of the inversion, whereas it was found to be negative (between −1.14 and −1.42) outside of the inversion (Fig. 5). For NCC signals of lowered Tajima’s D values were detected for year 2014 (Fig. 5). When calculating the Tajima’s D values for the DERIVED and ANCESTRAL genotypes separately, we identified elevated values for the DERIVED variant in NEAC for 1907, 1975, 2011, and 2014 (fig. S11), while for the ANCESTRAL variant in NCC the signals outside and inside of the inversion, no apparent difference was detected. Signals of selection by the integrated haplotype score (iHS) (*50*) were also detected over the inversion region in NEAC, especially for 1975 and 1999 as well as for NCC (fig. S12).

For the inversion on LG02 no strong signals of selection were detected (Tajima’s D and iHS) in NEAC (see Fig. 5 and fig. S12), while for NCC, positive Tajima’s D values were found for both years included in this study (i.e., 2011 and 2014), indicating the importance of this inversion for the nonmigratory ecotype (Fig. 5). In NEAC, we detected lowered values of Tajima’s D and strong signals of selection (iHS) over the inversion region on LG07, for most of the years investigated (Fig. 5 and fig. S12). For the same inversion on LG07, NCC displayed elevated Tajima’s D values and signals of selection (iHS) for both years included (Fig. 5 and fig. S12). For the inversion on LG12 for both ecotypes over the years investigated, some slight (but not substantial) elevation in Tajima’s D was detected (see Fig. 5 and see fig. S12). Estimation of nucleotide diversity (π) across the chromosomes did not indicate strong signals of selection that could be coupled to the inversion regions for particular years or outlier regions (see https://figshare.com/articles/online_resource/Supplementary_html/28914368?file=54168185).

### Identification of outlier loci and functional annotation

**Fig. 6.**
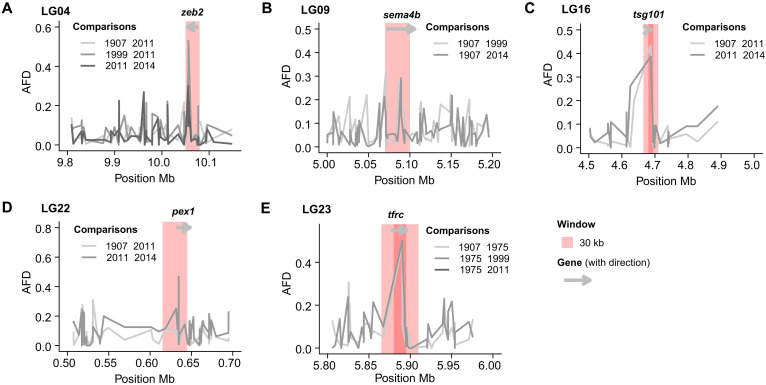
Allele frequency shifts and outlier candidate genes for NEAC over the past century. (A to E) Outliers in more than two pairwise comparisons between years, detected by *F*_ST_, AFD, and/or _XP_EHH analyses, with significant values for *F*_ST_ with a *q* value ≤0.05 and the AFD quantile = 99.9, and the _XP_EHH log(*P*) ≥ 3 are presented with AFD plots. (**A**) On LG04 the outlier identified is positioned inside an intron of the *zeb2* (zinc finger E-box-binding homeobox 2 gene). (**B**) On LG09, the outlier is positioned within the coding region of *sema4b* (semnaphorin-4B) and responsible for an amino acid change. (**C**) On LG16, the outlier is positioned inside an intron of *tsg101* (tumor susceptibility 101 gene). (**D**) On LG22, the outlier is positioned inside an intron of *pex1* (peroxisome biogenesis factor 1). (**E**) On LG23, the outlier is positioned inside an intron of *tfrc* (transferrin receptor 1).

For the inspection of the pairwise fixation index (*F*_ST_) (*51*) outlier regions, there were altogether *n* = 897 outliers (out of *n* = 166,484 sites) with a *q* value less or equal to 0.05 for all the pairwise comparisons between the years using the reduced WGS (whole-genome sequencing) dataset. Out of these outliers, only *n* = 107 were identified in more than two of the pairwise comparisons, and only a minority of these were present in more than three or four pairwise comparisons (*n* = 14 or *n* = 2 respectively, see table S20). Thus, the vast majority of genome-wide shifts detected in the pairwise comparisons were not persistent throughout the time period, while being unique for only a few of the comparisons (for more details see https://figshare.com/articles/online_resource/Supplementary_html/28914368?file=54168185). Further, the outliers detected did not display any signals of selective sweeps; they were rather evenly distributed across the genome (and all chromosomes), i.e., indicative of polygenic allele frequency shifts. Here, we highlight some of the common *F*_ST_ outliers detected between the pairwise comparisons over the years in NEAC, which also showed differentiation in the AFD (*52*) and _XP_EHH (cross-population extended haplotype homozygosity) (*53*) analyses. For LG04, one *F*_ST_ outlier common between 1907 versus 2011, 1999 versus 2011, and 2011 versus 2014, also correspond to AFD outliers for two of the comparisons (see Fig. 6A, fig. S13A, and table S20). This *F*_ST_ outlier was located within the intron region of zinc finger E-box-binding homeobox 2 (*zeb2*) gene. *zeb2* is a DNA binding transcription factor, involved in the transforming growth factor β signaling pathway, and important for early development of neural crest and bony endoskeleton in vertebrates (*54*, *55*). Notably, the neural crest, defined as the “fourth germ layer,” is thought to be a vertebrate innovation and source of a wide range of adult cell types, including varied craniofacial morphologies, pigmentation, and behavior differences (*56*).

One of the *F*_ST_ outliers on LG09 common between 1907 versus 1999 and 1907 versus 2014 corresponded to an AFD outlier in the 1907 versus 2014 comparison (see Fig. 6B, fig. S13B, and table S21). This outlier locus was located within a coding region of the semaphorin-4B (*sema4b*) gene. *sema4b* is found to be involved in immune response regulation (*57*). The SNP identified (TTC → CTC) is located at the very start of exon six and responsible for an amino acid change from Phe (phenylalanine) to Leu (leucin), which could putatively affect the folding/structure and thus, function of the protein. Two of the *F*_ST_ outliers on LG16 were found to be in common between two of the pairwise comparisons (1907 versus 2011 and 2011 versus 2014). Both *F*_ST_ outliers had corresponding AFD outliers that were localized inside an intron of tumor susceptibility 101 (*tsg101*) gene (see Fig. 6C, fig. S13C, and table S21), suggested to function in diverse intracellular processes (*58*). For LG22 one common *F*_ST_ outlier between 1907 versus 1975 and 1975 versus 1999 (see Fig. 6D, fig. S13D, and table S21), corresponded to quantile in AFD and _XP_EHH, both at the same position inside an intron of peroxisome biogenesis factor 1 (*pex1*) gene, an AAA–adenosine triphosphatase (ATPase) involved in peroxisome biogenesis and thus involved in fatty acid metabolism (*59*).

Last, two of the *F*_ST_ outliers on LG23 were found to be common between three of the pairwise comparisons (1907 versus 1975, 1975 versus 1999, and 1975 versus 2011), with matching AFD quantiles (see Fig. 6E, fig. S13E, and table S20). Both of these sites were located within the intron of transferrin receptor 1 (*tfrc*) gene, encoding for transferrin receptor protein 1 (TFRC) that binds transferrin (Tf) and performs a critical role in cellular iron uptake through the interaction with iron-bound Tf (*60*). Thus, the protein is the critical iron supplier to hemoglobin-synthesizing immature red blood cells (*61*), and could potentially be of high importance for Atlantic cod, with its well-known hemoglobin genotypes linked to warm- and cold-water adaptation (*35*). For the other outliers detected, see tables S20 and S21 for more information.

### Detection of adaptive divergence and functional annotation

**Fig. 7.**
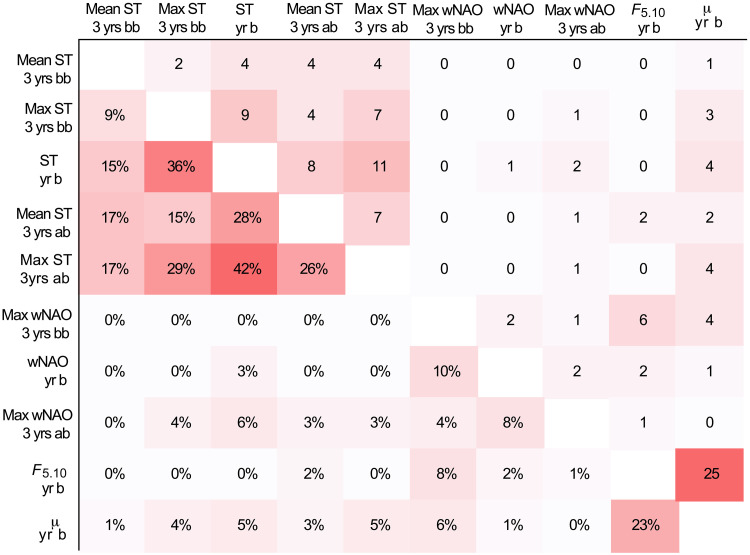
Shared outliers between environmental variables with BayPass. Outliers with XtX above simulation threshold and BF over 20. Right upper half showing number of shared outliers and left bottom half shared percentage. Variables given as ST (Kola Sea temperature), wNAO (winter North Atlantic Oscillation), *F*_5.10_ (fishing mortality), and μ (generation time), 3 years before birth (3 yrs. bb), 3 years after birth (3 yrs. ab), or at year of birth (yr b).

The BayPass (*62*, *63*) genome-wide scans enabled us to further couple the genome-wide allele frequency shifts observed over time to environmental and demographic parameters, and thus, potentially associated to adaptive and/or functional divergence. In these scans, we used the same variables as for the ecological modeling see above and text S6. We uncovered a variable number of outliers for the different tests performed, ranging from *n* = 9 for max wNAO 3 years before birth closely followed by *n* = 10 for mean ST 3 years before birth, to *n* = 62 for generation time (μ) and *n* = 72 for intensified fisheries (*F*_5.10_). In sum, the different analyses yielded 162 outliers of which 47 outliers were shared between two or more of the different variables tested (for more details see https://figshare.com/articles/dataset/Supplementary_BayPass_hits/28151783?file=52219715). The highest number of shared outliers (*n* = 25, yielding 22% of the number of SNPs detected in these tests) were found between *F*_5.10_ and generation time (see Fig. 7), while between ST year of birth and max ST 3 years after birth, the number of shared outliers were identified to *n* = 11 outlier loci (yielding the highest score in percentage (42%), followed by 36% (and *n* = 9 outliers) between ST year of birth and max ST 3 years before birth. The outliers detected were more or less evenly distributed on the different chromosomes, with an average between 0.4 and 3.1 outliers per LG for the environmental variables. Notably, some of the identified outliers were localized within the inversions, e.g., 21 and 10% of the detected outliers for ST year of birth and *F*_5.10_, respectively (for more information, see tables S22 and S23).

**Fig. 8.**
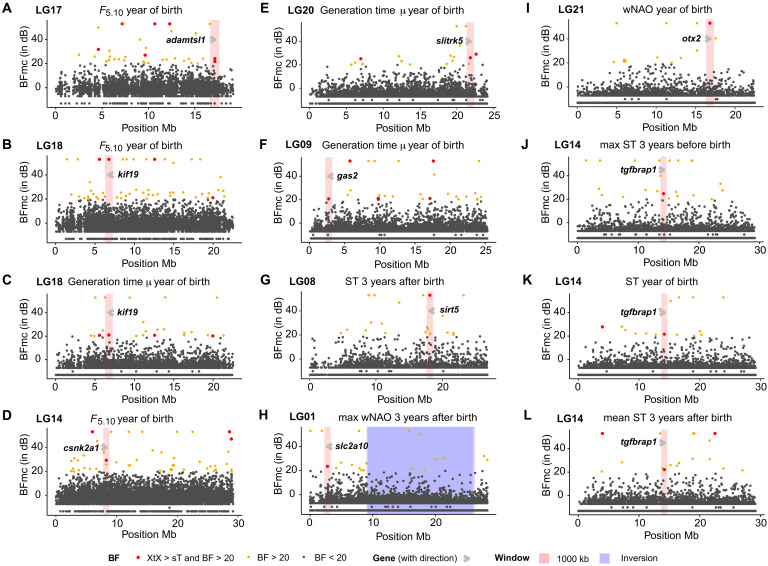
Outlier candidate genes associated with environmental variables. Variables given as ST (Kola Sea temperature), wNAO (winter North Atlantic Oscillation), *F*_5.10_ (fishing mortality), and μ (generation time). Significant outliers, with XtX > simulation threshold (sT) and BF over 20, are colored red. Genes marked with gray arrows and inversion and windows marked with blue and red, respectively, the latter ±500 kb from outlier site. (**A**) On LG17 two BayPass outliers associated with *F*_5.10_ yielded a nonsynonymous change inside an exon of *adamtsl1* (ADAMTS-like 1 gene). (**B** and **C**) On LG18, a synonymous outlier associated with *F*_5.10_ and μ was detected inside an exon of *kif19* (kinesin family member 19). (**D**) On LG14, an outlier associated with *F*_5.10_ was detected inside an exon of *csnk2a1* (casein kinase II subunit alpha). (**E**) On LG20, a synonymous outlier inside an exon of *slitrk5* (SLIT and NTRK-like family member 5 gene) was found. (**F**) On LG09, an outlier associated with μ was identified inside an intron of *gas2* (growth arrest–specific 2). (**G**) On LG08, an outlier inside exon of *sirt5* (sirtuin 5 gene), associated with ST 3 years after birth, was found. (**H**) On LG01, a nonsynonymous outlier associated with max wNAO 3 years after birth was detected inside an exon of *slc2a10* (solute carrier family 2 member 10). (**I**) On LG21, a synonymous outlier within an exon of the *otx2* (orthodenticle homeobox 2 gene) was identified, associated with wNAO at year of birth. (**J** to **L**) On LG14, an outlier associated with max ST 3 years before birth, ST year of birth, and mean ST 3 years after birth was identified inside an intron of *tgfbrap1* (transforming growth factor–β receptor–associated protein 1 homolog).

Of all the outliers detected, we here highlight those that could be coupled to gene predictions (localized within exons, in close vicinity to genes, i.e., introns and/or protein matches/mRNA matches/expressed sequence matches). For *F*_5.10_, two of the outliers detected on LG17 (see Fig. 8A) that were found in close vicinity to each other inside an exon of the ADAMTS-like 1 (*adamtsl1*) gene, yielding a nonsynonymous change from GAA (Glu, glutamic acid) to GGG (Gly, glycine), i.e., a functional shift from negatively charged to nonpolar. *adamtsl1* resembles members of the ADAMTS family of proteases (*64*) and suggested to have an important function in the extracellular matrix and/or controlling the synapse organization of the central nervous system (*65*). Furthermore, an outlier causing a synonymous mutation was detected on LG18 within an exon of the kinesin family member 19 (*kif19*) gene (Fig. 8, B and C), which was shared between *F*_5.10_ and generation time. The *kif19* gene is predicted to be involved in ATP hydrolysis activity and microtubule activity (*66*). One of the other outliers detected for *F*_5.10_ was localized on LG14 within an exon of the casein kinase II subunit alpha (*csnk2a1*) gene (Fig. 8D), which is one of three subunits forming the casein kinase II (CKII) tetramer known for its ability to phosphorylate growth regulatory proteins (*67*). However, since the reading frame was not given, functional evaluation of this shift was not possible. In addition, one synonymous outlier localized on LG20 inside an exon within the SLIT and NTRK–like family member 5 (*slitrk5*) gene (Fig. 8E) was associated with generation time. This is a gene that is predominantly expressed in neural tissues and has neurite-modulating activity (*68*). One outlier associated with generation time was found on LG09 (see Fig. 8F) within the intron of the growth arrest–specific 2 (*gas2*) gene, a gene associated with murine fibroblasts under growth arrest conditions, and mainly coupled to inhibition of cell division and/or apoptosis (*69*, *70*).

For mean ST 3 years after birth, we identified one outlier on LG08 that was localized within an exon of the sirtuin 5 (*sirt5*) gene (Fig. 8G), which is part of a larger gene family, i.e., the SIRT1–7 family, comprising seven evolutionary conserved enzymes that couple cellular NAD availability with health, nutrition, and welfare status in vertebrates (*71*). Also here, reading frame was not given, so no functional annotation was enabled. For maximum wNAO 3 years after birth, we identified one outlier that was localized inside an exon of the solute carrier family 2 member 10 (*slc2a10*) gene on LG01 (located outside the inversion; see Fig. 8H), yielding a nonsynonymous change GAG (Gln, glutamine) to CGG (Arg, arginine). The *slc2a10* gene encodes for a protein involved in maintaining glucose homeostasis (*72*). For wNAO, at year of birth, a synonymous outlier within an exon of the orthodenticle homeobox 2 (*otx2*) gene on LG21 (Fig. 8I) was identified. *otx2* is essential for forebrain and craniofacial development and crucial for photoreceptors formation and maintenance (*73*). Last, we highlight one outlier locus that was localized on LG14 (Fig. 8, J to L) within an intron of the transforming growth factor–β receptor–associated protein 1 (*tgfbrap1*) gene, which was associated with three ST environmental variables: max ST 3 years before birth, mean ST 3 years after birth, and ST year of birth. The *tgfbrap1* gene is involved in suppression of T cell activation, proliferation, and function, and thus protects organisms from inflammatory and autoimmune disease caused by an overexuberant immune responses (*74*). For full list of outliers in exons and untranslated regions see table S24; for all hits see https://figshare.com/articles/dataset/Supplementary_BayPass_hits/28151783?file=52219715.

## DISCUSSION

Our genome-wide retrospective characterization of the migratory NEAC, caught at its major spawning ground in northern Norway, uncovered varying degree of directional gene flow from the nonmigratory coastal cod throughout the last century. In addition, we identified that these genome-wide shifts were intensified in modern times (post millennium) accompanied with higher frequency of heterozygous individuals for the major inversions associated with migratory behavior in Atlantic cod (*29*, *38*, *39*), which is suggestive of increased hybridization events and/or survival of hybrids displaying higher degree of coastal cod ancestry. Further modeling and adaptive genome-wide scans indicated that these shifts were coupled to intensified fishing pressure, reduced generation time, and the ongoing ocean warming. Combined, our results spark earlier debates on how fisheries and other anthropogenic perturbations affect long-term genome-wide changes.

The significant increase of coastal cod ancestry detected at the genome-wide level demonstrates that long-term evolutionary level changes have occurred within the migratory NEAC over the past decades. Other studies have also reported on an ongoing admixture between the nonmigratory NCC and NEAC (*75*, *76*), but, to our knowledge, this is the first study that reports on a fluctuating directional introgression of coastal cod ancestry into the migratory ecotype, coupled to shifts in demographic and environmental variables. The positive *F*_s_’ values observed are further supporting these observations, i.e., the deviating allele frequencies are not induced by genetic drift alone, but likely stemming from increased introgression signals from coastal cod. This discrimination is mainly enabled by the unique sample collection: ranging from the early 1900s till present day, and thus spanning both colder phases (i.e., beginning of the century and in the 1960–70s) and warmer phases (in the 1940–50s and from the mid-1980s until today) (*77*), as well as contemporary samples of the nonmigratory ecotype found to co-occur with NEAC at the spawning grounds (*78*). Thus, the collection of samples, together with the outgroups from Canadian waters and the Baltic Sea, facilitated a solid variant calling of polymorphic sites to further evaluate if any temporal changes have occurred or not. Especially, inclusion of coastal cod samples was a necessity to accurately score the increased frequency of the coastal cod ancestry in the NEAC specimens and possibly an underlying reason why these genome-wide changes were not reported by Pinsky *et al.* (*6*). Moreover, our outlier analyses and adaptive genome-wide scans, i.e., *F*_ST_, AFD, _XP_EHH, and BayPass, further support subtle genome-wide allele frequency changes over the years investigated, whereas no larger genomic sweeps were detected. For the outlier analyses, some of the allele frequency shifts observed included loci associated to genes that could potentially affect phenotypic changes, including neural development coupled to behavioral differentiation (*zeb2*) (*56*), oxygen uptake in red blood cells (*tfrc*) (*60*, *61*), immune response regulation (*sema4b*) (*57*), and metabolism (*pex1*) (*59*). For the adaptive genome-wide scans, the outliers shown to covary with environmental and demographic variables were coupled to similar phenotypic alterations, e.g., synapse organization of the central nervous system and neural activity (*adamtsl1* and *slitrk5*) (*65*, *68*), and thus possibly linked to behavioral divergence, as well as immune response (*tgfbrap1*) (*74*) and metabolic (*kif19* and *slc2a10*) (*66*, *72*) regulation. In addition, other outliers detected were coupled to growth regulation (*csnk2a1* and *gas2*) (*67*, *70*, *79*), forebrain development, and crucial for photoreceptors formation and maintenance (*otx2*) (*73*). It should also be highlighted that some of the identified outlier loci were shared between the environmental and/or demographic parameters tested, including intensified fisheries and shifts in generation time. These findings combined indicate that the genomic alterations observed are linked to fisheries induced evolution, but then in a polygenic manner. However, some signals are most likely left undetected due to the low resolution in our dataset, having a rather low number of genome-wide SNPs compared to other datasets (*80*). This is often the case when working with historical samples, where the typically low DNA quality of the samples is hindering a higher sequencing coverage (*81*). A higher sequencing depth would most likely have detected a higher number of outlier loci in general, as well as a higher number of shared outlier loci. The other main study that has demonstrated such temporal polygenic changes in a marine fish species is the study on parallel phenotypic evolution in response to exploitation of the Atlantic silversides (*Menidia menidia*) (*8*). They uncovered small parallel allele frequency shifts among selection lines in unlinked genes associated with body growth variation, i.e., following classical quantitative genetic assumptions on how selection impacts complex traits (*82*). In addition, they detected rapid changes in frequencies for larger linked genomic regions, later on defined as inversions (*83*), for some of the selection lines, but not in all, demonstrating highly divergent genomic responses to the same selection pressure (*8*). Recently, another study reported on the evolution of a fast-growing piscivorous herring in the Baltic Sea (*80*), which is mainly differentiated from the other ecotypes at multiple smaller adaptive genomic regions including one of the larger inversions detected in herring, while at the genome-wide level, the discrimination is minimal (*80*).

In our study, the signals of coastal cod introgression were accompanied with an increase in HET individuals for the major inversion discriminating between the nonmigratory and the migratory ecotypes (*29*, *38*, *39*), from the millennium and onward. These results combined could indicate that the two ecotypes are interbreeding more frequently nowadays than historically. For Atlantic cod, fine-scale selection of spawning location seems to be affected by ambient temperature (*28*, *84*) and linked to gonadal development (*85*), embryonic/larval ontogeny (*86*), and/or indirectly food availability (*87*, *88*). Earlier characterization of the spawning distribution at the major spawning ground in the Lofoten area has uncovered that during specific years (with optimal temperature and saline conditions), higher aggregation of eggs has been observed inside Vestfjorden over shallow bottoms coupled to a narrow strip of cold (and low saline) water along the east coast of the Lofoten islands (*28*). While under suboptimal conditions or when the area with optimal conditions is too narrow, later arriving fish (at least after the massive increase in SSB in the 2000s) more often occupy spawning sites on the outside of the islands, i.e., on the continental shelf (*28*). Together, environmental factors like wind force, the depth of the thermocline (*89*) at the spawning ground, and demographic variables, including SSB, could affect the level of interaction between the ecotypes and thus the likelihood of hybridization events. Here, we can highlight the increased signals of coastal cod ancestry observed in NEAC at the beginning of this century, uncovering that the degree of hybridization (i.e., gene flow) between the ecotypes is not static. In the early 1900s, the estimated SSB was rather high (*90*), and thus, could potentially have affected the degree of interaction between the two ecotypes. Moreover, the higher proportion of HETs could also indicate that the offspring had a higher survival, even if spawning inside the fjord at the historical fishing grounds, most likely due to the less intensified fishing pressure during this time period (*90*).

Notably, the observed increase in the number of HET individuals in modern times, however, does not need to result from a higher degree of hybridization events alone. The increase could also be explained by enhanced survival of HET versus homozygous DERIVED individuals due to (i) behavioral differentiation and thus, likelihood of being fished at the spawning ground (as mentioned above) and/or (ii) due to any fitness advantage during the e.g., ongoing ocean warming, resulting in higher survival rate of HETs in more recent years. For instance, if the HET individuals are more prone to spawn in areas in close vicinity to the shore inside Vestfjorden or at continental shelf, but at shallower depths, this could indeed enhance these individuals’ likelihood of surviving due to behavioral differentiation, by aggregating at areas that larger trawling vessels do not target (*91*). For the fitness advantage scenario, there are several reports demonstrating how HET individuals have increased fitness compared to homozygous individuals (*92*), due to, e.g., heterosis and/or due to enhanced survival/fitness. The latter hypothesis is supported in a study by Case *et al.* (*93*) showing that larval Atlantic cod being heterozygous (*ab*) for the *pan* I locus, displayed larger growth rate versus homozygous (*bb*) individuals, when reared under seminatural warmer mesocosm conditions. Here it could be mentioned that the *pan* I locus is situated close to the breakpoint on the major inversion on LG01 (*38*), and has been used for decades to discriminate between the two ecotypes of Atlantic cod, i.e., the migratory NEAC and the nonmigratory NCC as well as the coastal and frontal cod found in Icelandic waters (*94*). Thus, *pan* I genotyping conducted in previous studies (as well as within this study) can be used as inversion scoring of the LG01 inversion (see the Supplementary Materials for more details), where NEAC exhibit high frequencies of the *pan* I *bb* genotype versus NCC exhibit high frequencies of the *pan* I *aa* genotype.

When using the GAM modeling approach, we found support for the inversion frequency shifts observed, especially the major inversion on LG01 but also for the inversion on LG02 and LG07, to be tightly coupled to intensified fisheries and climatic variables, i.e., increasing sea temperature and a positive wNAO index (see text S6). These results strongly indicate a correlation between the inversion genotype and survival based on fitness and/or behavioral differentiation as discussed above. In short, a positive NAO index phase results in increased westerly winds over the North Atlantic, which again increases the Barents Sea water temperatures through enhanced volume flux of relative warm Atlantic water from the southwest, higher air temperatures, and increased cloud cover (*95*). Higher Barents Sea water temperature could potentially improve growth and survival of cod larvae, through the vulnerable stages when year-class strength is determined (*96*, *97*), both directly through faster development rates and indirectly through regulating the production of their main prey, nauplii of the copepod *Calanus finmarchicus* (*98*). In addition, increased inflow from the zooplankton rich Norwegian Sea further increases availability of food for the cod larvae (*96*). Together, years with warmer water and higher food availability (for larval and juvenile fish) could result in higher growth rates and greater survival for certain genotypes, and thus, by such impact, the genomic signatures of the different cohorts as detected by our temporal dataset. For instance, the decrease in hemoglobin Val-Ala genotypes, two nonsynonymous SNPs coupled to cold water adaptation in Atlantic cod (*35*), indicates higher survival of HET individuals in warmer years. Here, it can be noted that the hemoglobin genes are located on LG02, but outside of the detected inversion. The similar response for the ANCESTRAL LG02 inversion genotype and the cold water–adapted hemoglobin genotype indicates a potential linkage between the inversion and the hemoglobin genotype.

Furthermore, for the inversion on LG01, a decrease in frequency of the DERIVED variant (and thus, an increase in number of HETs) was also linked to shorter μ generation time. Intriguingly, a direct coupling was also shown for the individual genotyped data, where age at maturation defined by otolith readings were significantly affected by inversion type, i.e., being homozygous for the DERIVED variant showed higher age at maturation, than for those being scored as HET or as homozygous for the ANCESTRAL variant (mainly found in coastal cod). Thus, our findings further support earlier reports and the suggested association between the documented decrease in size and age at maturation and anthropogenic perturbations, i.e., intensified fisheries (*4*, *14*) and ocean warming (*15*), and that this response is linked not only to phenotypic plasticity but also linked to genomic variation, such as shifts in inversion frequencies (*31*, *32*).

The evolutionary importance of the inversions over the time period investigated was further confirmed by the selection analyses conducted. For the migratory ecotype, elevated Tajima’s D values inside versus outside of the inversion on LG01 were prominent for almost all time periods investigated, suggesting that this inversion is under balancing selection (*99*, *100*). However, lowered values were detected inside versus outside of the inversion on LG07, indicative of being under directional selection (*99*, *100*). For the nonmigratory ecotype, opposite signals were detected, where the inversions on LG02 and LG07 show signs of elevated Tajima’s D values, i.e., being under balancing selection, whereas the inversion on LG01 show signals of lowered values and thus being under directional selection. Last, it should be noted that some slight differentiation in signals of selection for the different time points/years investigated were uncovered, indicating that the genomic signature (and the linked genotypes) over an inversion is not static and that changes for a specific inversion genotype can occur.

By taking advantage of whole-genome sequencing combined with historical datasets and statistical modeling, we here show that the iconic migratory Atlantic cod ecotype has undergone genome-wide changes over the past century, via increased introgression signals from coastal cod in response to intensified anthropogenic perturbations. However, we cannot exclude that some of the genomic alterations observed could be associated to e.g., shifts in age structure or by alteration in the composition of the spawning component of NEAC in the Lofoten area. This scenario is less likely, since it insinuates that there is genomic differentiation coupled to age and/or subpopulation structuring within the larger NEAC population, where gene flow seems prominent.

For future forecasting, it will be instrumental to further evaluate which factors affect the survival of the hybrids the most, i.e., is it the fitness advantage of the hybrids or is it increased survival due to behavioral differentiation, e.g., avoidance of being caught at the spawning grounds. Such knowledge would aid in defining the correct management actions to avoid depletion of important genomic variation from one of the most important marine resources in the northern Atlantic Ocean.

## MATERIALS AND METHODS

### Sample collection

Historical samples, i.e., cod scales or otoliths, from Atlantic cod specimens caught at the spawning grounds in the Lofoten area, from selective years during the last century (see tables S25 to S27 and the Supplementary Materials for more details), were obtained from the Institute of Marine Research (IMR), Norway. The samples were selected from years based on the Barents Sea Climate index (*37*), including both cold (1907 and 1975) and warm periods (1940, 1958, 1999, 2011, 2012, and 2014). In addition, modern samples from the same location, the Lofoten area, were included. From the modern collection, both ecotypes were included, i.e., the migratory NEAC (caught during spawning) and the nonmigratory NCC. For the historical collection, the majority of the samples used were assigned as NEAC specimens. The discrimination of the two ecotypes was based on otolith readings and the differentiation in growth pattern deposited, mainly due to differences in feeding and migratory behavior between the ecotypes (*101*). In parallel, age determination (for the NEAC and NCC used within this study) was conducted. Both analyses were performed by IMR (*101*).

As outgroups and scaling of genetic variation, we also included modern samples from the NOR, BOR, and GOM (see table S25 and the Supplementary Materials). For all samples, we strived to achieve an even sex distribution and a representative length distribution for the sample collections for the different years and/or locations included in our study (see text S7 and fig. S14).

All samples used in this study were collected in a responsible manner (both the historical and modern samples): (i) in connection to research surveys (as part of larger hauls for stock assessments) or (ii) by commercial fisheries (obtained as by-product of conventional business practice). The fish were humanely euthanized before sampling in accordance with the guidelines set by national and international animal welfare laws (e.g., https://norecopa.no), and thus no specific legislation was needed.

### Whole-genome sequencing and data processing

DNA extractions and subsequent library preparations for the historical samples (1907–1999), were conducted at the Sensi-Lab at the Natural History Museum of Oslo, University of Oslo, Norway (for details see text S8). A modified protocol, specialized for DNA extraction of historical DNA (*102*) was used. The genome sequencing was performed by the Norwegian Sequencing Centre (https://sequencing.uio.no), University of Oslo, Norway using the HiSeq2500 System (Illumina). The mean sequencing coverage obtained for the different samples was 9.2× for the samples from 1907 (scales), while 5.6× of that for the samples from 1940 to 1999 (otoliths) (for more information, see Table 1). Moreover, the modern samples were extracted, prepped, and sequenced according to the description stated in Pinsky *et al.* (*6*), yielding a mean sequencing coverage of 9.2× (for more information, see https://figshare.com/articles/dataset/Supplementary_coverage/28190465?file=52218728). However, because of the rather high coverage detected in two of the modern samples, i.e., 17× and 35×, respectively, they were downsampled to 7× coverage, before any further processing. For more in-depth details on the data processing and quality assessment, see text S9 and fig. S15.

**Table 1.** Sequencing overview. Summary of the sequencing statistics reflecting the ecotype and/or population, location, tissue type used, sex ratio (males/females and NA excluded), sequencing coverage ± SD, number of samples sequenced, and number of samples left after filtering in WGS dataset.

Ecotype/population	Year of capture	Place of catch	Tissue type	Sex M/F	Coverage nuclear DNA	Number of samples sequenced	Number of samples after filtering in nuclear
NEAC	1907	Lofoten, Norway	Scales	10/16	9.2 ± 1.9	28	27
	1940	Lofoten, Norway	Otoliths	11/10	5.6 ± 0.9	23	21
	1958	Lofoten, Norway	Otoliths	7/8	5.4 ± 1.8	23	15
	1975	Lofoten, Norway	Otoliths	3/8	3.8 ± 2.0	19	11
	1999	Lofoten, Norway	Otoliths	8/3	8.5 ± 5.0	14	11
	2011	Lofoten, Norway	Tissue	7/15	7.8 ± 1.3	22	22
	2014	Lofoten, Norway	Tissue	12/14	9.8 ± 1.9	26	26
NCC	1958	Lofoten, Norway	Otoliths	6/3	0.4 ± 0.3	9	0
	1975	Lofoten, Norway	Otoliths	3/6	0.3 ± 0.2	9	0
	2011	Lofoten, Norway	Tissue	14/12	7.9 ± 0.8	26	26
	2014	Lofoten, Norway	Tissue	11/13	10.8 ± 5.6	25	25
**NOR**	2002	NOR	Tissue	9/6	8.6 ± 0.7	15	15
**BOR**	2012	Bornholm, Baltic Sea	Tissue	8/7	9.2 ± 2.0	15	15
**GOM**	2009	Gulf of Maine	Tissue	NA	9.9 ± 0.8	15	14

### Variant calling and filtering

Haplotype and genotype calling for the genome-wide dataset were conducted using GATK (version 3.8) for the mt genome, and for two different versions of the WGS dataset, the following were used: (i) the full dataset where all years were included (*n* = 241) and (ii) the reduced dataset where data from 1940 and 1958 were excluded (*n* = 199). The motivation behind this exclusion was to reduce the potential bias in the variant calling due to the shorter read length for those years, specifically 1958 (fig. S16). Read length summaries were obtained using SAMtools view version 0.1.19 (see text S10 and fig. S16). For the WGS data, mt contamination analysis was performed before final genotype calling and samples with <1× nuclear coverage excluded (see text S11 and S12, fig. S17, and tables S26 and S27).

The subsequent 13 to 14 filtering steps for the genome dataset were performed using a combination of BCFtools (*103*) (version 1.1) and VCFtools (*104*) (version 0.1.14); see text S12 for in-depth details. In short, the filtering included restriction to bi-allelic sites, removal of sites with low DP (sample specific read depth), and removal of individual samples and sites with high percentage of missing data. Correction was made for mappability (*105*), and care was taken to exclude repetitive regions and removal of sites with C > T and G > A. Removing repetitive regions and sites of low mappability is important when having short read lengths (*105*, *106*).

For the variant calling of the mtDNA, the data were mapped toward the mt genome launched together with gadMor2. The ploidy was set as either two (for the contamination analysis, see text S11 for more details) or as one for the variant calling performed for subsequent mt genome analyses. Here, in this dataset, we included 18 historical coastal cod (NCC) samples from 1958 and 1975, which were whole-genome sequenced, but the coverage was too low to include in the WGS analyses (see table S27).

### Temporal population genomic differentiation

For assessment of overall fine-scale temporal population structure of NEAC specimens collected over the last century, “neutral” genome-wide datasets were generated by (i) pruning for linkage with plink (1.90b5.2) and (ii) excluding the known inversions, present on LG01, LG02, LG07, and LG12 (*29*, *30*, *33*), using the updated breakpoint regions defined for this dataset (see *F*_ST_ analyses below and texts S13 and S14, figs. S18 to S20, and tables S28 and S29 for more details). The PCA analysis was made with Eigensoft Smartpca (*40*, *41*) (version 7.2.1). Plink settings and Eigensoft settings were set to allow extra chromosomes (chromosome set as 23 and no sex chromosomes). The analyses were conducted on both datasets, i.e., the reduced dataset (excluding 1940 and 1958 with the total number of individuals *n* = 192) resulting in a total number of SNPs *n* = 141,549, and the full dataset (including 1940 and 1958 with the total number of individuals *n* = 228) resulting in a total number of SNPs *n* = 70,405.

For the admixture analyses (using ADMIXTURE version 1.3.0), we used the neutral SNP datasets defined above (pruned for linkage and excluding the inversions). Cross-validation (CV) of the number of divisions was run up to *k* = 10. However, on the basis of the CV estimates, which were rather low and more or less similar for *k* = 1, *k* = 2, *k* = 3, *k* = 4, and *k* = 5, i.e., ranging from 0.381 to 0.401, the genome-wide ancestry fractions (see figs. S2 and S3), and the theoretical reasonable number of populations present in the dataset (GOM, NOR, NCC, NEAC, and BOR), we here present the admixture analyses at *k* = 5. In this analysis, all individuals from the modern material were included, as well as those with uncertain otolith types, i.e., migratory ecotype caught during summer and bycatch of coastal cod during the spawning season. However, for the calculations of the ancestry fractions, only done for NEAC, we excluded those individuals (i.e., bycatch or straying cod). Differences in coastal cod ancestry between time periods (i.e., catch year and year of birth) were examined using Kruskal-Wallis rank-sum test. For these tests, impact of missingness was taken into account for the full dataset (for more information, see text S2).

The above-mentioned neutral SNP datasets were also used to investigate if any temporal changes in *N*_e_ of the NEAC population over the past century were detected. For the reduced dataset, temporal changes in allele frequencies for the different sample intervals (e.g., 1907–1975, 1907–1999, etc.) were calculated as averages over the 141,549 SNPs, using the *F*_s_ estimator (*43*). The actual sample size is under the assumption to be of much smaller size than population census size, following sampling plan II (*44*), and thus, based on this, the estimated *F*_s_ were adjusted for sampling accordingly, i.e., to equation 13 in (*43*). The SE for point estimates of *F*_s_’ were obtained by Jack-knifing over loci and 95% confidence limits for *F*_s_’ calculated as mean *F*_s_’ ± 1.96 SE. For the full dataset, see details in text S3. To investigate the clustering of maternal lineages, an mt haplotype network was inferred for the full dataset and the additional historical coastal cod individuals (see text S4 for details)

### Inversion and hemoglobin genotyping

For the inversion genotyping of the well-known inversions detected in Atlantic cod on LG01, LG02, LG07, and LG12 (*29*, *38*, *39*), we used the above-mentioned WGS data and already genotyped individuals, i.e., from a 12K Illumina SNP chip (*29*, *32*) or a 48 Fluidigm SNP array (*25*), improving the statistical power in the subsequent retrospective inversion analyses. Following an inspection of the genome-wide differentiation from 12K SNP chip data (see text S15 and fig. S21), we included data from 64 individuals caught in the Lofoten area in 2011 and 2012 (for more information, see table S25). Moreover, based on data from the 48 SNP array, we enabled inclusion of the inversion genotypes from additionally 35 individuals caught in Lofoten in 2014. For the genotyping of the inversions, we used the same SNPs as in the previous study by Langangen *et al.* (*25*) with the exception of LG01. For LG01, we identified a discrepancy in the inversion scoring conducted when using the selected SNPs (*25*) and the results that we got from the WGS data (for those where overlapping data exists), so here we instead used the *pan* I locus for the genotyping for the inversion on LG01, which were found to have high degree of consistency with the WGS data. For the two datasets mentioned, both NEAC and NCC ecotypes were included, defined by otolith readings (for more information, see table S25).

For the WGS data (not pruned for linkage), the inversion genotypes were scored by PCA analyses, plotted with Eigensoft Smartpca (version 6.1.4). The readjusted inversion breakpoints were used to secure that all sites were positioned well within each inversion (see text S14, table S29, and figs. S19 and S20). The distinction of the three well-defined clusters representing the two homokaryotypes and the heterokaryotypes, i.e., the ANCESTRAL, HET, and DERIVED arrangements, as previously described in Mérot (*107*) and defined by Matschiner *et al.* (*31*), were further validated by plotting the PC1 against percentage of HET sites using VCFtools (version 0.1.4); see figs. S22 and S23. Genotype frequencies (*f*_Genotype_) were calculated for the different years for both NEAC and NCC, only including individuals of NEAC caught at the spawning ground (representing the spawning population), and NCC caught outside of the spawning season.

In addition to the inclusion of modern data genotyped using the 12K Illumina SNP chip or the 48 Fluidigm SNP array (*25*), we also managed to retrieve the inversion scoring for some of the historical samples. For the excluded individuals from year 1940 and 1958, due to high degree of missingness for the WGS analyses, a proper inversion scoring was conducted using a VCF file (Step2) with only the first steps of basic filtering included (and keeping indels) and at Step4 (only basic filtering performed). The scoring for the additional historical samples was conducted by PCA analyses (Step4 VCF) for all inversions (as described above, see figs. S24 and S25) combined with inspections over the inversions (Step2 VCF) using IGV (integrative genomics viewer) and Genotype Plot (*108*, *109*) (see text S15 and figs. S26 to S29). For confirmation, in-depth inspections of the genotypes (banding patterns) over specific genes located inside of the inversions were conducted, i.e., panthophysin for LG01 (fig. S26), collagen-alpha-1 for LG02, aquaporin-11 for LG07, and G protein signaling modulator 2 loci for LG12 (fig. S27 to S29).

For the hemoglobin genotyping a VCF file at Step2 (text S6) for the full dataset, without filtering indels, was manually inspected in IGV to search for the two SNPs located in positions corresponding to Met^55^Val and Lys^62^Ala in hemoglobin β1, two nonsynonymous substitutions that have previously been linked to temperature adaptations (*35*, *36*). Upon identification, the VCF was loaded, the two SNPs extracted, and their allele frequencies were plotted using R version 3.6.3 with packages vcfR and Tidyr. The file at Step2 was used since the locus is an indel (*35*).

For testing of frequency shifts over the time period, in terms of inversion and hemoglobin genotypes, Fischer’s exact test with Bonferroni correction were performed (see tables S11 and S16). In addition, we tested for HWE with a χ^2^ goodness-of-fit test for each year (see tables S12 to S15, S17, and S18).

### Detection of selection

For the detection of signatures of selection along the chromosomes, Tajima’s D and π were calculated using a less filtered dataset (i.e., without pruning and MAF filtering) that included the inversions. Tajima’s D and π estimates were calculated by 30- and 50-kb window sizes, respectively, and step size set as half of window size using VCFtools (version 0.1.14). The statistics were calculated per year for NEAC and NCC, as well as for the outgroups separately. Since these analyses are sensitive to inaccurate genotype calls, we here used the reduced dataset (excluding the years 1940 and 1958).

Additional selection analyses, i.e., iHS and _XP_EHH, were performed on a phased version of the reduced dataset (without pruning and MAF filtering), including the inversions using rehh (*110*). Phasing was achieved using a combination by VCFtools version 0.1.14 and BCFtools version 1.1 (splitting VCF by chromosome and providing chromosome map) and plink version 1.90b5.2 (making bed file) and Shapeit (*111*) version 2.r837 (https://mathgen.stats.ox.ac.uk/genetics_software/shapeit/shapeit.html) for the phasing (--window 0.5 -T 5). For confirmation, the selection analyses (Tajima’s D, π, iHS, and _XP_EHH) were also performed for the DERIVED and ANCESTRAL genotype for the inversion on LG01 separately for the different years, to avoid selection signals due to AFDs. Because of the limited number of individuals harboring the ANCESTRAL genotype in NEAC, the analyses were only conducted for NCC and vice versa for the DERIVED genotype only conducted for NEAC for the different years.

### Modeling approaches using demographic and environmental variables

By taking advantage of the historical dataset spanning the last century, temporal shifts in proportion of different genotypes (ANCESTRAL and DERIVED) for all of the inversions and proportion of homozygous Val-Ala (or alternative genotypes) at the *hb* β*1* locus at LG02 were looked into in more detail, for NEAC specifically. For this, we back calculated year of birth for all the NEAC individuals, based on the otolith and scale readings combined with year of catch.

Using a GAM modeling approach, via the mgcv library, the temporal variability in these proportions were related to the demographic explanatory variables, i.e., SSB, *F*_5.10_, population growth (*r*) or generation time (μ) as well as environmental variables, including ST and wNAO. For the two latter, we considered the mean and maximum of the three years before birth (affecting the parents, i.e., year of birth and 2 years before) and after birth (affecting survival during the critical periods of life that are the years before recruitment, i.e., year of birth and 2 years after). Data for environmental and demographic variables are described in detail in text S6. The dependent variable was set as proportion with a binomial error distribution. In addition, we took into account that the proportions could be calculated on an uneven number of fish. Since the GAM procedure automatically selects the degree of smoothing based on the Generalized Cross Validation score (a proxy for the model’s predictive performance), it can shrink the smooth term toward a linear model if appropriate. This was tested and confirmed by comparing our model with its linear equivalent using quasi-AIC (see table S30).

Last, we took advantage of the individual recordings obtained for the NEAC included in the study to describe the individual inversion genotypes relative to individual demographic variables, i.e., age at maturation, growth rate, and Fulton K (all perceived from a combination of the otolith readings and length/weight measurements), using the function boxplot. For age at maturation, we had data for all years, while for growth rate and Fulton K, years 1958 and 1975 were excluded due to the lack of weight recordings those years. The significance was estimated using a Kruskal-Wallis rank test for stochastic dominance among groups followed when a significant difference is observed by a post hoc test between pairs using a Dunn’s test, where the *P* values for multiple comparisons are adjusted using the Benjamini-Hochberg adjustment using the package dunn.test (*112*) in R. The dunn.test function accounts for tied ranks. All analyses were conducted using the software R version 4.1.3.

### Outlier analyses

Weighted pairwise fixation index *F*_ST_, as a measurement of genetic differentiation, was assessed using VCFtools (versions 0.1.14 and 0.1.16). Candidate regions under positive selection were identified using a combination of standard population genetic statistics and permutation tests. First, allele frequencies, locus-specific and window-based *F*_ST_ values between populations, were calculated with VCFtools. Weighted *F*_ST_ was calculated using 30-kb windows and a step of 15 kb. Significance of the *F*_ST_ values, *P* values, were estimated through permutations for each chromosome using the permuteSmooth function in vcflib (*113*), using the *F*_ST_ values, window based and locus specific, and allele frequencies calculated with VCFtools. To correct for multiple tests, *q* values were subsequently calculated per chromosome using the q-value package (*114*) in R. Windows with a *q* value less than 0.05 were considered as candidate regions under positive selection. Furthermore, from the *F*_ST_ comparisons between the modern NEAC and NCC (as well as the other outgroups), the inversion breakpoint regions were re-evaluated and adjusted (for more details, see text S14).

For confirmation of the identified outlier regions, additional outlier tests were conducted, i.e., _XP_EHH analyses (described in detail above) and AFD analyses. However, since these analyses are sensitive to accurate genotype calls, we here used the reduced dataset (excluding the years 1940 and 1958 for the selection analyses) only. In short, *F*_ST_ outliers, with *q* value less than 0.05, found to be common between a minimum of two-three of the pairwise comparisons between the different years were further looked into. We investigated if some of these sites were overlapping with regions/sites found to be significantly different in the _XP_EHH comparisons [i.e., log(*P*) > 3] as well as within the 99.9th quantile of the identified AFD. Furthermore, the functional importance of these SNPs was inspected via IGV (in 30-kb windows) using the annotated filtered gff-file for gadMor2 (*115*), which can be found at: https://figshare.com/articles/dataset/Transcript_and_genome_assemblies_of_Atlantic_cod/3408247.

In addition, we performed genome-wide scans for the reduced WGS dataset using BayPass (version 2.41) (*62*, *63*), which allows for identification of outlier loci that covaries with the environmental and demographic variables described above and in text S6. BayPass was run with the instructions described by Stuart (*116*) with some modifications. We exclusively looked at NEAC using the same year classes as in the ADMIXTURE analysis (excluding year classes with less than two samples). For the env file, we used ST at year of birth, mean and max ST 3 years before and after birth, *F*_5.10_ (at year of birth), wNAO (at year of birth, maximum 3 years before and after birth), and generation time (at year of birth). For ST and wNAO, data were available for all time points included in our study, while for *F*_5.10_ and generation time, data were available from 1913 and onwards (see text S6). On the basis of this, the number of individuals and groups was reduced from 87 individuals and eight groups to 64 individuals and five groups for the testing conducted for *F*_5.10_ and generation time data (see https://figshare.com/articles/dataset/Supplementary_data/28191122?file=52218356). Five replicate runs were performed for each variable as recommended for MCMC, median BF mc (Bayes Factor under the AUX model), XtX statistic (*117*) and β coefficient were calculated. Only the outliers that passed the filtering thresholds were kept, i.e., BF > 20 and passing XtX 1% (99th quantile) simulation threshold. The identified outlier loci (found to significantly covary with the environmental and demographic data) were further inspected in IGV using the above mentioned gff-file.

## Supplementary Materials

### This PDF file includes:

Supplementary Text Figs. S1 to S29 Tables S1 to S30 References
