# Supplementary material for: A century of anthropogenic perturbations impact genomic signatures of the iconic migratory Atlantic cod
Source: Sci Adv. 2025 Jul 30;11(31):eadp3342. doi: 10.1126/sciadv.adp3342 (PMC12309680; doi:10.1126/sciadv.adp3342)
Supplement: Supplementary file 1 — Supplementary Text Figs. S1 to S29 Tables S1 to S30 References [file sciadv.adp3342_sm.pdf]

Science Advances
AAAS

Supplementary Materials for

A century of anthropogenic perturbations impact genomic signatures of the iconic migratory Atlantic cod

Cecilia Helmerson *et al.*

Corresponding author: Cecilia Helmerson, cecilia.helmerson@ibv.uio.no; Sissel Jentoft, sissel.jentoft@ibv.uio.no

Sci. Adv. **11**, eadp3342 (2025)
DOI: 10.1126/sciadv.adp3342

This PDF file includes:

Supplementary Text
Figs. S1 to S29
Tables S1 to S30
References

Supplementary Text

S1 PCA analyses and statistics

Similar differentiation between the populations included in the PCA analyses were detected in both datasets. For the full dataset (i.e., including 1940 and 1958), the first three principal components had significant Tracy-Widom statistics, and the percentage of variation explained ranged from 0.62% to 3.44%. For the *reduced dataset* (i.e., excluding 1940 and 1958), the first three principal components were significant as well and variation explained ranged between 0.65% to 3.39%.

Eigbest SNPs, driving separation in PCA are found in **table S3** and **table S6**.

Some Eigbest SNPs were found also in XPEHH (Cross Population Extended Haplotype Homozygosity) (*53*) (see **Supplementary html-file**). For example, the one of the sites driving PC2 in the *reduced dataset* LG02:3761996 also corresponds to a XPEHH outlier between 1999 and 2014 NEAC.

S2 Admixture analyses and statistics

The random seed was set to 43, for both the *full* and the *reduced datasets*. The separation of GOM and BOR, is found for both the *full* and the *reduced dataset* (see **fig. S2-S3**). However, for the next level (k=4) the two datasets start to diverge. For the full dataset (including 1940 and 1958) the NCC and NOR display a more or less similar ancestry pattern vs. the NEAC, whereas for the *reduced dataset* (excluding 1940 and 1958), the NCC separates out without the NOR, which are more akin to NEAC. At k=5, the *reduced dataset*, the five groups separating out are GOM, NOR, NCC, NEAC and BOR, while in the *full dataset*, NOR does not separate out, instead a historical signal is detected, and it is the NEAC from year 1958 that separates out the most. This differentiation is most likely due to the difference in SNPs obtained for the two datasets.

To ensure the quality ADMIXTURE analyses in terms of degree of missingness (in the historical material) and the scoring of coastal cod ancestry we did some additional testing. Due to some individuals having a coastal cod ancestry fraction equal to 0 we used a statistical hurdle model (*121*) approach using *glmer* function from the *lme4* package (*122*). The first model used was a presence-absence generalized linear mixed-effects model, with year as random effect and a binomial distribution (0 when the coastal cod fraction was equal to 0 and 1 when the fraction was different from 0). The second model used was generalized linear mixed-effects model with year as random effect modelling the coastal cod ancestry fraction ignoring all individuals with a fraction level equal to 0 with a quasipoisson distribution to account for the overdispersion in the residuals. For the *reduced dataset* we found that missingness did not impact the coastal cod ancestry fraction ($P>0.05$ for both the binomial and the quasipoisson models). For the *full dataset* we found that missingness did not impact the detected level of coastal cod ancestry in the binomial model ($P>0.05$), whereas for the quasipoisson model the fraction level detected were seemingly linked to degree of missingness ($P<0.05$), i.e., where individuals with higher degree of missingness displayed lower levels of coastal cod ancestry. Based on this, for the *full dataset* we excluded individuals with >30% missingness. Subsequently, we proceeded by testing differences in coastal cod ancestry (for both datasets) between i) catch years as well as ii) year of birth (with a pooling of 3 year classes together) using Kruskal Wallis test. For the *full dataset* we identified significant differences in coastal cod ancestry between catch years (Kruskal-Wallis chi-squared = 22.414, df = 6, *P* value = 0.001018) as well as for year of birth (Kruskal-Wallis chi-squared = 27.715, df = 14, *P* value = 0.01552). For the *reduced dataset* coastal cod ancestry was significantly different

between catch years (Kruskal-Wallis chi-squared = 12.196, df = 4, *P* value = 0.01595) as well as for year of birth (Kruskal-Wallis chi-squared = 27.482, df = 8, *P* value = 0.0005834).

S3 Ne and temporal shifts

Average temporal shifts in allele frequencies were estimated for each sample-pairs using the unbiased estimator F_s (*43*). Briefly, using a thinned (to minimize LD) set of 70405 SNPs, the method sum over SNPs their squared allele frequency differences between two sample occasions, standardized by dividing by the sum over SNPs of their expected heterozygosity, z*(1-z), given the mean allele frequency z in the two samples (eq. 9) (*43*). The resulting quantity, denoted F_s, is then adjusted for bias arising from sampling finite numbers of individuals for genetic analyses, by subtracting terms of the inverse harmonic mean sample sizes (eq. 13) (*43*), yielding a putatively unbiased estimate F_s'. Standard errors (SE) for F_s' (eq. 11.5) (*123*) were obtained by the jack-knife approach, leaving one SNP out at a time and calculating jack-knife replicates of F_s'. These SE were used to construct standard normal 95% confidence intervals (CI) for the F_s' estimates.

S4 mt network analysis

First, individual fasta sequences were extracted from the VCF file using PPP (*124*) v0.1.13. Then, protein coding genes (PCGs) were identified in the gadMor2 mitogenome using MitoFish (*125*, *126*). The PCGs identified from the gadMor2 reference were used to extract the corresponding PCGs from the individual fasta sequences using BLASTN (*127*). The extracted individual PCGs were then aligned with MAFFT (*128*) v7, manually corrected for length, and the *nd6* gene was reverse complemented as it is encoded on the light strand. The aligned PCGs were concatenated, and a maximum likelihood (ML) analysis was conducted using IQ-TREE (*129*) v2.2.0, with the substitution model selected using ModelFinder Plus (*130*). Finally, the resulting ML tree and alignment were used to infer a haplotype network using Fitchi (*131*).

The Canadian samples (GOM) were found in separate clusters, with only a few modern and historical NEAC present. In total, 79 nodes were identified and 78 edges. The largest distance observed in the mt network was 109 substitutions. BOR and 1958 NCC were present in the fewest number of nodes, and 2014 NEAC being most prevalent in the largest number of nodes followed by 2014 NCC. Thus, no specific clustering was detected based on this network analysis, which is in concordance with earlier reports (*132*), and further confirms high degree of gene-flow between the NCC and NEAC throughout the last century.

S5 Temporal inversion and haemoglobin frequency analysis

The significant differences detected for the inversion on LG01, using Fischer´s exact test with Bonferroni correction, were found between the pairwise comparisons of year 1907, 1940, 1958 and 2012 vs year 2011 for NEAC (see **table S11**). Four additional comparisons were almost significant, 1975 versus 2011 and 1958 versus 1975, 2012 and 2014, but failed due to the Bonferroni correction (see **table S11**). Deviations from HWE were uncovered for year 1907 and 2012 for LG01 (*P*=0.008 and *P*=0.001, **table S12**), 2012 for LG02 (**table S13**) and LG12 (*P*=0.006, *P*=0.036, **table S15**). For coastal cod no significant differentiation between the two years (2011 vs 2014) for any of the inversions (LG01, LG02, LG07, and LG12) were detected (see **table S11**).

S6 Modelling approaches using demographic and environmental variables

In the modelling approaches both environmental and demographic variables were used. This section describes the data used for the variables, starting with the environmental variables and then the demographic variables.

Environmental variables

Climate indices, such as the North Atlantic Oscillation index for the winter months (wNAO) December through March (*47*)), capture complex spatio-temporal variability into a simple metric and integrate larger scale climate processes and their variability (*133*). They have been shown to be good predictors for biological processes (*134*). In the Barents Sea, wNAO was documented affecting different components of the system such as Northeast Arctic cod (*135*). The wNAO index is based on the difference of normalized sea level pressure (SLP) between Lisbon, Portugal (high pressure) and Stykkisholmur/Reykjavik, Iceland (low pressure) since 1864 (*47*). Positive NAO phase characterized by a pronounced difference in SLP. During positive NAO phases, the westerly winds are strengthened and moved northward, causing increased precipitation and temperatures over northern Europe. Roughly opposite conditions occur during the negative-index phase. Positive NAO phases affect the Barents Sea through increasing volume flux of warm water from the southwest, cloud cover, and air temperature, all leading to increased water temperature, which influences fish growth and survival both directly and indirectly (*136*).

This effect on individual growth is evident in Arcto-Norwegian cod (*Gadus morhua*) with warm years (positive NAO) favouring higher growth rates (*87*). In an analysis of 22 cod stock, it was shown that NAO affects cod recruitment through local environmental variables such as sea temperature, salinity, oxygen, turbulence and advection (*137*). The persistent anomalies in the wind field associated with the NAO are responsible for alterations in the direction and strength of oceanic surface currents. Off northern Norway, this influences the north eastward flow of warmer Atlantic water and ultimately the temperature of the Barents Sea (*138*, *139*). NAO levels thus regulate the inflow of Atlantic water to the Barents Sea, influencing temperature and food availability for the larval and juvenile stages since the volume of inflow is related to the abundance of zooplankton (*136*).

Temperature is a regional hydro-climatic variable potentially affecting the survival and growth of early life stages (*140*) as well as their distribution (*141*) and recruitment (*142*). In the Barents Sea, the Kola transect temperature is representative of the Atlantic water masses in the south-central Barents Sea (*46*) and explain the dynamics for NEAC (*143*) and northward population displacement in the Barents Sea (*144*). The Kola Sea temperature (1921-2017) is an aggregated average of several depths (1-200 m) at five stations on the Kola meridian transect (33°30'E, 70°30'-72°30'N) in the Barents Sea (https://ocean.ices.dk/core/iroc) (*37*, *145*). To estimate Kola Sea temperature before 1921, we applied output from the MPI-ESM-LR of the Max-Planck-Institute for Meteorology (MPI-M) in Hamburg (https://www.wdc-climate.de/ui/) (*146*). We used the monthly surface temperature (between 70-75°N and 30-35°E) and adjusted the obtained results to the observed monthly Kola's ST measurements (1921-2005) before calculating the yearly average.

Demographic variables

We used available demographic variables that are the spawning stock biomass (SSB), number of recruits (R), Fishing mortality ($F_{5.10}$) since 1913 (*147*, *148*) as well as transient population growth (r) and generation time (μ) are extracted from (*149*).

In addition to these variables, we were able to calculate for each fish its age at maturation (in years), its growth rate (in cm/year) as well as the Fulton index K following the formula (*48*):

$K = \frac{weight}{length^3}$ (in g/cm).

S7 Sample selection

The historical samples were obtained from the otolith archive from the Institute of Marine Research (IMR; i.e., boxes with otoliths/scales from 1907, 1940, 1958, 1975 and 1999), where they have been stored since collected in envelopes at room temperature. The selection of specimens used for whole genome sequencing (from our modern data collection as well as the historical samples) was based several criteria's: i) the length distribution for the selected years, ii) sex (aiming for a 50:50 distribution) and iii) ecotype (NEAC (Northeast Arctic cod) vs. NCC (Norwegian coastal cod)) determined by the otolith readings. For a majority of the samples metadata is recorded (see **Supplementary data**) which includes length, weight, sex as well as age and age at maturation. The latter exclusively obtained for the NEAC and NCC. For the historical collection this information was obtained from the convolute information (based on original readings), whereas for the modern samples the recordings were based on two independent readings, where the second reading was conducted with the goal of including age at maturation. The otolith readings were conducted by technicians at IMR (*101*). The individuals selected for sequencing were based on the length distribution for the sample collections for the respective years (see **fig. S14** for more details).

S8 Sample preparation

The extractions were made in chronological order starting with the oldest material (N_{1940}=33, N_{1958}= 24, N_{1975} = 24, N_{1999} = 23), with one negative control per extraction. The subsequent library preparations were conducted according to Meyer and Kircher protocol (*150*), using indices exclusively used for historical samples and thus, ending up with N_{1940}=23, N_{1958}=23, N_{1975}= 23, N_{1999}=22. The quality of libraries was checked with a High Sensitivity DNA Assay Kit (Agilent Technologies) in an Agilent 2100 Bioanalyser. Quality was based upon concentration and length of fragments. For samples with low concentration but prominent peak(s), re-amplifications were made running additional 4-6 cycles of PCR (sample names marked with R or RA). For 1975 and 1999 respectively, 4 and 8 libraries were excluded based upon quality readings. Excluded libraries showed no peaks, indicating failed extractions. The sequencing conducted, were run stepwise, first performing test sequencing (N_{1975} = 19, N_{1999} = 14, 2 lanes for both years) and then production sequencing (N_{1975} = 17, N_{1999} =11, 8 lanes (1975) and 6 lanes (1999) respectively. Libraries were excluded from production pools based on low read number, clonality (N_{1975}=2, N_{1999}=2) or being fully sequenced (N_{1999}=1). Sample preparation for 1907 is described in Pinsky *et al.* (*6*).

S9 Data processing

The BWA (Burrows-Wheeler Aligner) pipeline of Paleomix was used for Adapter removal (AdapterRemoval v. 2.7.1) (*151*) as well as for the mapping to the reference genome gadMor2 (*115*, *152*). BWA-backtrack (BWA v.0.5.9-r26-dev/0.5.10) (*153*) was used for the historical samples while BWA-mem (*154*) was used for the modern samples. Subsequently, filtering of e.g., unmapped reads and reads of low quality (25<Q), as well as removal of PCR duplicates, indel-realignment in GATK (Genome Analysis Toolkit v.3.6) (*155*) were performed. aDNA damage assessment was made with mapDamage (v.2.0 and 2.2) (*156*). Base frequencies and

misincorporation patterns were assessed at 5' and 3' end for all historical NEAC from 1940-1999 (see **fig. S15A**), using mapDamage and visualisation in R. Deamination for 1907 already assessed in a previous study by Pinsky *et al.* (*6*). For all years (including 1907) deamination was determined to be under 0.05 (see **fig. S15B**). The above-mentioned data processing was conducted following the Paleomix (v. 1.2.5) pipeline (*157*). Soft-clipped reads were removed from both modern and historical data (nuclear genome). This was done due to the presence of multiple SNPs being called on those reads after one other. Based on assessment of the average coverage (both before and after removal of soft clipped reads) using Samtools (*158*) (v.1.3.1), two of the modern samples with higher coverage were down-sampled, to approx. 7x coverage.

S10 Read length assessment

Read lengths were obtained from bam-files using Samtools (*158*) v.0.1.19 (view ${1}.bam | cut -f 10 | perl -ne 'chomp;print length($_) . "\n"' | sort | uniq -c > Read_length_summary${1}.txt). The bam-files for modern populations were sub sampled to roughly 5x coverage prior to obtaining the read length summaries with Samtools v.0.1.19 (view -bs 42.6 ${1}.bam > ${1}.5X.bam), to speed up computation. None of the bam files had soft clipped reads removed prior to retrieving read length summaries (this was later applied in filtering), and none of the 5x sub sampled files were later used in variant calling. Read length distributions were assessed visually using R v. 4.1.1 (see **fig. S16**) and by calculating the mean read length per population/year (also in R). The latter were calculated by first calculating the mean read length per individual and then the mean of the population means. The read length per individual was calculated by multiplying the number of reads of each length with the read length, then taking the summary of the products and dividing with the total number of reads. The modern samples have more or less uniform distributions with the majority of reads at either 100bp or 125bp (**fig. S16**). The historical samples have mean read lengths per population between roughly 56 and 88 bp (**fig. S16**). The historical catch years with longest mean read length (RL) were 1999 (RL = 88.3 bp) and 1907 (RL = 86.5 bp), followed by 1975 (RL = 76.7 bp). Shortest mean read length were found in 1958 (RL =56.1 bp) and 1940 (RL =58.6 bp). Two different datasets, *reduced* and *full* were setup, the *reduced dataset* excluding the samples from the two years with shorter read length 1940 and 1958, and the full dataset.

S11 mt contamination and variant calling

Some of the samples were also excluded prior to variant calling based on contamination assessment of the mt (mitochondrial) data. This assessment was conducted with calculation of % heterozygous sites based on diploid variant calling of the reads mapping towards the mitochondrial genome. For the *reduced dataset* (excluding 1940 and 1958) three individuals were excluded, whereas in the *full dataset* (including 1940 and 1958) seven individuals were excluded. The excluded samples exceeded a threshold of 3% of heterozygous sites (**fig. S17A**), when using 1461 mt sites obtained from the variant calling conducted on the *full dataset*. These samples also showed a suspicious (banding) heterozygosity profile across the mtDNA in IGV (integrative genomics viewer) (*109*) (**fig. S17B**). However, we did allow some heterozygosity due to the known presence of heteroplasmy in the mtDNA of Atlantic cod (*159*). The variant index type was set as linear, the index parameter set at 128000.

S12 Filtering

Filtering was started (Step1) by converting gzvcf files to bcf, and applying the filter recommendations for SNPs from GATK when unable to run VSQR calibration in BCFtools (*103*)

(v.1.1), and setting --SnpGap to 10, removing SNPs within close range of each other, to avoid false SNPs. Furthermore, all indels were removed (Step2), max mean depth set as 20 and only bi-allelic sites kept using VCFtools (v.0.1.14) (*104*). That only bi-allelic sites were kept is due to the fact that sample sizes are small and data is low in coverage and thus our ability to look at multiallelic sites is compromised. Sites at the individual level with DP < 3 were set as missing in VCFtools (v.0.1.14), and individuals with more than 60% data missing were excluded in the dataset without 1940 and 1958 ($N_{>60\%}$=7), and in the dataset with 1940 and 1958 ($N_{>60\%}$=13), leaving 192 individuals and 228 individuals respectively. The 60% limit was a reached compromise between losing the individuals most affected by missingness while keeping moderate sample size. Next filtering on missingness per site, allowing a maximum of 20% missingness reduced the missingness per individual, setting max-missing count to 38 in the dataset without 1940 and 1958, yielding 10 042 648 sites, and max-missing count to 45 in the dataset with 1940 and 1958 and 7 002 845 sites. Furthermore, filtering was made on minimum allele frequency, setting the limit to 0.03, yielding 1 790 257 sites in the dataset without 1940 and 1958 and 946 084 sites in the dataset with. The 0.03 minimum allele frequency was a reached compromise between bias including sites with low frequency and losing sites. Two alternative datasets were made excluding the minimum allele frequency filtering entirely for the purpose of looking at nucleotide diversity, Tajima's D (*49*), iHS (*50*) (the integrated haplotype score) and extended haplotypes (*53*). The filtering steps described below also apply to those two datasets, but numbers of sites deviate and aren't reported below. The datasets were assessed for excess heterozygosity (HWE) in VCFtools (v.0.1.14) and sites with a *P* value under 0.001 excluded. For the dataset without 1940 and 1958, 99% or 1 766 804 sites were kept, for the dataset with 1940 and 1958, 99% or 935 079 sites were kept. Correction based upon mappability was made using excluding regions defined with GEM Mappability (*105*) (25kmer, 150bp from edge), keeping 451 660 regions in the gadMor2 genome. This left 566 930 sites in the dataset without 1940 and 1958 whereas 326 931 sites were left in the dataset with 1940 and 1958. Known repetitive regions in gadMor2 were excluded, yielding 539 061 sites in the dataset with 1940 and 1958 and 310 171 sites in the dataset without 1940 and 1958. Removing repetitive sites and regions of low mappability is especially important when dealing with short read length data (*105*, *106*)

Sites showing deamination (C>T and G>A) were removed by a combination of cat and awk (| awk '($4!="C" && $5 != "T") {print $0}'| awk '($4!="G" && $5 != "A") {print $0}'), yielding 188 498 sites in the dataset without 1940 and 1958 and 111 120 sites in the dataset with.

Sites were tested looking at the allele contribution on heterozygote calls (*6*). This was done by extracting allele and genotype data in VCFtools (v. 0.1.14). A binominal test was then used to see if SNPs fall within accepted 0.5 frequency, and SNPs with a *P* value under 0.05 removed. For the dataset without 1940 and 1958, 166 941 sites passed the binominal test (89% of sites), in the dataset with 1940 and 1958, 82 365 sites (74%) passed.

The unplaced scaffolds present in the gadMor2 was removed at last step of filtering (scaffolds not placed within LG01-LG23), leaving 166 484 sites in the dataset without 1940 and 1958, and 82 162 sites in the dataset with them.

Missingness per individual and site were checked with VCFtools (v.0.1.14 or v.0.1.16), after the filtering. Analysis of the dataset without 1940 and 1958 revealed no individuals left with missingness exceeding 50%, and only two individuals exceeding 45% and missingness per site did not exceed 10% for any site. 72% of sites (119 924) showed missingness exceeding 5%, which was deemed acceptable.

For the dataset with 1940 and 1958 no individuals had missingness exceeding 45% and only two individuals had missingness exceeding 40%. Missingness per site did not exceed 10% for any site, however 90% of (74 054) showed missingness exceeding 5%.

S13 LD decay

Linkage disequilibrium (LD) was determined per population/year for LG01-LG23. r^2 was calculated in plink (1.90b5.2) (*160*). Settings were r2 --ld-window 1000 --ld-window-kb 500 --ld-window-r2 0). LD decay plots (**fig. S18A-C**) were generated using binning and averaging the distance within each bin (*161*, *162*). Due to the difference in r^2 between the populations/years we tested the effect of sample size on the r^2, sub-setting all populations to 11 (--max-indv 11) (**fig. S18A-C**).

S14 Inversion breakpoint regions

Inversion borders for exclusion and analysis restricted to inversions were adjusted by analysing the pairwise F_{ST} (*51*) comparisons of the NEAC (modern and historical) with NCC (both NCC 2011 and 2014), as well as comparisons with the outgroups (NOR 2002, BOR 2012, and GOM 2009) (see **fig. S19** and **fig. S20**). Initial borders (*29*), after set to positions in gadMor2 (see **table S28**), were found to be imprecise for the present dataset. Initial borders (*29*) were therefore compared with borders obtained from other historical cod (*163*) (see dotted and dashed lines in **fig. S19** and **fig. S20**). Graphically alternative hard cut-offs were found, and from those confidences were created and used as borders (see **table S29**). All comparisons were plotted but only 4 F_{ST} comparisons are shown (see **fig. S19** and **fig. S20**), the rest of the comparisons are available upon request.

S15 Inversion scoring

Inversion scoring was made in the Integrative Genome Viewer (IGV) (*109*) for individuals with high amount of missing data (>40%), visualized with Genotype Plot (*108*) as explained in the main document. Visualization might not always reflect the view in IGV (especially for individuals with high missingness), due to removal of sites with >50% missingness as well as removal of invariant sites and analysis with Genotype plot required VCF files to be split per year due to computation (except when looking at genes), this meant that for some years in visualization not all genotypes were present. As such original IGV inspection score and check over genes were trusted over banding patterns seen in Genotype Plot.

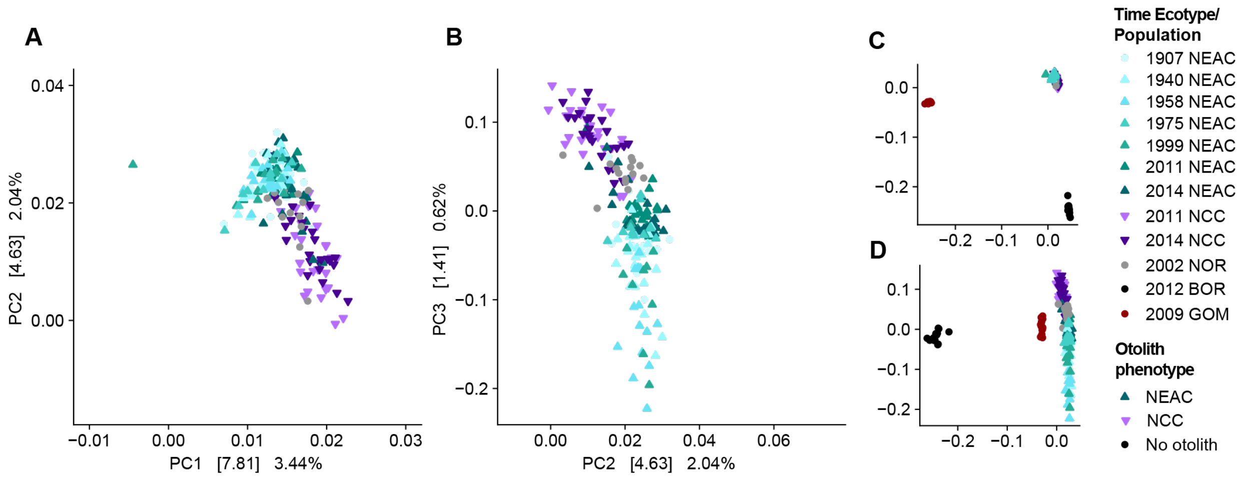

Fig. S1. PCA plots for all significant eigenvectors for *full* WGS dataset. Zoom in (**A**) for the first two principal components, zoom in (**B**) for the second and third principal component. Eigen vector mean within square brackets for full PCA as well as percentage explained by eigenvector. Full PCA for the first two principal components (**C**) and full PCA for the second and third principal components (**D**).

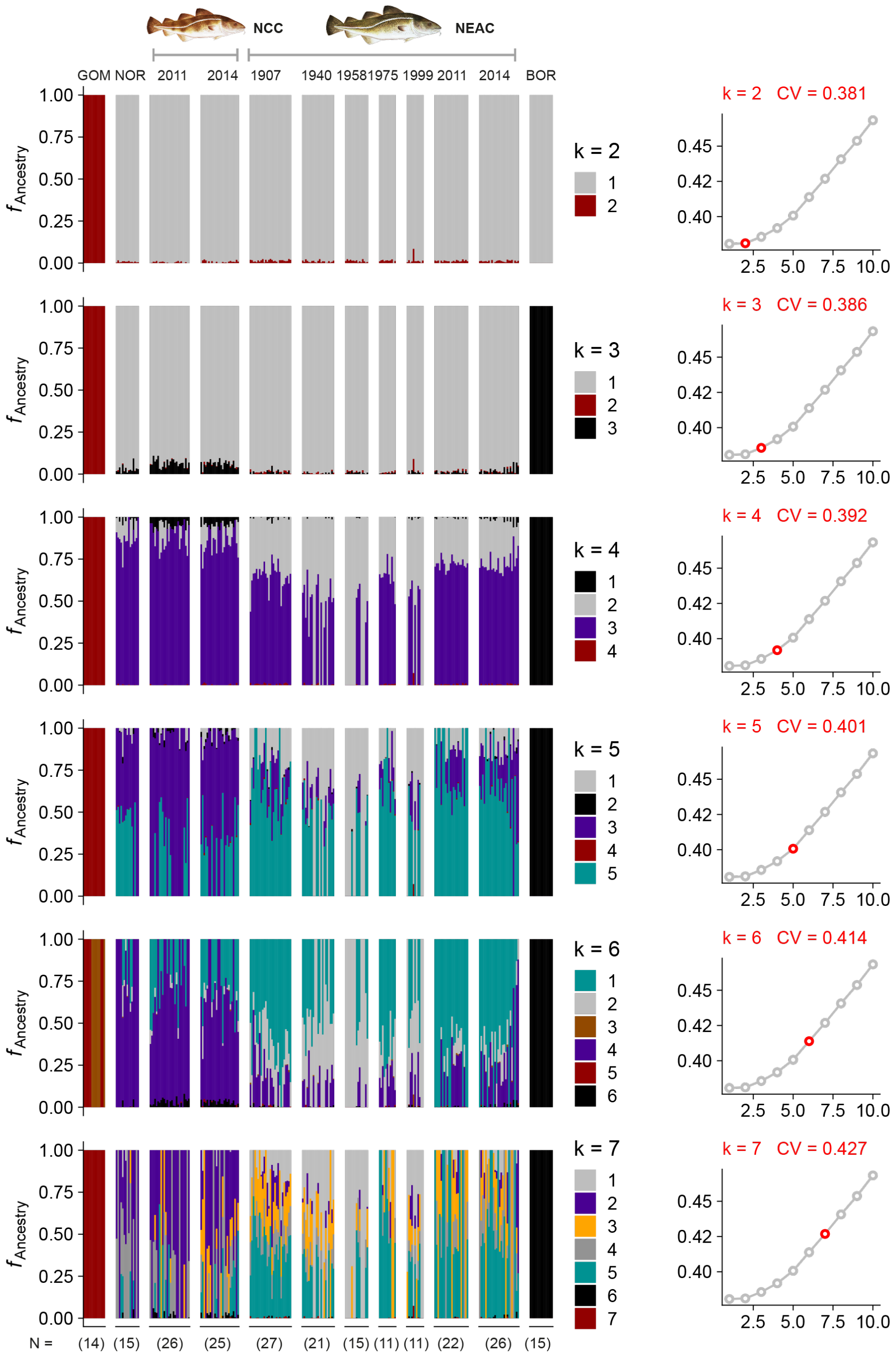

Fig. S2. Admixture plot for *full* WGS dataset split per sampling year and population and CV. Each bar is one sample. N denotes the number of samples within each division and is given within brackets. k shows the number of populations. CV plots shown on right side, k used marked with red. Fish illustrations by Cecilia Helmerson (UiO).

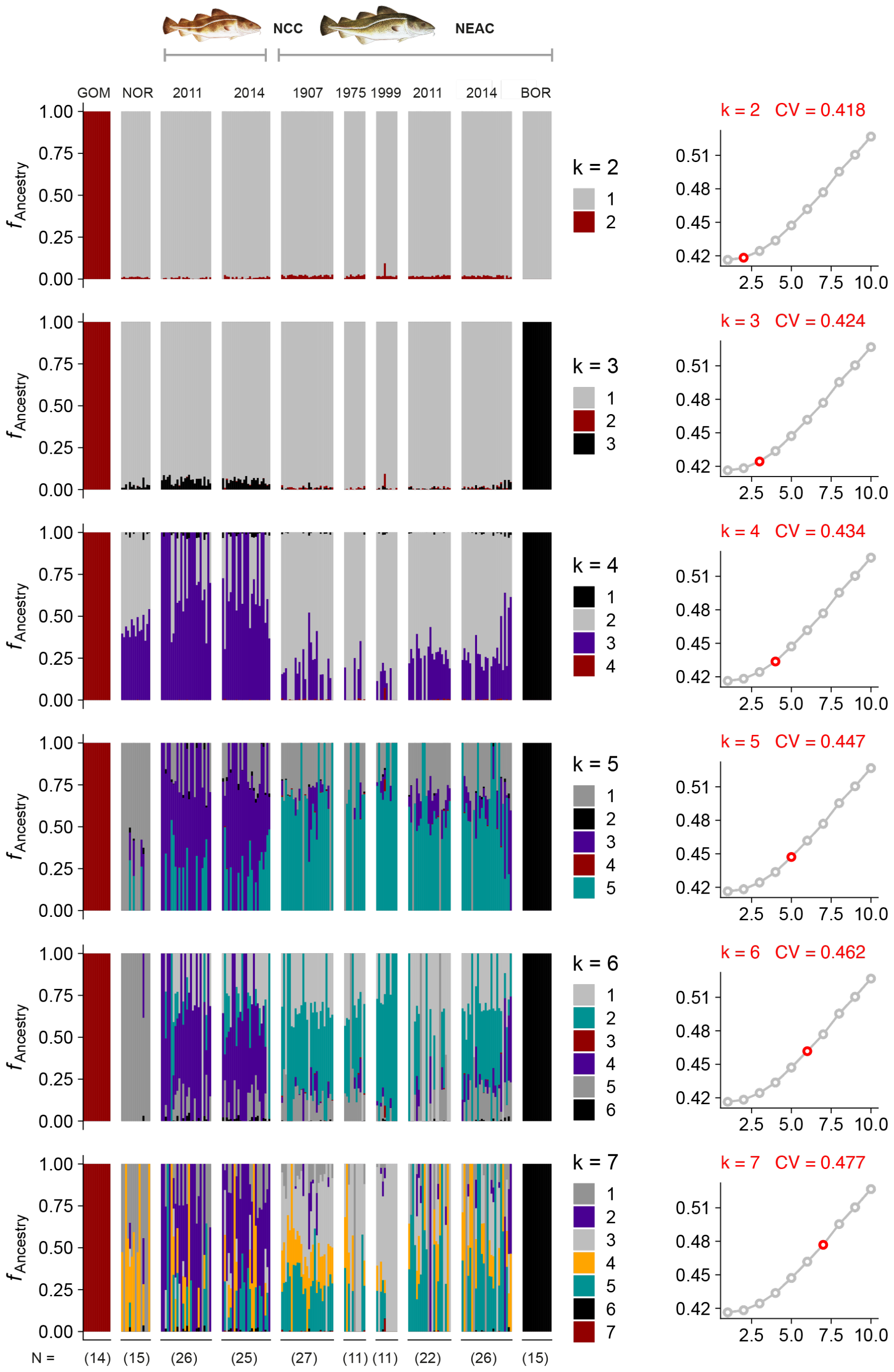

Fig. S3. Admixture plot for *reduced* WGS dataset split per sampling year and population and CV. Each bar is one sample. N denotes the number of samples within each division and is given within brackets. k shows the number of populations. CV plots shown on right side, k used marked with red. Fish illustrations by Cecilia Helmerson (UiO).

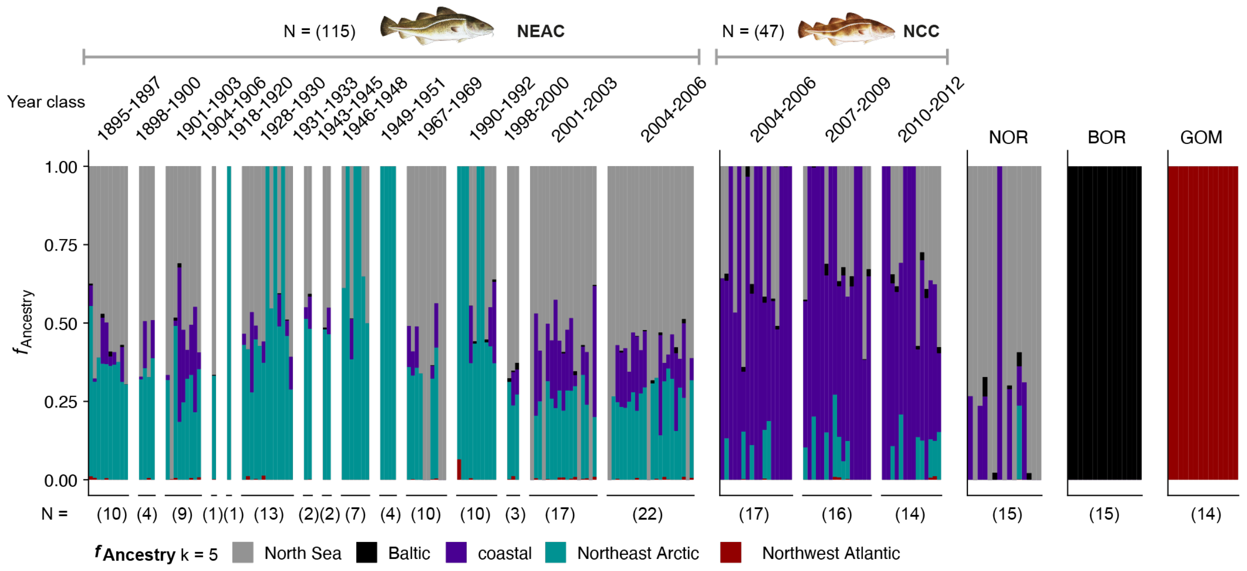

Fig. S4. Admixture plot for *full* WGS dataset split per year classes for NEAC and NCC at k = 5. Individuals with > 30% missing data removed. Fish illustrations by Cecilia Helmerson (UiO).

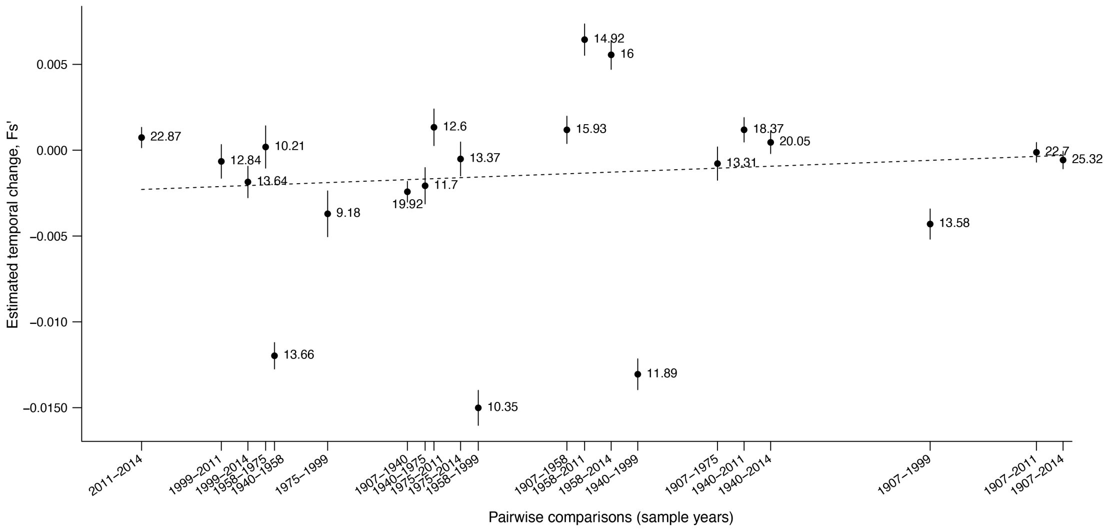

Fig. S5. Estimates of temporal allele frequency shifts, F_s'. F_s'. (black dots), between pairs of samples with 95% CI (vertical bars). Sample intervals (x-axis) are arranged after number of years between samples. Numbers to the right of point estimates represent harmonic mean sample sizes. The horizontal, dotted line represents linear regression of F_s' against number of years between samples in pairwise comparisons (nonsignificant slope = 1.9 $*10^{-5}$, *P* value=0.588).

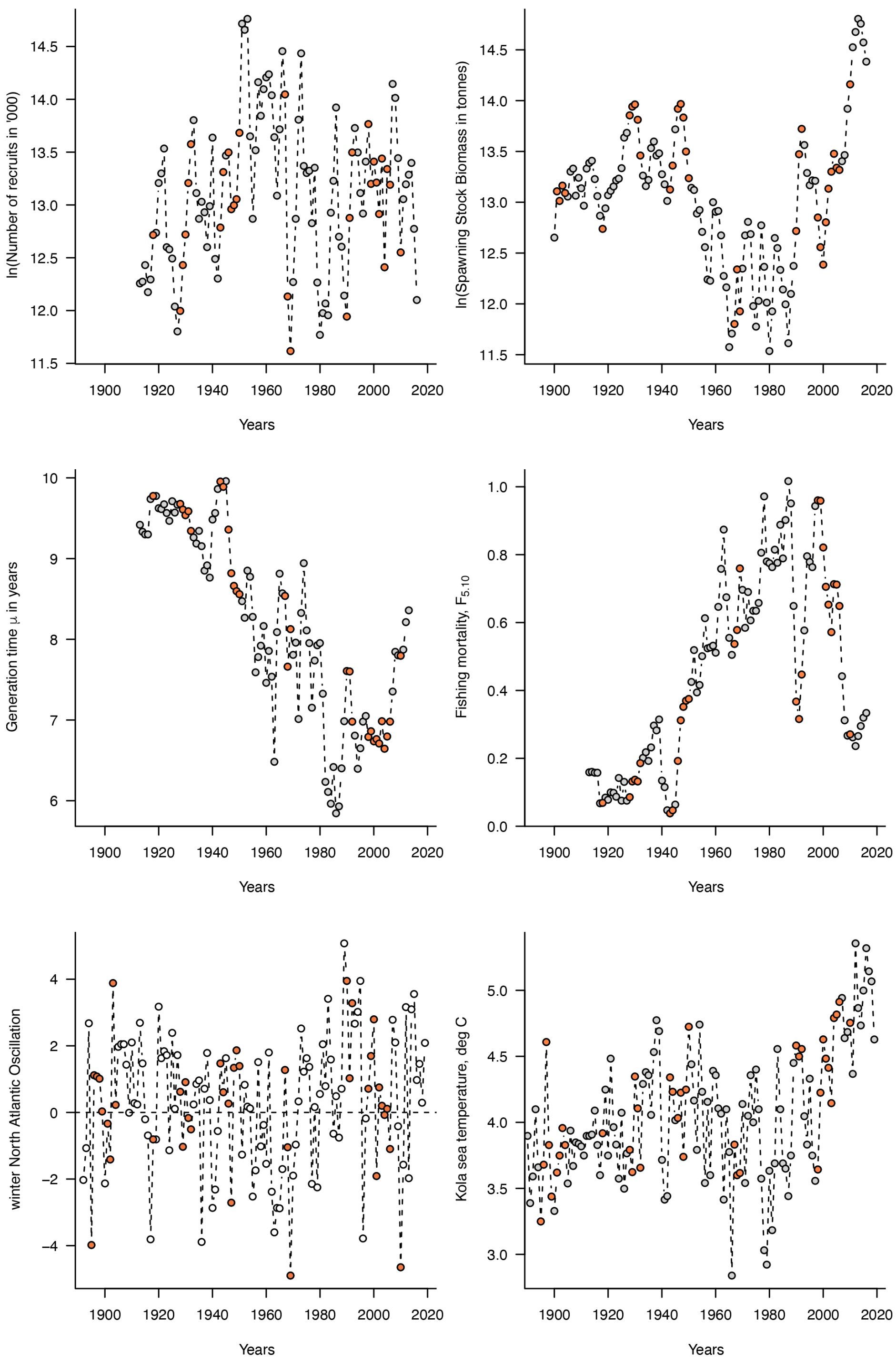

Fig. S6. Demographic and environmental variables used as potential explanatory variables. For all plots, the bold red dots correspond to the years where sampled cod were born and when we have genomic information.

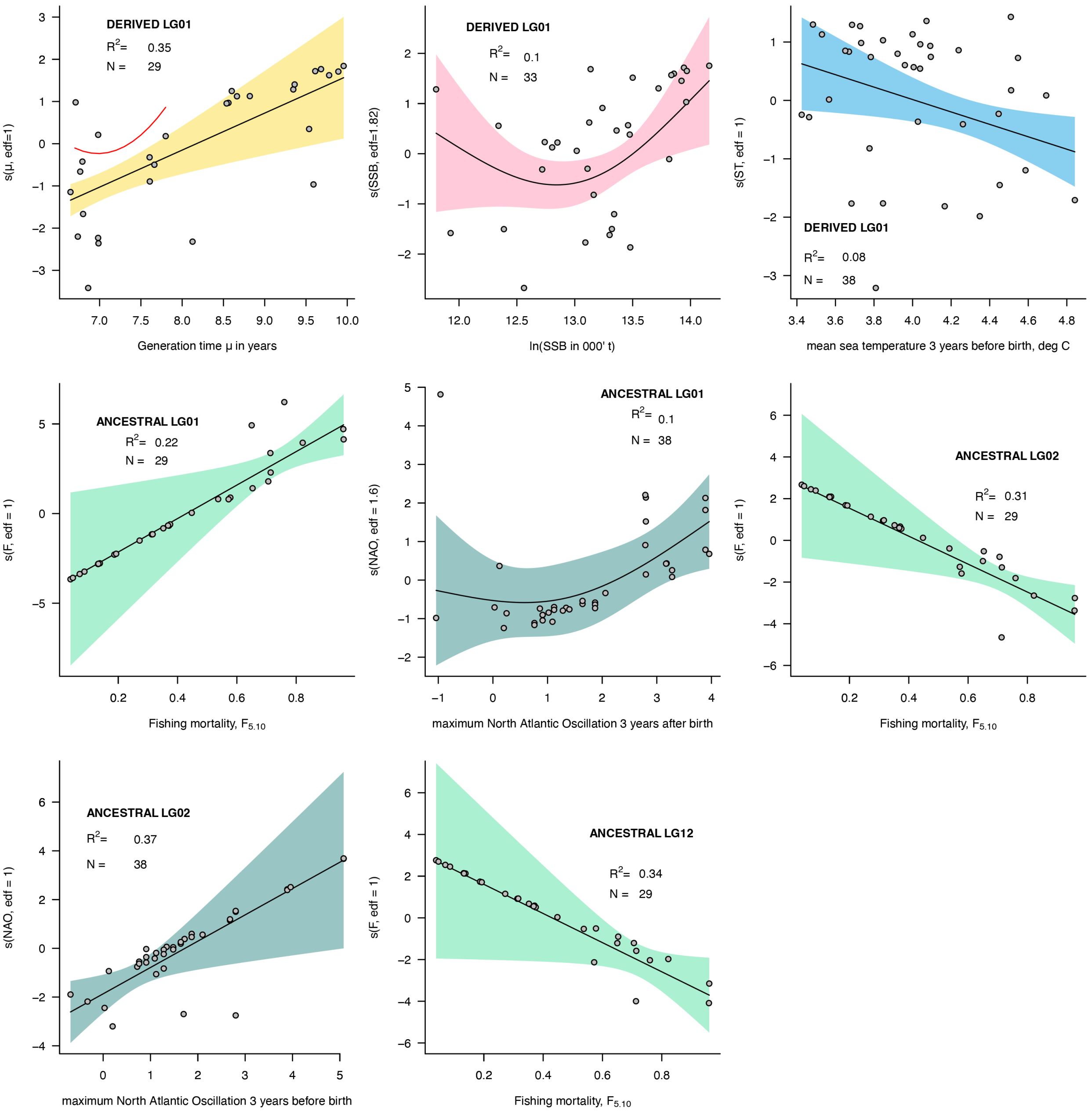

Fig. S7. GAM predictions partial plots for different inversions where significant relationships were found. For each plot, the x-axes show the covariate and the y-axes the partial effect that each covariate has on the response variable. s(X, y) is the smoothing term, where X represents the explanatory variable and y is the estimated degrees of freedom (edf) of the smoothing term. Black line shows the smooth term effect of the considered covariate on the proportion of the inversion considered with the pointwise 95% confidence interval around the mean prediction (shaded area). The dots indicate the residuals.

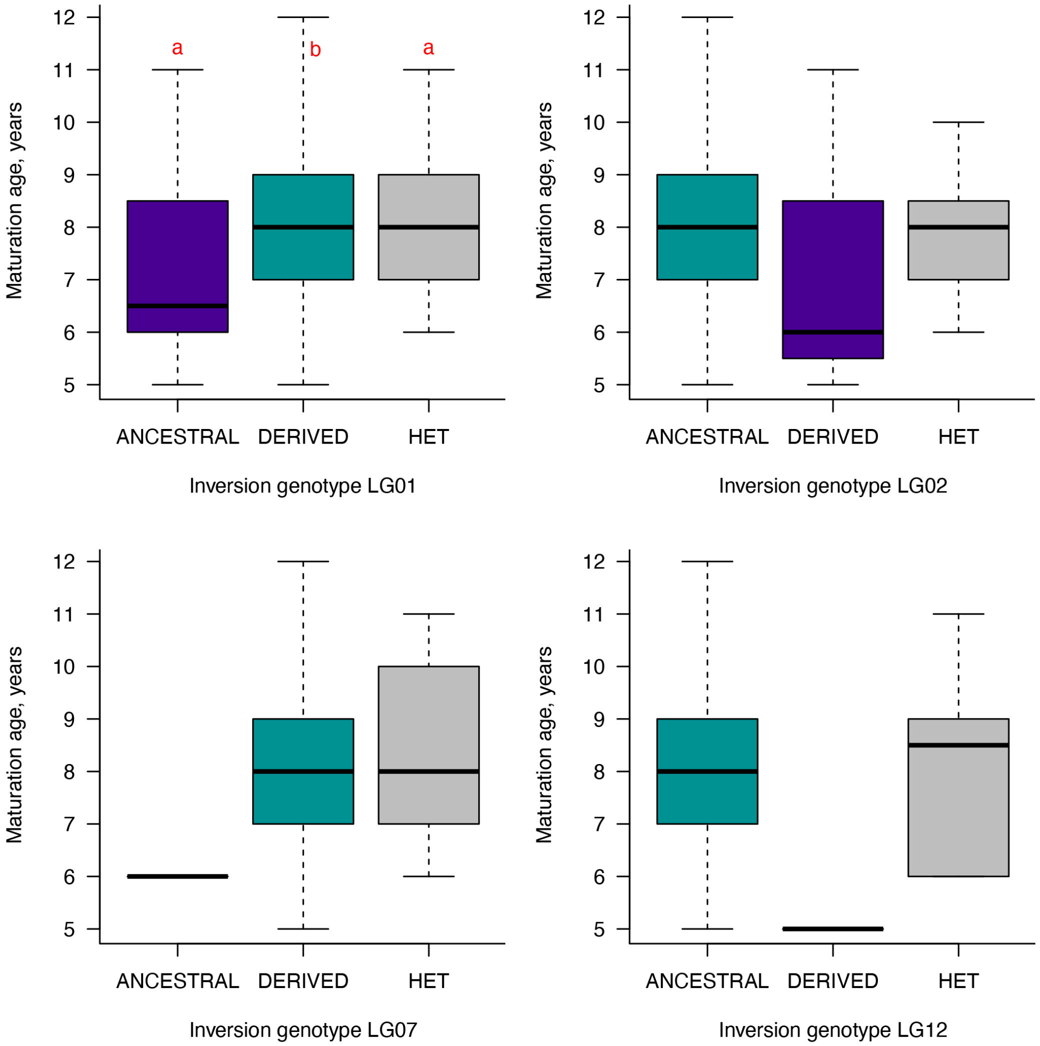

Fig. S8. Description of age at maturation age for the three different inversion genotypes (ANCESTRAL, HET and DERIVED) on LG01, LG02, LG07 and LG12. The horizontal line is the median value, the box delimits the 1st and 3rd quartiles (interquartile range (IQR) criterion), while the whiskers (error bars) show the 5th and 95th percentiles. Level of differences (here $P<0.1$) between categories obtained with Kruskal-Wallis rank test followed by a Dunn test are indicated by letters. Note that the data contain many ties and categories are of different length making the statistical test less reliable. HET= heterozygous.

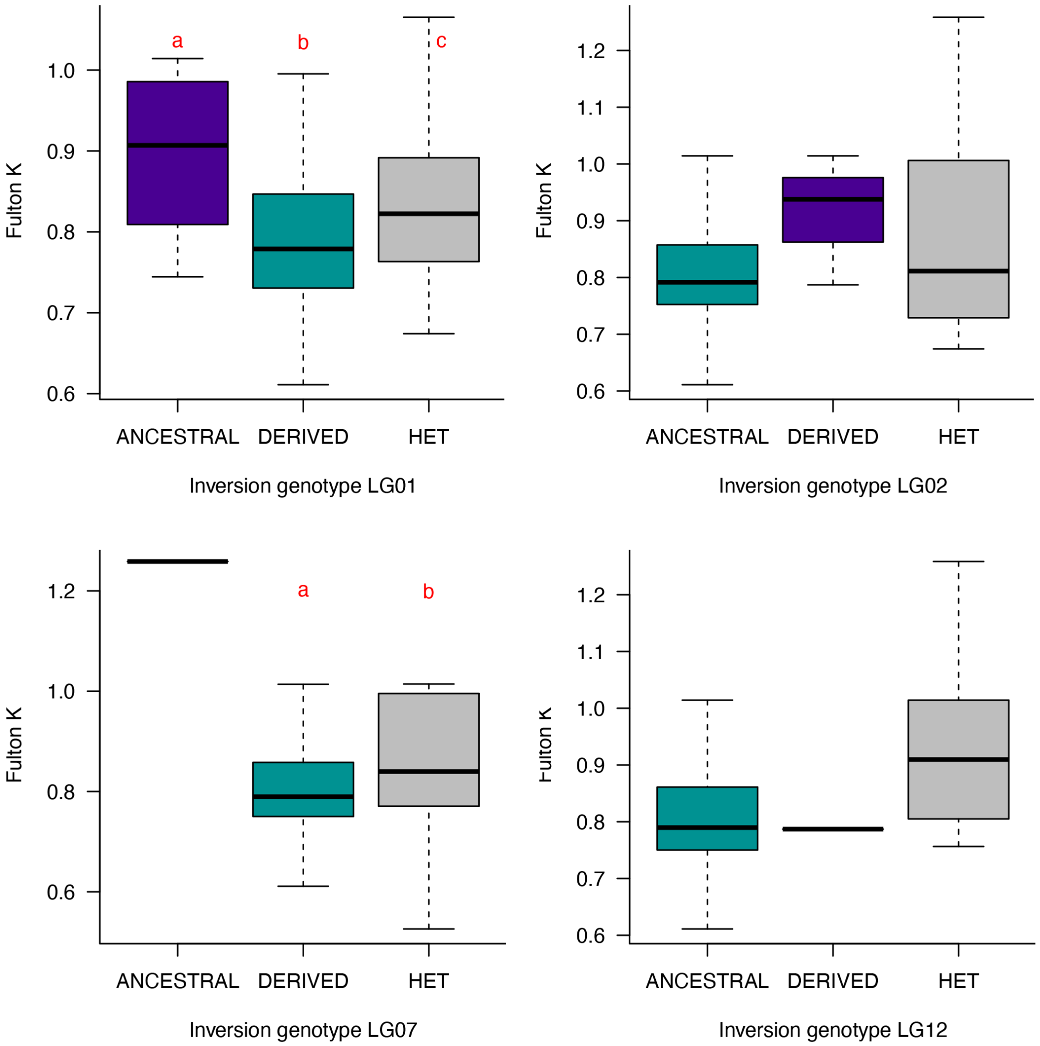

Fig. S9. Comparison of the Fulton K index for the three different genotypes on LG01, LG02, LG07 and LG12. The horizontal line is the median value, the box delimits the 1st and 3rd quartiles (interquartile range (IQR) criterion), while the whiskers (error bars) show the 5th and 95th percentiles. Level of differences (here P<0.05) between categories obtained with Kruskal-Wallis rank test followed by a Dunn test are indicated by letters. Note that the data contain many ties and categories are of different length making the statistical test less reliable. HET= heterozygous.

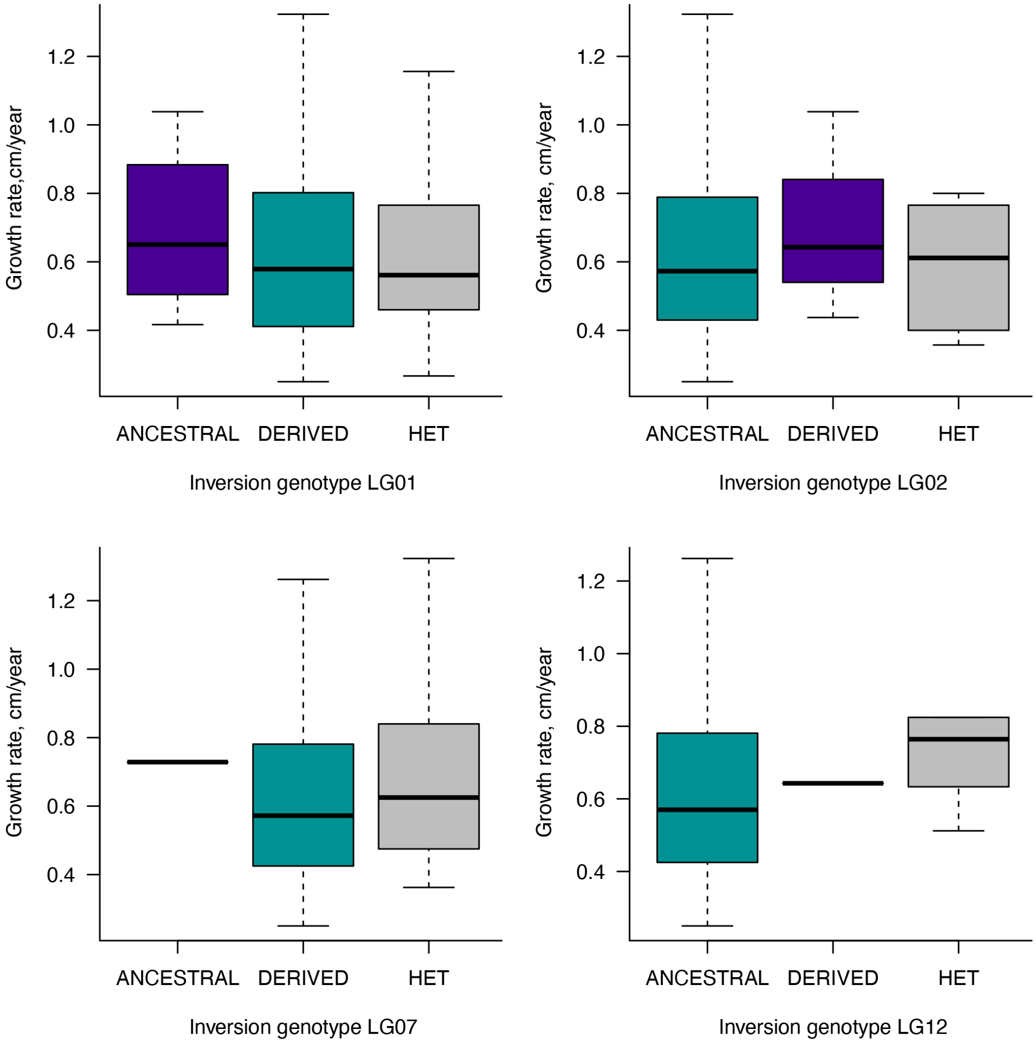

Fig. S10. Comparison of the growth rate for the three different genotypes on LG01, LG02, LG07 and LG12. The horizontal line is the median value, the box delimits the 1st and 3rd quartiles (interquartile range (IQR) criterion), while the whiskers (error bars) show the 5th and 95th percentiles. HET= heterozygous.

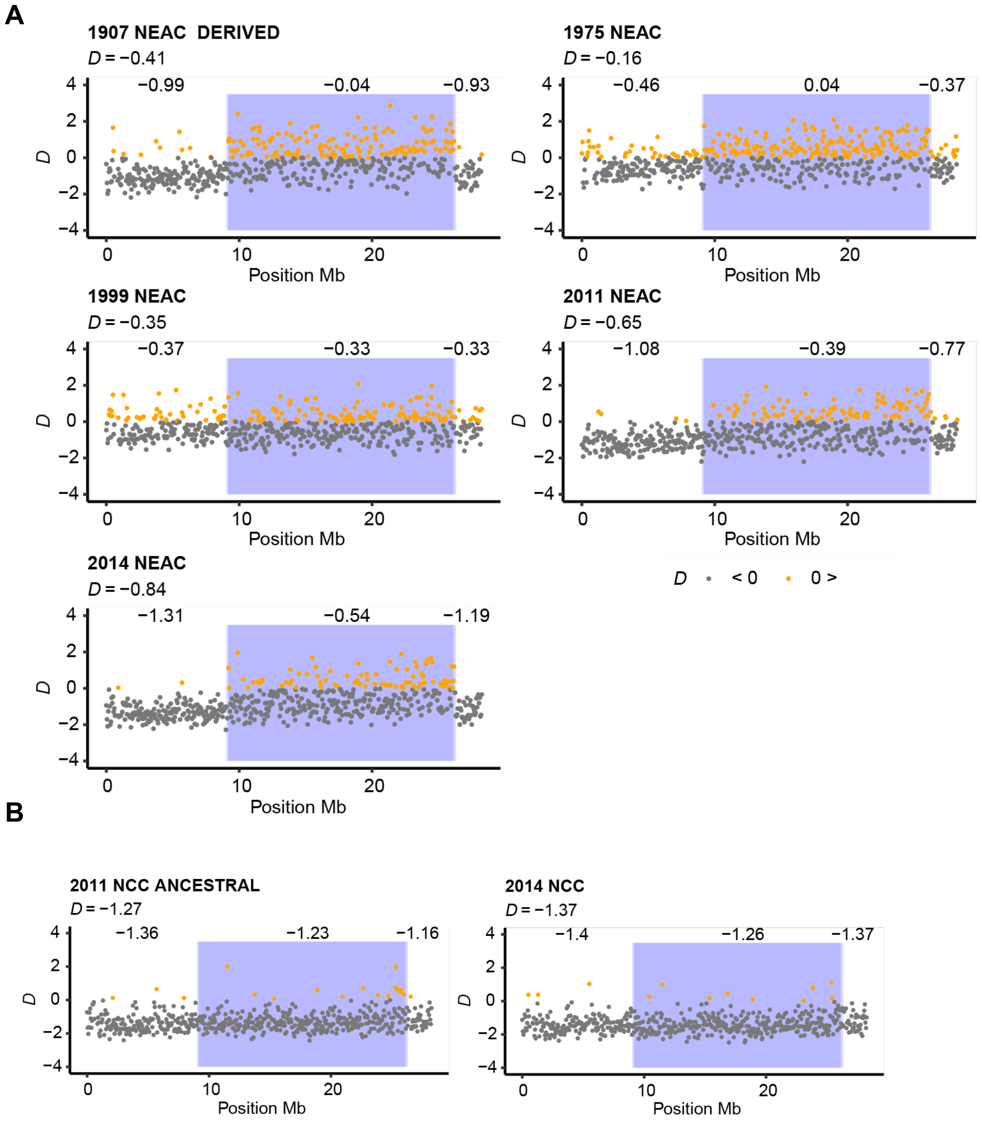

Fig. S11. Selection pressure in the form of Tajima's D on inversion at LG01 for NEAC and NCC for homozygous individuals. (**A**) Tajima's D for homozygous DERIVED NEAC split per catch year. (**B**) Tajima's D for homozygous ANCESTRAL NCC split per catch year.

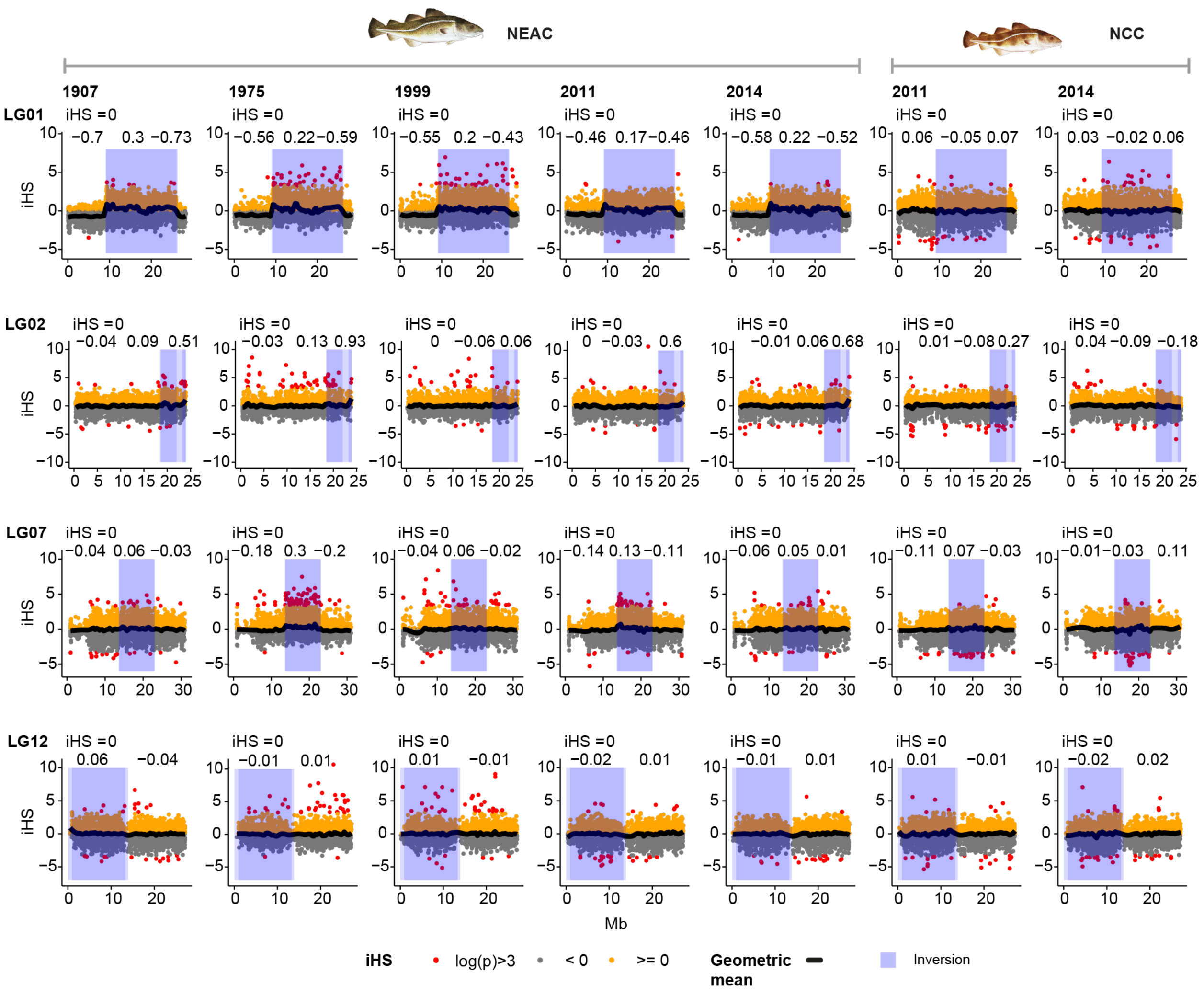

Fig. S12. Signatures of selection over the four inversion regions for NEAC over the past century and NCC in modern times. iHS (integrated haplotype score) (using the *reduced dataset)* along the chromosomes over the inversion regions on LG01 LG02, LG07 and LG12 for NEAC and NCC for the different years. iHS over or equal to 0 coloured in orange and negative coloured in grey. Statistically significant coloured in red. iHS for entire chromosome given on top of each plot and averages inside and outside of inversion. Geometric mean shown with black line. Inversion regions marked in blue. Fish illustrations by Cecilia Helmerson (UiO).

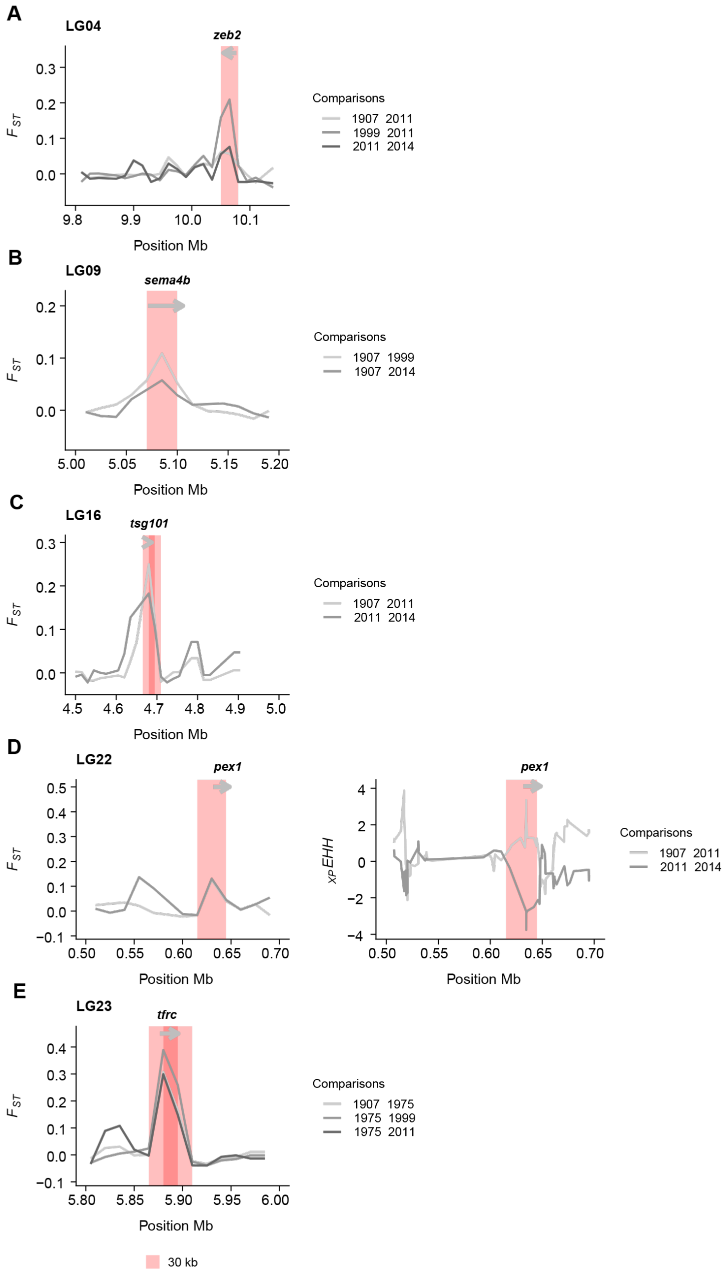

Fig. S13 Allele frequency shifts and outlier candidate genes for NEAC over the past century. (**A-E**) Significant outliers in more than two pairwise comparisons between years, detected by F_{ST}, AFD and/or $_{XP}$EHH analyses, with significant values for F_{ST} with a q-value ≤ 0.05, the AFD quantile = 99.9, and the $_{XP}$EHH log(P) ≥ 3 are presented with F_{ST} plots and $_{XP}$EHH plots (when applicable). (**A**) On LG04 the outlier identified is positioned inside an intron of the *zeb2*: zinc finger E-box-binding homeobox 2 gene. (**B**) On LG09 the outlier is positioned within the coding region of *sema4b*: semnaphorin-4B, and responsible for an amino acid change. (**C**) On LG16 the outlier is positioned inside an intron of *tsg101*: tumour susceptibility 101 gene. (**D**) On LG22 the outlier is positioned inside an intron of *pex1*: peroxisome biogenesis factor 1 (**E**) On LG23 the outlier is positioned inside an intron of *tfrc*: transferrin receptor 1.

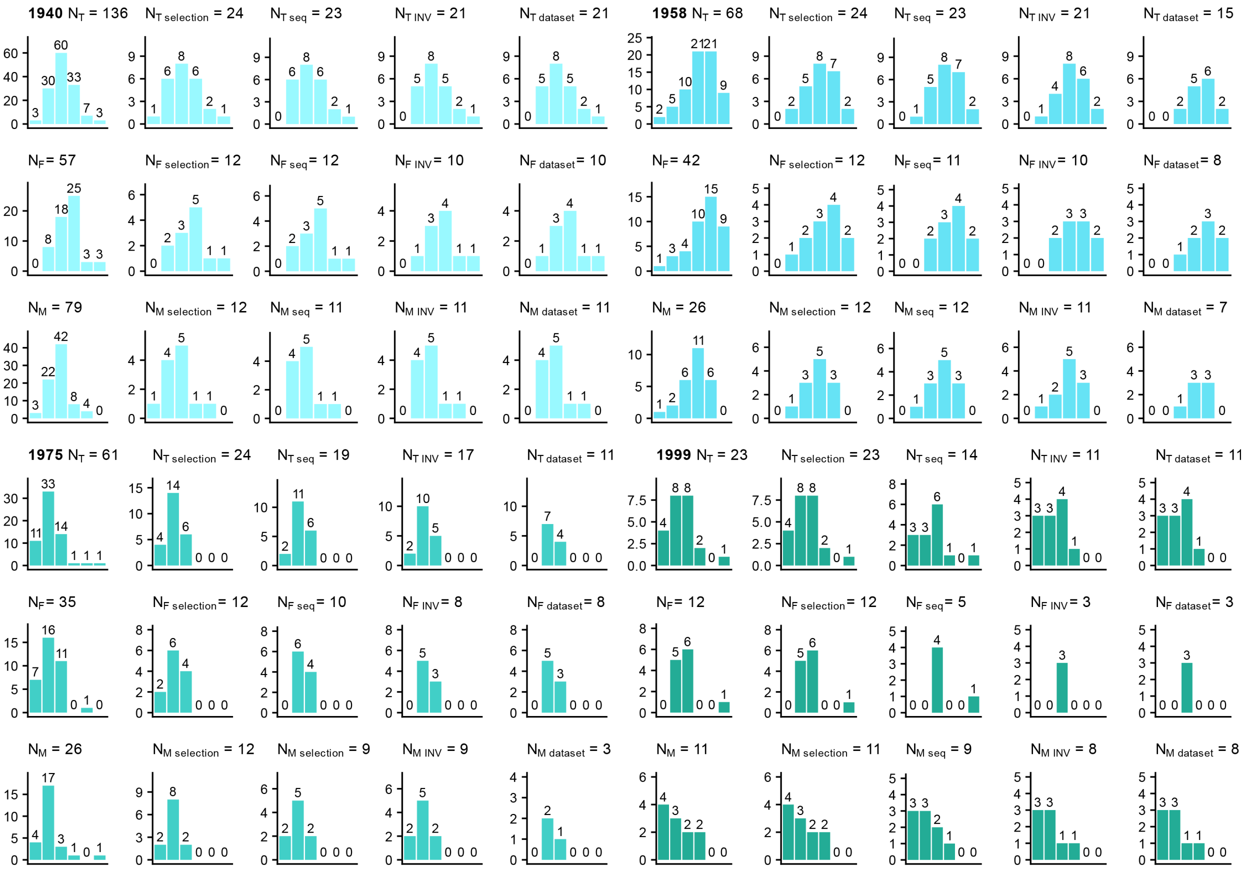

Fig. S14. **Sample selection plots for 1940 - 1999 NEAC**. Graphs based on length of individuals landed in Lofoten in 1940, 1958, 1975 and 1999. Plots showing number of individuals (N) within length categories, divided top to bottom: total (N_T), female (N_F) and male (N_M), and left to right: total in library prep ($N_{T\ lib}$), sequenced (seq), scored for inversions (INV) and individuals present in final *full* WGS dataset. Size bins are set as 65 cm (60 -70 cm), 75 cm (71-80 cm), 85 cm (81-90 cm), 95 cm (91-100 cm), 105 cm (101-110 cm) and individuals with a length equal to or exceeding 111cm.

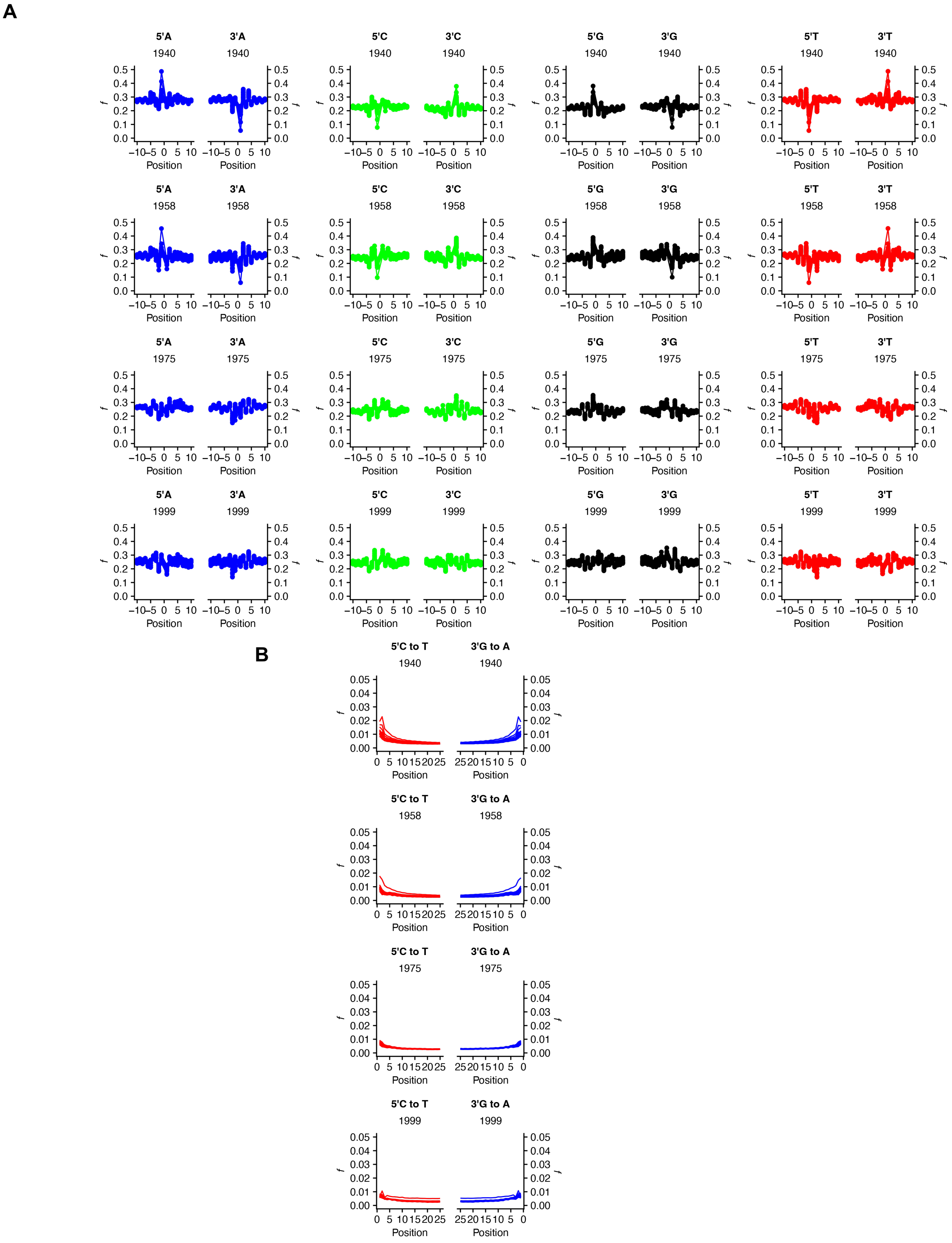

Fig. S15. mapDamage base frequencies and deamination plots. (**A**) Base frequencies split per population/year for adenine (A), cytosine (C), guanine (G) and thymine (T) at 5' and 3' end. (**B**) Deamination plots showing frequency of cytosine to thymine (C>T) at 5' end and guanine to adenine (G>A) at 3' end.

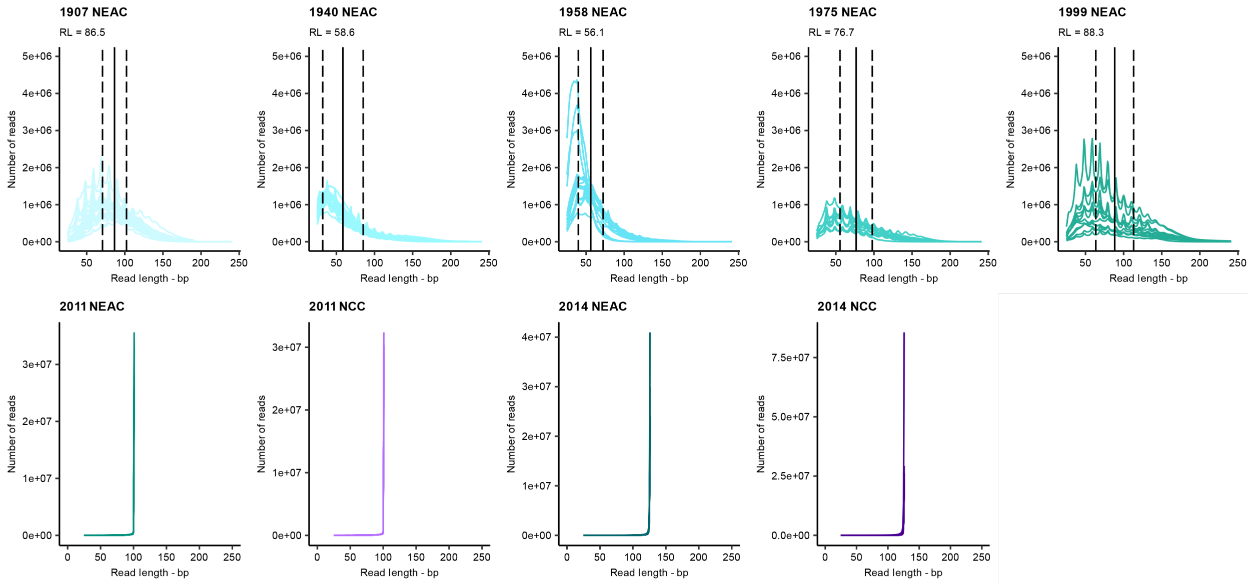

Fig. S16. Read length distributions for the historical and modern NEAC as well as modern NCC. Distributions are split by sampling year, left to right chronologically. Solid line showing RL (mean read length) per year, whereas dashed lines showing two standard deviations from the RL. RL value displayed above the mean line.

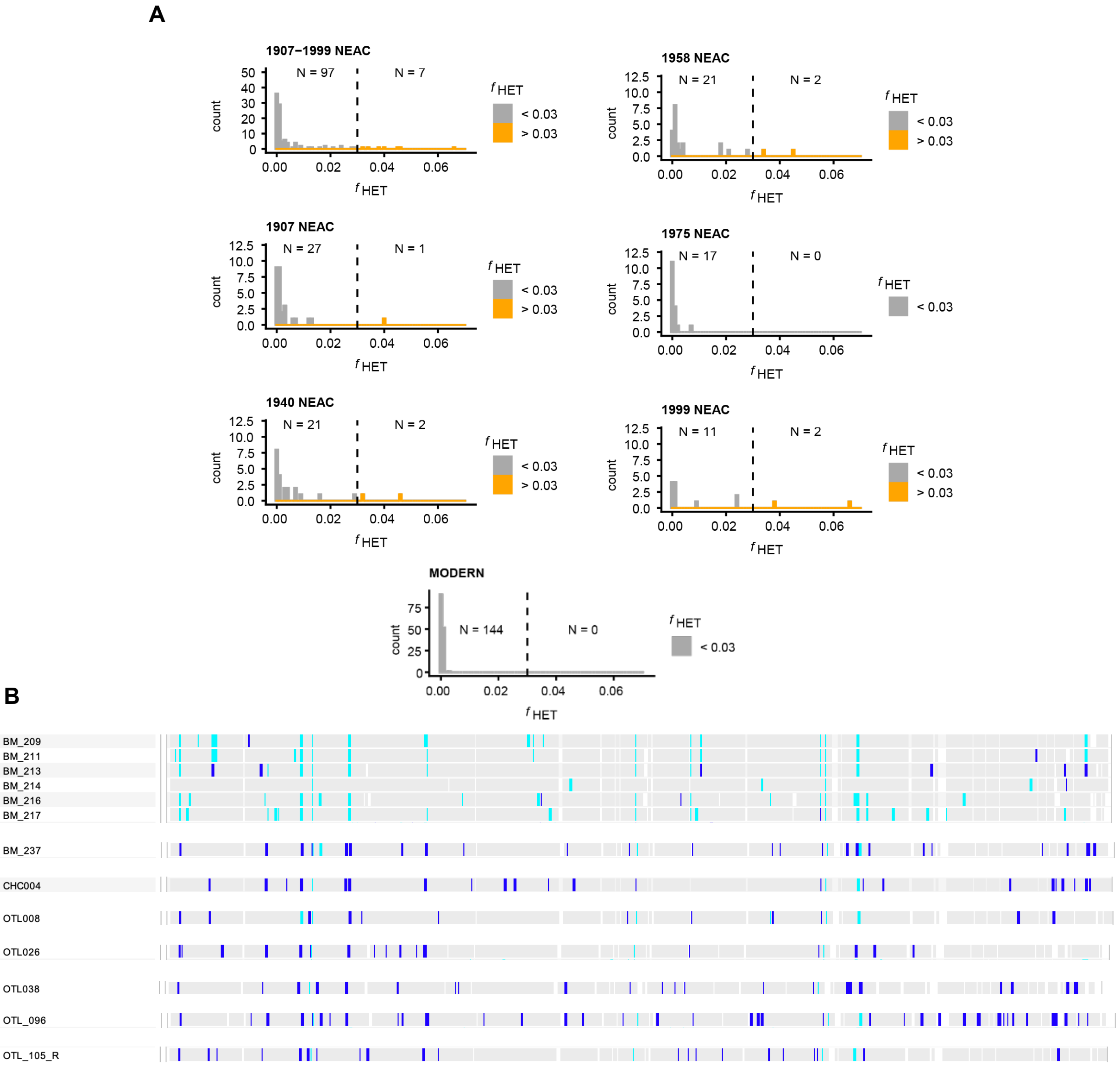

Fig. S17. Contamination plots. (**A**) Plots showing distribution of mitochondrial heterozygosity, fraction of heterozygous sites in mitochondrial DNA (f_{HET}) for historical NEAC samples and all modern samples. Historical samples shown pooled and split per year. (**B**) Screen dumps from IGV of VCF file with ploidy set as 2, total 16kb of mt genome, first showing a few individuals without suspicious banding pattern, then 7 individuals with suspicious heterozygosity pattern (heterozygosity >3%). Heterozygous sites dark blue, homozygous as in reference genome in grey and alternative homozygous in turquoise.

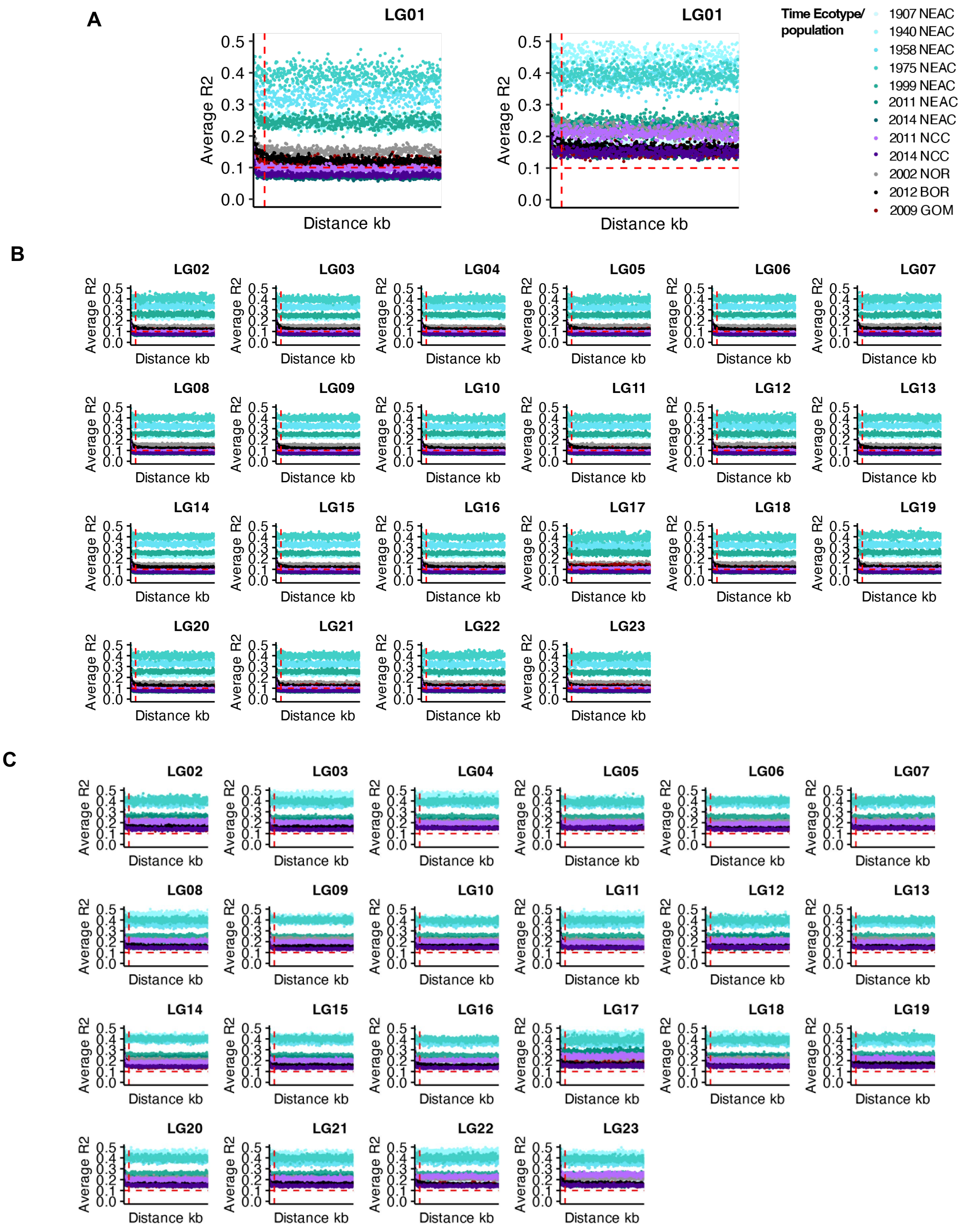

Fig. S18. LD decay plots. Plots showing average r^2 (R2) and distance in kb. Vertical dashed red line at 30kb distance and horizontal red dashed line at $r^2 = 0.1$. (**A**) showing zoom in on LG01 for full dataset and sub-sampled full dataset. (**B**) LD decay for full dataset LG02-LG23, (**C**) LD decay for sub-sampled full dataset LG02-LG23.

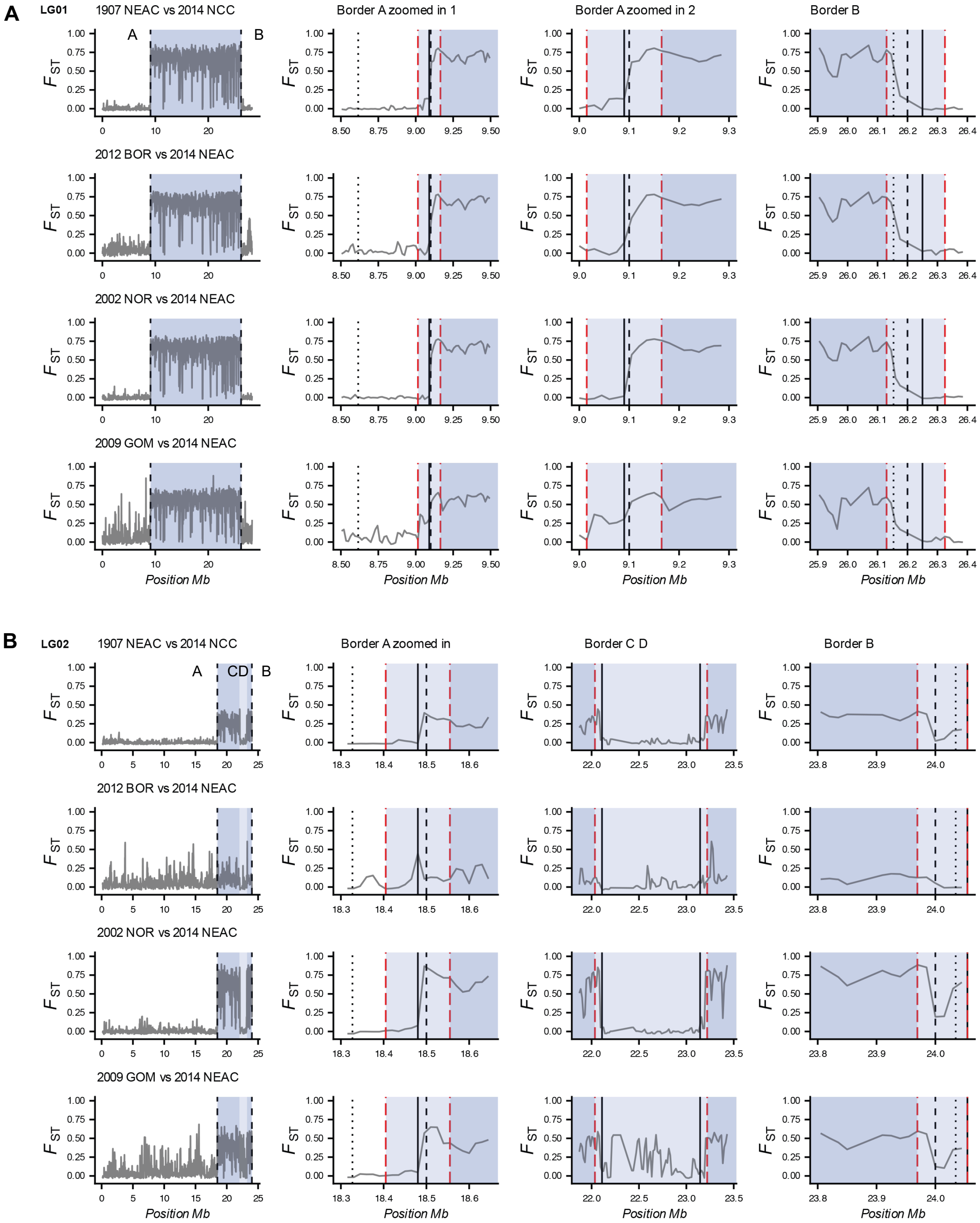

Fig. S19. F_{ST} inversion boundaries for *reduced dataset* for LG01 and LG02 with 2014 NEAC and 2014 NCC. Boundaries for dataset without 1958 and 1975 (N=192), listing NEAC comparisons versus 2014 NCC and outgroups versus 2014 NEAC, for LG01 (**A**) and LG02 (**B**). The dotted black lines show inversion boundaries (*29*) used for LD calculation (see **table S26**). Dashed black line show boundaries from another historical cod study (*163*). Both found to be imprecise for this study. The black solid line showing position of F_{ST} that confidence is set.

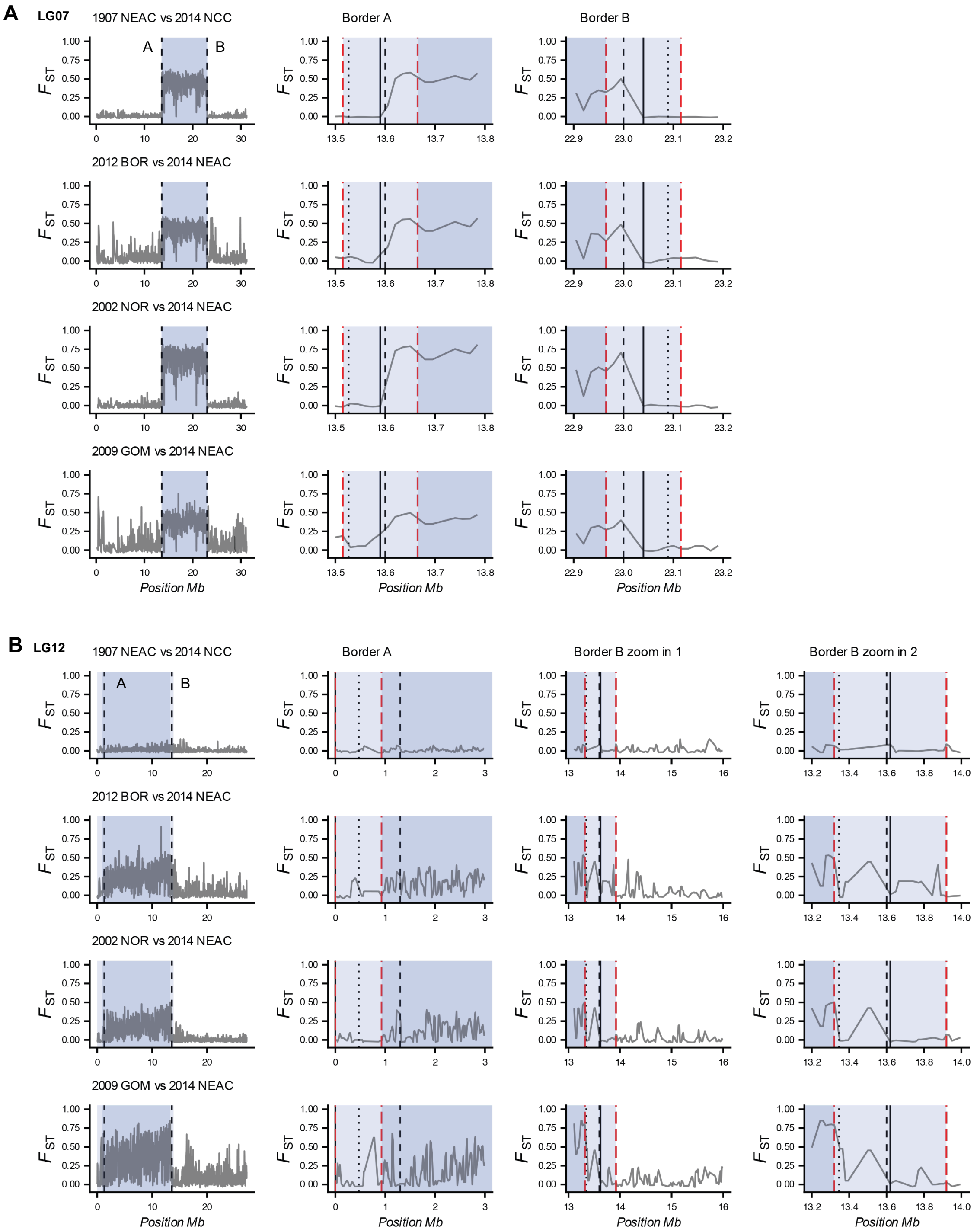

Fig. S20. F_{ST} inversion boundaries for *reduced dataset* for LG07 and LG12 with 2014 NEAC and 2014 NCC. Boundaries for dataset without 1958 and 1975 (N=192), listing NEAC comparisons versus 2014 NCC and outgroups versus 2014 NEAC, for LG07 (**A**) and LG12 (**B**). The dotted black lines show inversion boundaries (*29*) used for LD calculation (see **table S26**). Dashed black line show boundaries from another historical cod study (*163*). Both found to be imprecise for this study. The black solid line showing position of F_{ST} that confidence is set.

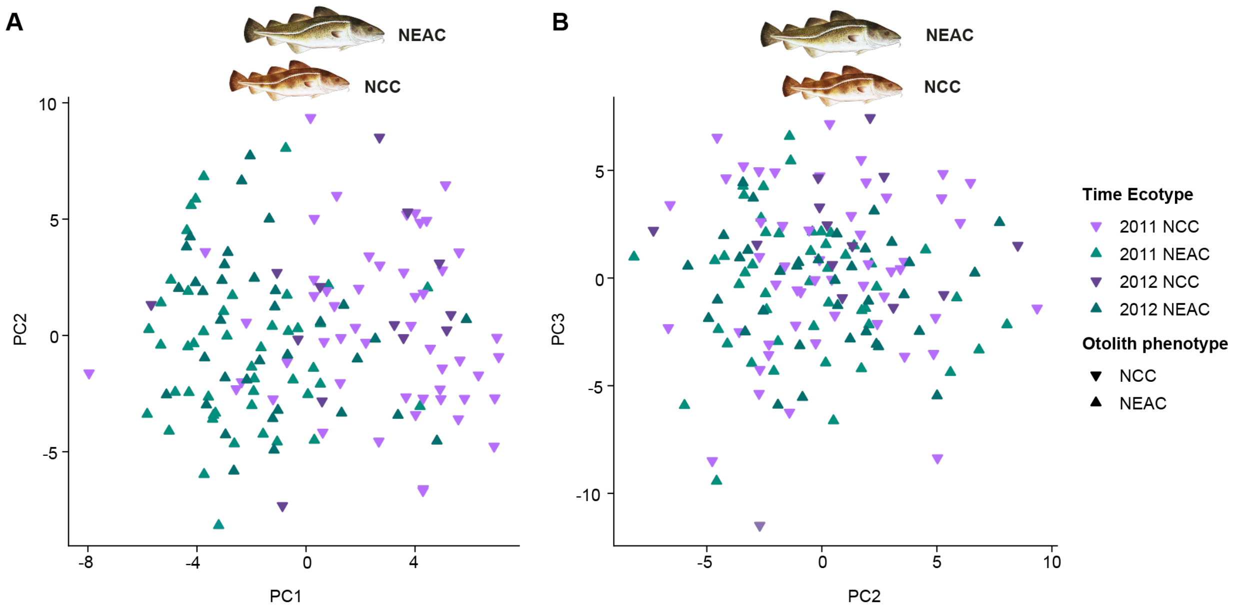

Fig. S21. PCA plots for 12k SNP chip. (**A**) for the first two principal components, (**B**) for the second and third principal component. Fish illustrations by Cecilia Helmerson (UiO).

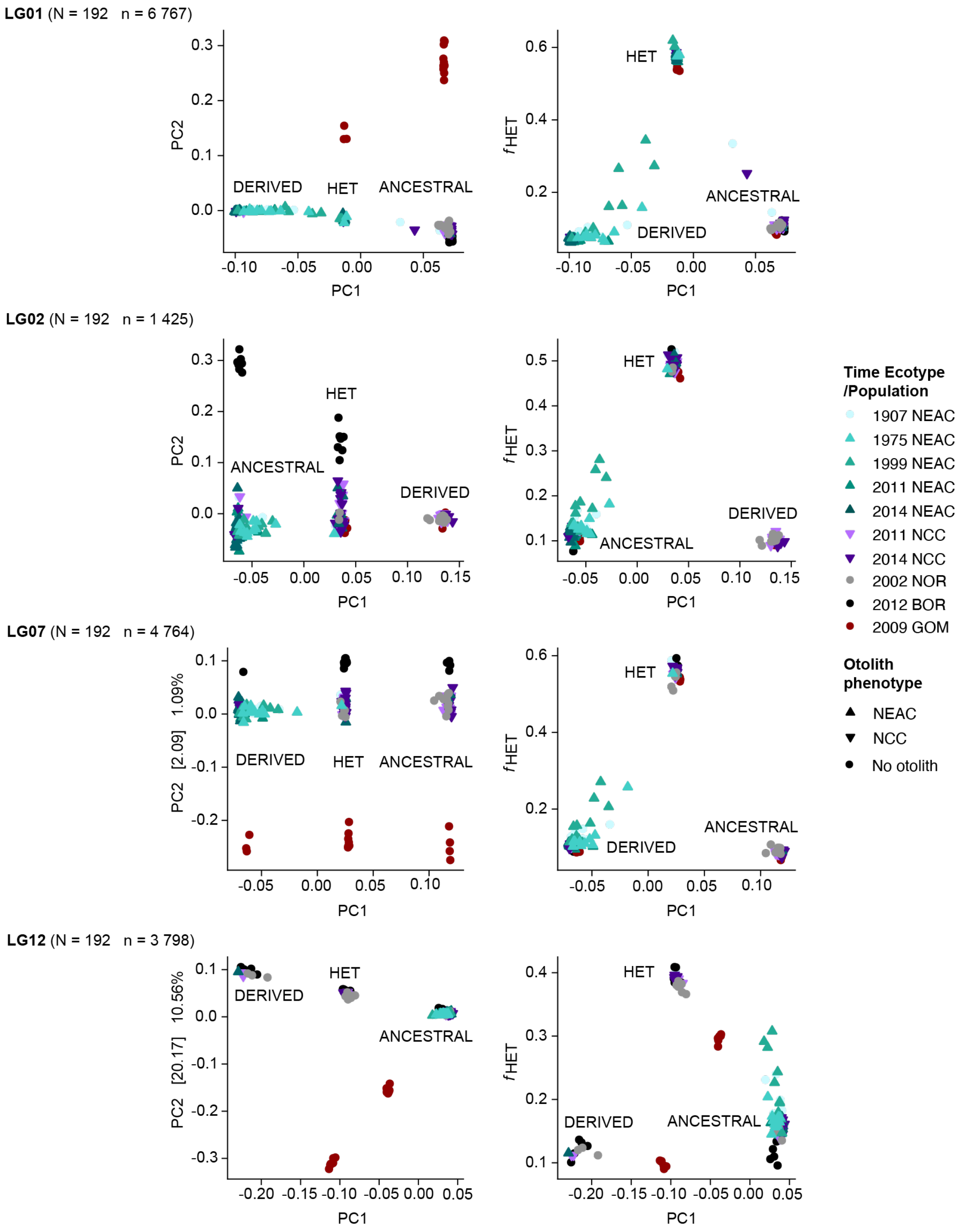

Fig. S22. Inversion scoring plots for *reduced* WGS dataset split per sampling year and population and LG. Left column showing PC1 (x-axis) and PC2 (y-axis). Right column showing PC1 versus fraction of heterozygosity f_{HET} on the y-axis. Genotypes marked as ANCESTRAL (homozygous ancestral), DERIVED (homozygous derived) and HET (heterozygous). Number of samples (N) and number of sites (n) within brackets.

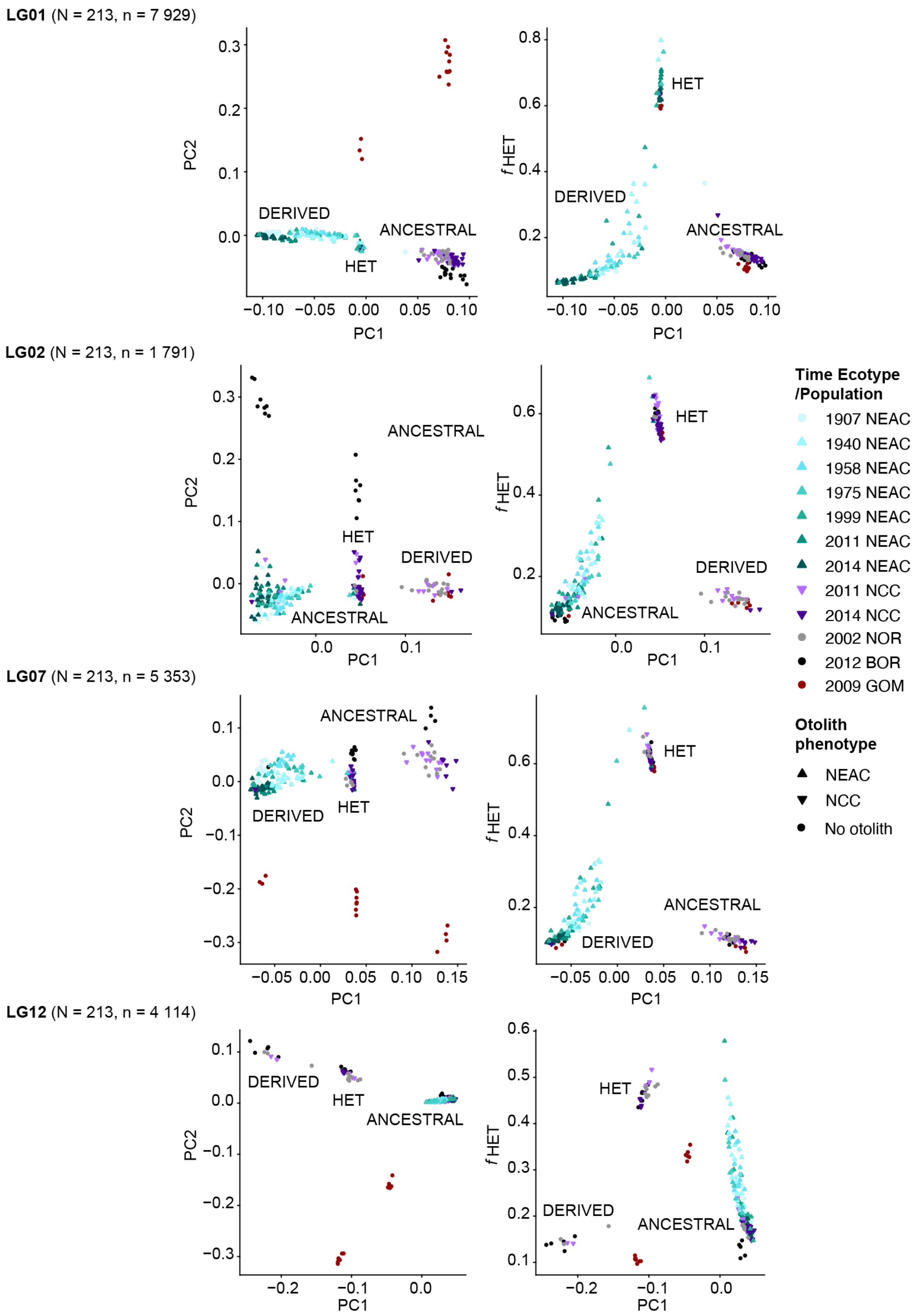

Fig. S23. Inversion scoring plots for *full* WGS dataset split per sampling year and population and LG. Left column showing PC1 (x-axis) and PC2 (y-axis). Right column showing PC1 versus fraction of heterozygosity f_{HET} on the y-axis. Genotypes marked as ANCESTRAL (homozygous ancestral), DERIVED (homozygous derived) and HET (heterozygous). Number of samples (N) and number of sites (n) within brackets.

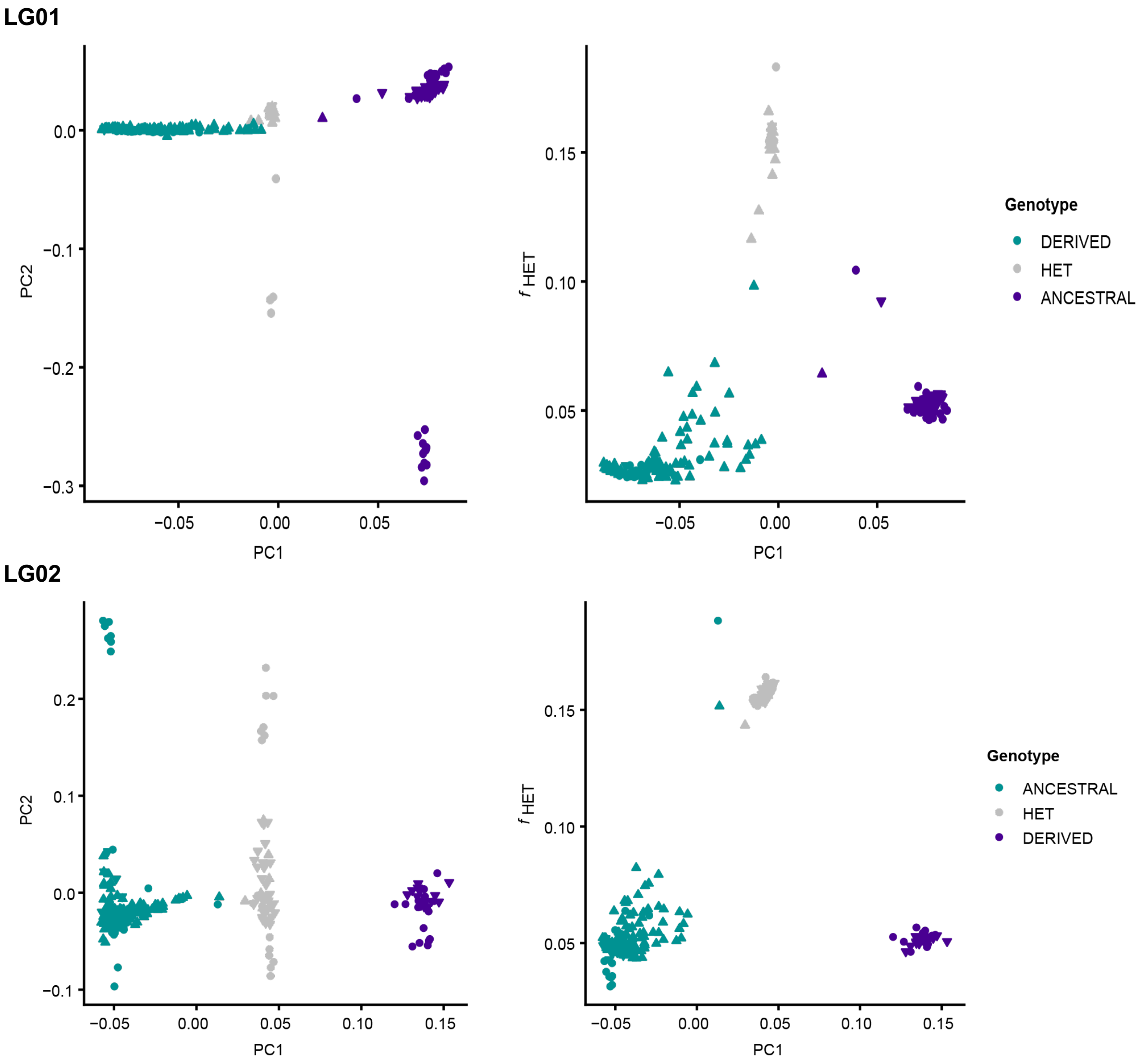

Fig. S24. Inversion scoring LG01 and LG02 plots for WGS dataset (for individuals with high amount missing data) split per sampling year, population and final genotype. Left column showing PC1 (x-axis) and PC2 (y-axis). Right column showing PC1 versus fraction of heterozygosity f_{HET} on the y-axis. Genotypes coloured as depicted on right side as ANCESTRAL (homozygous ancestral), DERIVED (homozygous derived) and HET (heterozygous), as determined by PCA and Genotype Plot.

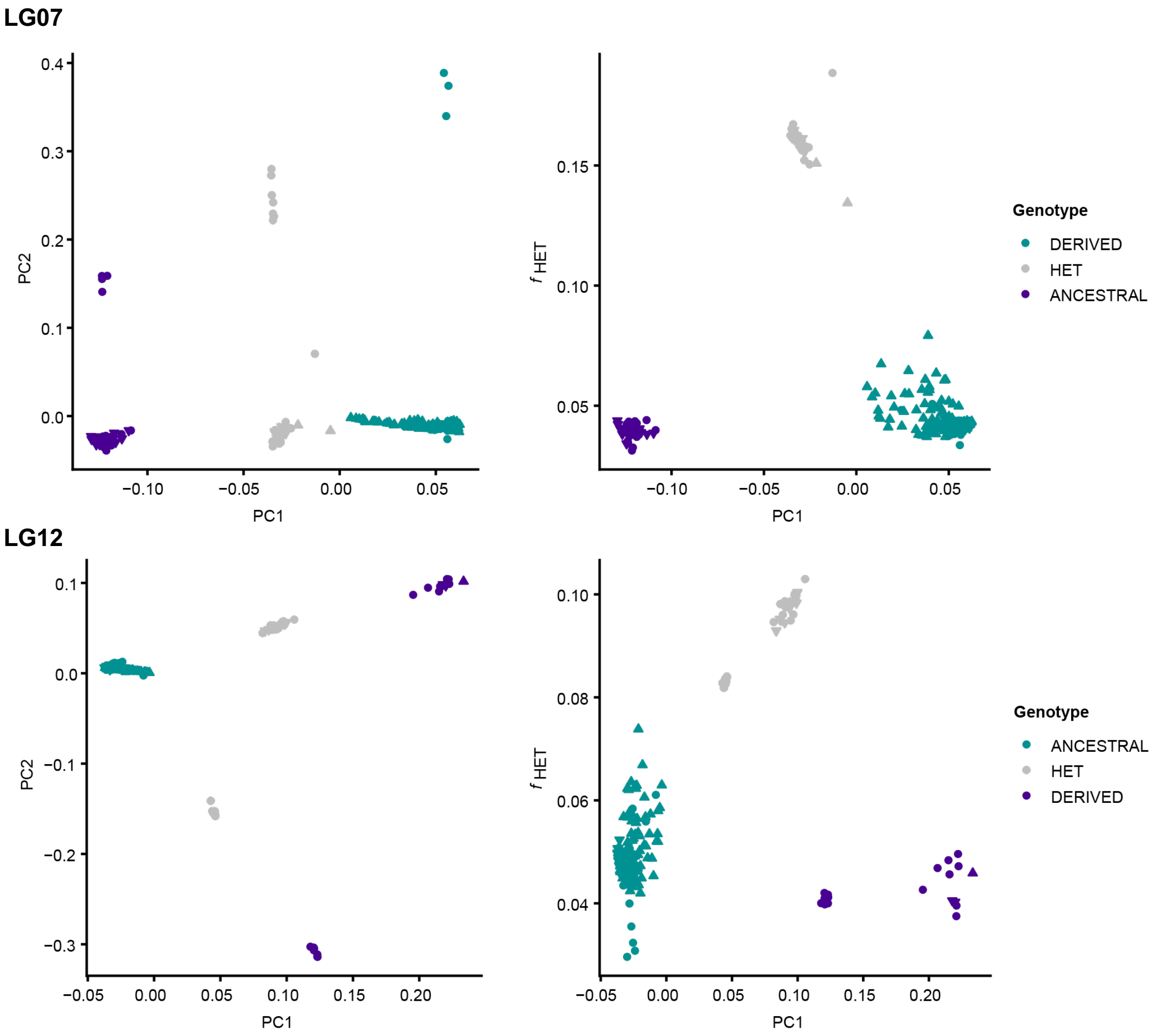

Fig. S25. Inversion scoring LG07 and LG12 plots for WGS dataset (for individuals with high amount missing data) split per sampling year, population and final genotype. Left column showing PC1 (x-axis) and PC2 (y-axis). Right column showing PC1 versus fraction of heterozygosity f_{HET} on the y-axis. Genotypes coloured as depicted on right side as ANCESTRAL (homozygous ANCESTRAL), DERIVED (homozygous derived) and HET (heterozygous), as determined by PCA and Genotype Plot.

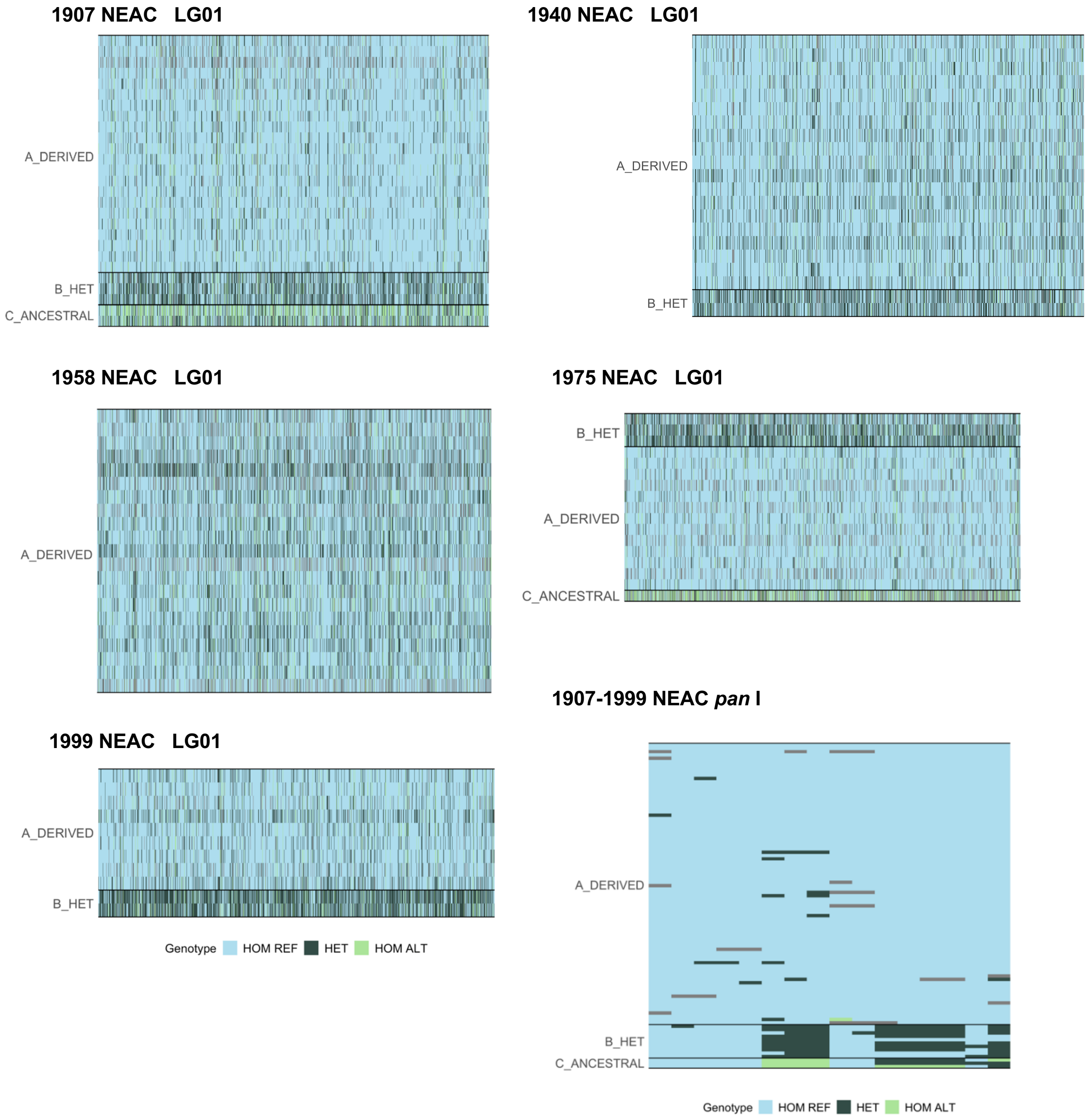

Fig. S26. Genotype plot for the inversion at LG01 for 1907, 1940, 1958, 1975 and 1999 NEAC. Showing inversion genotypes between position 9015000 and 26325000 on LG01 for historical 1907, 1940, 1958, 1975 and 1999 NEAC sorted by genotype. SNPs with > 50% missing data pruned and invariants removed. Also showing inversion genotypes between position 9436325 and 9436898 for *pan* I (panthophysin) locus on LG01 for historical NEAC (1907-1999) sorted by genotype. SNPs with > 50% missing data pruned and invariants removed. Genotype HOM REF corresponds to as homozygous in reference genome gadMor2, whereas HOM ALT corresponds to alternative genotype.

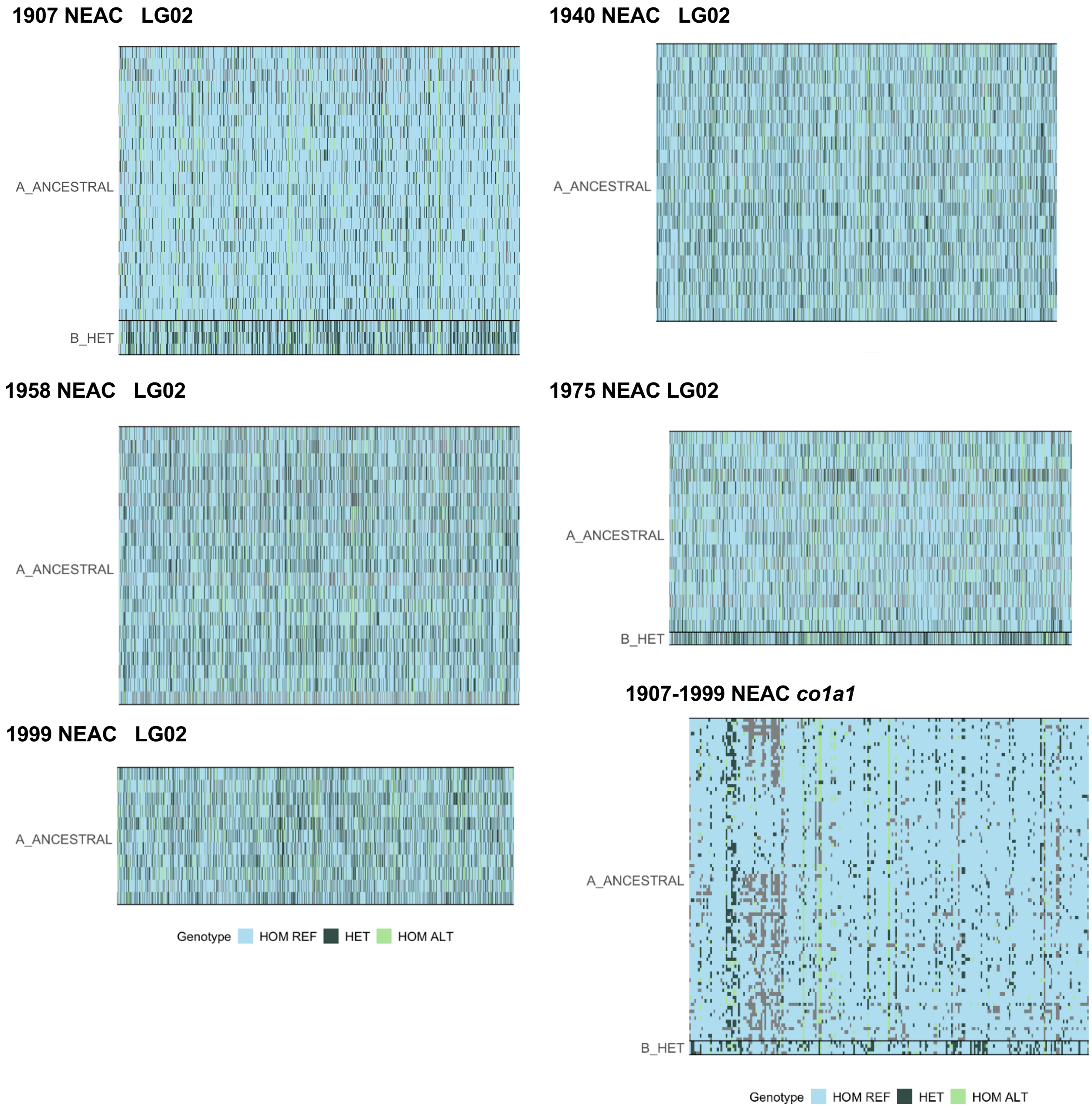

Fig. S27. Genotype plot for the inversion at LG02 for 1907, 1940, 1958, 1975 and 1999 NEAC. Showing inversion genotypes between position 18405000 and 24054406 on LG02 for historical 1907, 1940, 1958, 1975 and 1999 NEAC sorted by genotype. Also showing inversion genotypes between position 23454550 and 23470869 for collagen-alpha-1 (*co1a1*) locus on LG02 for historical NEAC (1907-1999) sorted by genotype. SNPs with > 50% missing data pruned and invariants removed. Genotype HOM REF corresponds to as homozygous in reference genome gadMor2, whereas HOM ALT corresponds to alternative genotype.

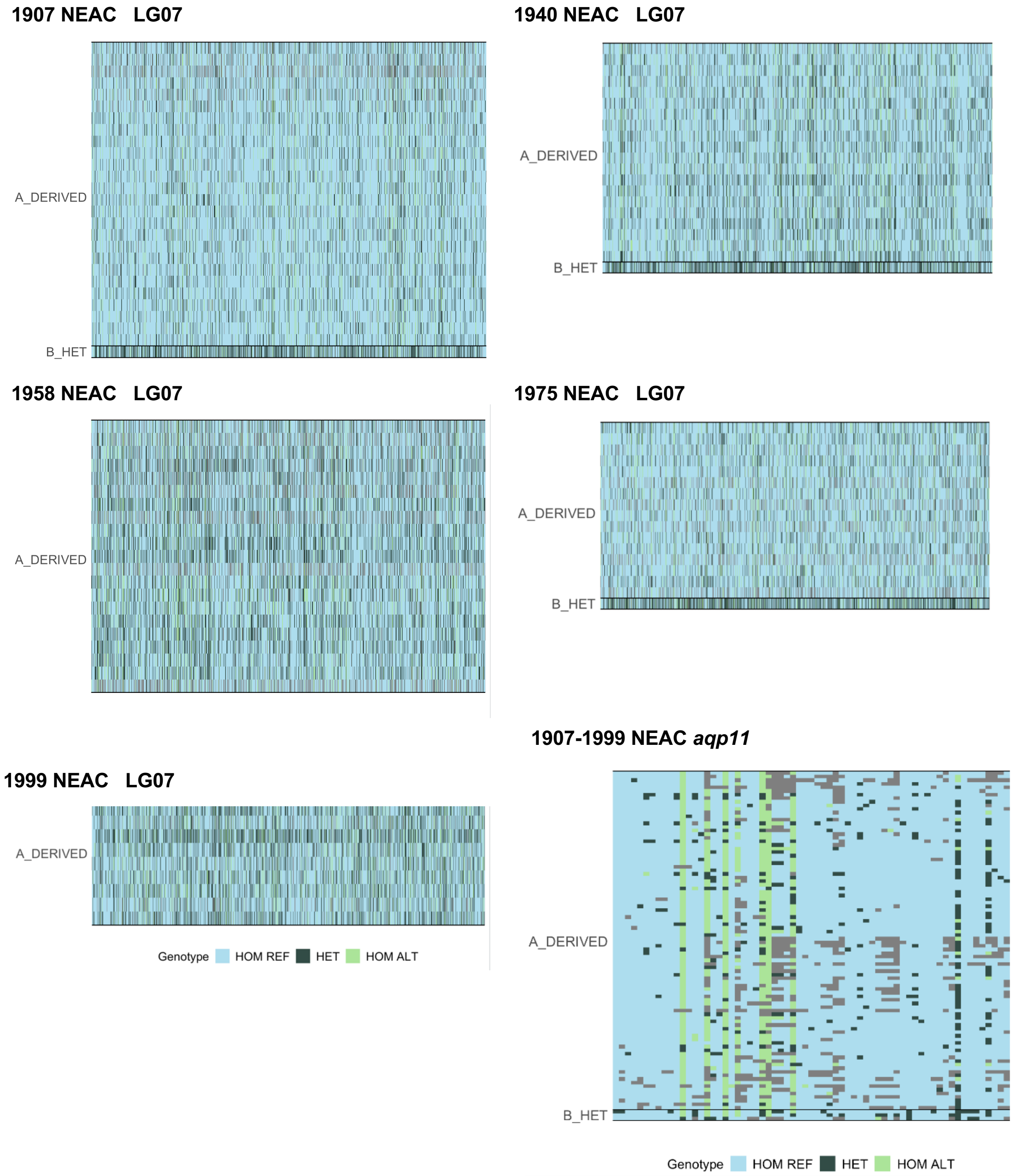

Fig. S28. Genotype plot for the inversion at LG07 for 1907, 1940, 1958, 1975 and 1999 NEAC. Showing inversion genotypes between position 13515000 and 23115000 on LG07 for historical 1907, 1940, 1958, 1975 and 1999 NEAC sorted by genotype. Also Showing inversion genotypes between position 19726653 and 19730004 for aquaporin-11 (*aqp11*) locus on LG07 for historical NEAC (1907-1999) sorted by genotype. SNPs with > 50% missing data pruned and invariants removed. Genotype HOM REF corresponds to as homozygous in reference genome gadMor2, whereas HOM ALT corresponds to alternative genotype.

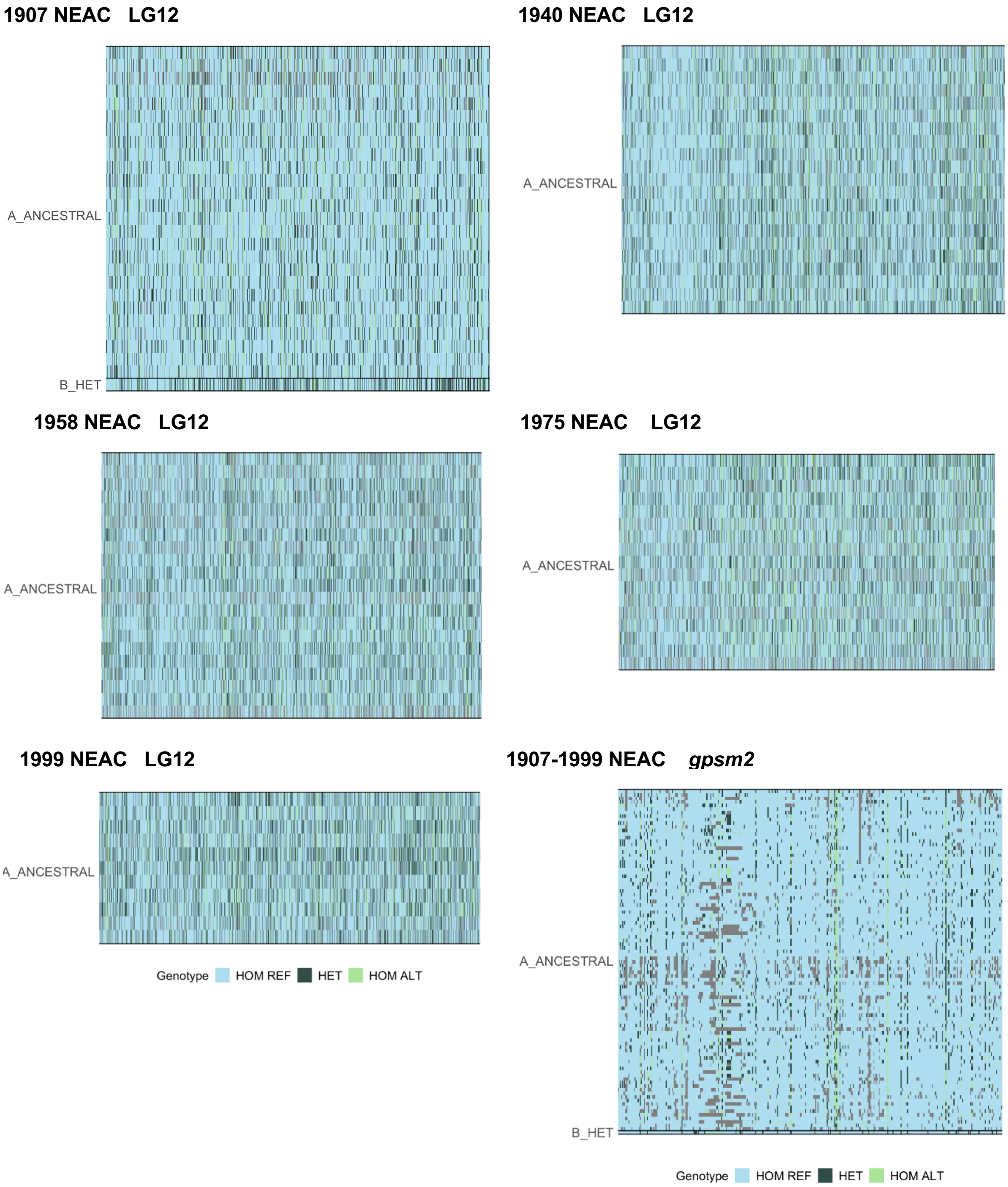

Fig. S29. Genotype plot for the inversion at LG12 for 1907, 1940, 1958, 1975 and 1999 NEAC. Showing inversion genotypes between position 1 and 13920000 on LG12 for historical 1907 and 1940 NEAC sorted by genotype. Also showing inversion genotypes between position 1194988 and 1217069 for G-protein-signalling modulator 2 (*gpsm2*) locus on LG12 for historical NEAC (1907-1999) sorted by genotype. SNPs with > 50% missing data pruned and invariants removed. Genotype HOM REF corresponds to as homozygous in reference genome gadMor2, whereas HOM ALT corresponds to alternative genotype.

Table S1. Tracy-Widom statistics for *full* WGS dataset for significant eigenvectors. Statistics obtained from Eigensoft Smartpca. N showing the number of samples and n the number of sites.

**Tracy-Widom statistics:**		**N:**	228		**n:**	70405
**Eigenvector**	**eigenvalue**	**Difference**	**TWstat**	***P* value**		**effect.n**
1	7.805437	NA	217.821		0	808.327
2	4.625239	-3.180197	285.962		0	2724.578
3	1.414334	-3.210905	36.293	2.85665e-65		10630.429

Table S2. ANOVA statistics for *full* WGS dataset for significant eigenvectors. Non-significant NEAC vs NEAC comparisons marked in bold grey text, significant NEAC vs NEAC comparisons in bold black text. Non-significant NCC vs NCC comparisons marked in bold grey italic text. Significance level given as less than 0.000001 +++ and between 0.000001 and 0.001 ***.

ANOVA statistics for population differences along each eigenvector			
**Eigenvector**	**Comp**	***P* value**	
1	overall	0	+++
1	1907 NEAC-2012 BOR	0	+++
1	**1907 NEAC-1940 NEAC**	**0.0913694**	
1	1907 NEAC-2009 GOM	0	+++
1	1907 NEAC-2011 NEAC	0.4358	
1	1907 NEAC-2011 NCC	7.72393e-12	+++
1	1907 NEAC-2014 NCC	8.88464e-09	+++
1	**1907 NEAC-2014 NEAC**	**0.00479191**	
1	1907 NEAC-2002 NOR	0.00305982	
1	**1907 NEAC-1958 NEAC**	**0.29308**	
1	**1907 NEAC-1999 NEAC**	**0.122896**	
1	**1907 NEAC-1975 NEAC**	**0.0468924**	
1	2012 BOR-1940 NEAC	0	+++
1	2012 BOR-2009 GOM	0	+++
1	2012 BOR-2011 NEAC	0	+++
1	2012 BOR-2011 NCC	0	+++
1	2012 BOR-2014 NCC	3.33067e-16	+++
1	2012 BOR-2014 NEAC	0	+++
1	2012 BOR-2002 NOR	0	+++
1	2012 BOR-1958 NEAC	1.11022e-16	+++
1	2012 BOR-1999 NEAC	0	+++
1	2012 BOR-1975 NEAC	1.11022e-16	+++
1	1940 NEAC-2009 GOM	0	+++
1	**1940 NEAC-2011 NEAC**	**0.00820507**	
1	1940 NEAC-2011 NCC	1.73195e-14	+++
1	1940 NEAC-2014 NCC	7.65986e-11	+++
1	1940 NEAC-2014 NEAC	1.14284e-05	***
1	1940 NEAC-2002 NOR	7.41736e-06	***
1	**1940 NEAC-1958 NEAC**	**0.589549**	
1	**1940 NEAC-1999 NEAC**	**0.488548**	
1	**1940 NEAC-1975 NEAC**	**0.401335**	
1	2009 GOM-2011 NEAC	0	+++
1	2009 GOM-2011 NCC	0	+++
1	2009 GOM-2014 NCC	0	+++
1	2009 GOM-2014 NEAC	0	+++
1	2009 GOM-2002 NOR	0	+++
1	2009 GOM-1958 NEAC	0	+++
1	2009 GOM-1999 NEAC	0	+++
1	2009 GOM-1975 NEAC	0	+++
1	2011 NEAC-2011 NCC	1.8852e-11	+++
1	2011 NEAC-2014 NCC	5.8847e-08	+++
1	**2011 NEAC-2014 NEAC**	**0.0226663**	
1	2011 NEAC-2002 NOR	0.00584836	
1	**2011 NEAC-1958 NEAC**	**0.0481004**	
1	**2011 NEAC-1999 NEAC**	**0.0652488**	
1	**2011 NEAC-1975 NEAC**	**0.00612858**	
1	***2011 NCC-2014 NCC***	***0.387059***	
1	2011 NCC-2014 NEAC	7.41869e-08	+++

Table S2 continued on next page

Table S2 continued from previous page

Eigenvector	Comp	*P* value	
1	2011 NCC-2002 NOR	7.61771e-06	***
1	2011 NCC-1958 NEAC	5.57532e-12	+++
1	2011 NCC-1999 NEAC	1.45409e-06	***
1	2011 NCC-1975 NEAC	1.17505e-10	+++
1	2014 NCC-2014 NEAC	3.86122e-05	***
1	2014 NCC-2002 NOR	0.00150656	
1	2014 NCC-1958 NEAC	1.28823e-08	+++
1	2014 NCC-1999 NEAC	2.29071e-05	***
1	2014 NCC-1975 NEAC	5.31991e-08	+++
1	2014 NEAC-2002 NOR	0.534199	
1	**2014 NEAC-1958 NEAC**	**0.000271797**	***
1	**2014 NEAC-1999 NEAC**	**0.00468881**	
1	**2014 NEAC-1975 NEAC**	**7.53697e-05**	***
1	2002 NOR-1958 NEAC	8.03639e-05	***
1	2002 NOR-1999 NEAC	0.012541	
1	2002 NOR-1975 NEAC	8.75513e-05	***
1	**1958 NEAC-1999 NEAC**	**0.414425**	
1	**1958 NEAC-1975 NEAC**	**0.242288**	
1	**1999 NEAC-1975 NEAC**	**0.890342**	
2	overall	0	+++
2	1907 NEAC-2012 BOR	0	+++
2	**1907 NEAC-1940 NEAC**	**0.235359**	
2	1907 NEAC-2009 GOM	0	+++
2	**1907 NEAC-2011 NEAC**	**0.494115**	
2	1907 NEAC-2011 NCC	9.34808e-14	+++
2	1907 NEAC-2014 NCC	5.96578e-12	+++
2	**1907 NEAC-2014 NEAC**	**0.120946**	
2	1907 NEAC-2002 NOR	0.000100857	***
2	**1907 NEAC-1958 NEAC**	**0.326867**	
2	**1907 NEAC-1999 NEAC**	**0.52223**	
2	**1907 NEAC-1975 NEAC**	**0.206857**	
2	2012 BOR-1940 NEAC	0	+++
2	2012 BOR-2009 GOM	0	+++
2	2012 BOR-2011 NEAC	0	+++
2	2012 BOR-2011 NCC	0	+++
2	2012 BOR-2014 NCC	0	+++
2	2012 BOR-2014 NEAC	0	+++
2	2012 BOR-2002 NOR	0	+++
2	2012 BOR-1958 NEAC	0	+++
2	2012 BOR-1999 NEAC	1.11022e-16	+++
2	2012 BOR-1975 NEAC	1.11022e-16	+++
2	1940 NEAC-2009 GOM	0	+++
2	**1940 NEAC-2011 NEAC**	**0.48527**	
2	1940 NEAC-2011 NCC	3.58795e-11	+++
2	1940 NEAC-2014 NCC	1.33698e-09	+++
2	**1940 NEAC-2014 NEAC**	**0.554494**	
2	1940 NEAC-2002 NOR	0.00164607	
2	**1940 NEAC-1958 NEAC**	**0.865593**	
2	**1940 NEAC-1999 NEAC**	**0.711164**	
2	**1940 NEAC-1975 NEAC**	**0.700273**	
2	2009 GOM-2011 NEAC	0	+++
2	2009 GOM-2011 NCC	0	+++
2	2009 GOM-2014 NCC	0	+++
2	2009 GOM-2014 NEAC	2.22045e-16	+++

Table S2 continued on next page

Table S2 continued from previous page

**Eigenvector**	**Comp**	***P* value**	
2	2009 GOM-2002 NOR	0	+++
2	2009 GOM-1958 NEAC	0	+++
2	2009 GOM-1999 NEAC	0	+++
2	2009 GOM-1975 NEAC	1.11022e-16	+++
2	2011 NEAC-2011 NCC	1.0143e-12	+++
2	2011 NEAC-2014 NCC	3.42167e-11	+++
2	**2011 NEAC-2014 NEAC**	**0.272189**	
2	2011 NEAC-2002 NOR	0.000138675	***
2	**2011 NEAC-1958 NEAC**	**0.585128**	
2	**2011 NEAC-1999 NEAC**	**0.848541**	
2	**2011 NEAC-1975 NEAC**	**0.308331**	
2	***2011 NCC-2014 NCC***	***0.186266***	
2	2011 NCC-2014 NEAC	1.71574e-09	+++
2	2011 NCC-2002 NOR	0.000149989	***
2	2011 NCC-1958 NEAC	1.37555e-09	+++
2	2011 NCC-1999 NEAC	5.51718e-08	+++
2	2011 NCC-1975 NEAC	4.1289e-07	+++
2	2014 NCC-2014 NEAC	1.18623e-07	+++
2	2014 NCC-2002 NOR	0.00289427	
2	2014 NCC-1958 NEAC	2.23253e-08	+++
2	2014 NCC-1999 NEAC	5.29647e-07	+++
2	2014 NCC-1975 NEAC	4.50848e-06	***
2	2014 NEAC-2002 NOR	0.0296121	
2	**2014 NEAC-1958 NEAC**	**0.521144**	
2	**2014 NEAC-1999 NEAC**	**0.488591**	
2	**2014 NEAC-1975 NEAC**	**0.858802**	
2	2002 NOR-1958 NEAC	0.00227647	
2	2002 NOR-1999 NEAC	0.00592173	
2	2002 NOR-1975 NEAC	0.0276621	
2	**1958 NEAC-1999 NEAC**	**0.7987**	
2	**1958 NEAC-1975 NEAC**	**0.585641**	
2	**1999 NEAC-1975 NEAC**	**0.516243**	
3	overall	3.33067e-16	+++
3	1907 NEAC-2012 BOR	0.355643	
3	**1907 NEAC-1940 NEAC**	**0.000795316**	***
3	1907 NEAC-2009 GOM	0.000329385	***
3	**1907 NEAC-2011 NEAC**	**0.000573868**	***
3	1907 NEAC-2011 NCC	0	+++
3	1907 NEAC-2014 NCC	0	+++
3	**1907 NEAC-2014 NEAC**	**0.00157506**	
3	1907 NEAC-2002 NOR	7.6728e-11	+++
3	**1907 NEAC-1958 NEAC**	**1.11465e-08**	+++
3	**1907 NEAC-1999 NEAC**	**0.000282475**	***
3	**1907 NEAC-1975 NEAC**	**0.393325**	
3	2012 BOR-1940 NEAC	0.000387862	***
3	2012 BOR-2009 GOM	0.000114813	***
3	2012 BOR-2011 NEAC	0.000558023	***
3	2012 BOR-2011 NCC	1.9984e-15	+++
3	2012 BOR-2014 NCC	4.03677e-13	+++
3	2012 BOR-2014 NEAC	0.0242913	
3	2012 BOR-2002 NOR	1.63169e-12	+++
3	2012 BOR-1958 NEAC	2.84643e-07	+++
3	2012 BOR-1999 NEAC	0.000352556	***
3	2012 BOR-1975 NEAC	0.904459	

Table S2 continued on next page

Table S2 continued from previous page

Eigenvector	Comp	*P* value	
3	1940 NEAC-2009 GOM	1.25856e-06	***
3	**1940 NEAC-2011 NEAC**	**7.4005e-08**	+++
3	1940 NEAC-2011 NCC	0	+++
3	1940 NEAC-2014 NCC	1.11022e-16	+++
3	**1940 NEAC-2014 NEAC**	**3.82249e-07**	+++
3	1940 NEAC-2002 NOR	4.62045e-11	+++
3	**1940 NEAC-1958 NEAC**	**0.00108772**	
3	**1940 NEAC-1999 NEAC**	**0.278256**	
3	**1940 NEAC-1975 NEAC**	**0.00202199**	
3	2009 GOM-2011 NEAC	0.126068	
3	2009 GOM-2011 NCC	4.96935e-11	+++
3	2009 GOM-2014 NCC	4.82417e-09	+++
3	2009 GOM-2014 NEAC	0.64626	
3	2009 GOM-2002 NOR	2.09648e-06	***
3	2009 GOM-1958 NEAC	1.50312e-08	+++
3	2009 GOM-1999 NEAC	1.29727e-05	***
3	2009 GOM-1975 NEAC	0.00179102	
3	2011 NEAC-2011 NCC	0	+++
3	2011 NEAC-2014 NCC	8.9595e-14	+++
3	**2011 NEAC-2014 NEAC**	**0.617091**	
3	2011 NEAC-2002 NOR	7.50962e-11	+++
3	**2011 NEAC-1958 NEAC**	**4.39127e-11**	+++
3	**2011 NEAC-1999 NEAC**	**4.95387e-07**	+++
3	**2011 NEAC-1975 NEAC**	**0.00607146**	
3	***2011 NCC-2014 NCC***	***0.449548***	
3	2011 NCC-2014 NEAC	4.82836e-13	+++
3	2011 NCC-2002 NOR	5.70335e-06	***
3	2011 NCC-1958 NEAC	1.11022e-16	+++
3	2011 NCC-1999 NEAC	1.01363e-13	+++
3	2011 NCC-1975 NEAC	1.85107e-12	+++
3	2014 NCC-2014 NEAC	4.953e-11	+++
3	2014 NCC-2002 NOR	0.000238242	***
3	2014 NCC-1958 NEAC	0	+++
3	2014 NCC-1999 NEAC	2.17615e-12	+++
3	2014 NCC-1975 NEAC	1.45868e-10	+++
3	2014 NEAC-2002 NOR	5.96347e-05	***
3	**2014 NEAC-1958 NEAC**	**1.11497e-10**	+++
3	**2014 NEAC-1999 NEAC**	**2.43811e-06**	***
3	**2014 NEAC-1975 NEAC**	**0.0643155**	
3	2002 NOR-1958 NEAC	2.16621e-11	+++
3	2002 NOR-1999 NEAC	2.12047e-08	+++
3	2002 NOR-1975 NEAC	1.37638e-09	+++
3	**1958 NEAC-1999 NEAC**	**0.0963775**	
3	**1958 NEAC-1975 NEAC**	**7.09128e-06**	***
3	**1999 NEAC-1975 NEAC**	**0.00206512**	

Table S3. Eigbest SNPs for *full* WGS dataset for significant eigenvectors. The top ten sites driving PCA differentiation for each principal component. Statistics obtained from Eigensoft Smartpca and matches obtained in IGV (*109*). The 21 sites that are in common with the *reduced dataset* as contributing most to PCA differentiation are marked in bold. Matches are given in relation to genetic elements in the annotated gadMor2 (*115*) genome, however not separating into introns, exons or UTRs unless stating otherwise.

Eigenvector	Site	LG	Position	n	Matches in relation to genetic elements in IGV
1	LG11:13613969	11	13613969	7.209	mRNA, GAMO_00020974-RA, PRDM13: PR domain zinc finger protein 13 (*Homo sapiens*)
**1**	**LG22:14539341**	**22**	**14539341**	**7.103**	**Protein, sp|Q6ZRP5|YD019_HUMAN**
**1**	**LG21:8608827**	**21**	**8608827**	**7.094**	**Gene, GAMO_00067756, RBM25: RNA-binding protein 25 (*Homo sapiens*)**
**1**	**LG12:23672124**	**12**	**23672124**	**6.962**	**mRNA, GAMO_00029794-RA, EEF2: Elongation factor 2 (*Gallus gallus*)**
**1**	**LG20:14383915**	**20**	**14383915**	**6.952**	**No match**
**1**	**LG23:4341139**	**23**	**4341139**	**6.853**	**Match, LG23: hit:109464:4.5.0.43, genemark-LG23-abinit-gene-43.15-mRNA-1**
**1**	**LG14:21632665**	**14**	**21632665**	**6.823**	**Protein, sp|Q69566|U88_HHV6U**
**1**	**LG09:20786400**	**9**	**20786400**	**6.779**	**No match**
**1**	**LG09:9086716**	**9**	**9086716**	**6.764**	**Protein, sp|Q69566|U88_HHV6U**
**1**	**LG16:25991457**	**16**	**25991457**	**6.712**	**mRNA, GAMO_00011131-RA, yap1: Transcriptional coactivator YAP1 (*Danio rerio*)**
**2**	**LG07:23819790**	**7**	**23819790**	**8.740**	**Gene, GAMO_00007041, Pigment epithelium-derived factor (*Mus musculus*)**
**2**	**LG21:8590042**	**21**	**8590042**	**8.372**	**No match**
**2**	**LG11:20291131**	**11**	**20291131**	**8.046**	**Match, LG11: hit:150416:4.5.0.203, genemark-LG11-abinit-gene-203.8-mRNA-1**
**2**	**LG20:15065251**	**20**	**15065251**	**7.926**	**Protein, sp|Q69566|U88_HHV6U**
**2**	**LG21:4089335**	**21**	**4089335**	**7.898**	**No match**
**2**	**LG08:9865422**	**8**	**9865422**	**7.800**	**mRNA, GAMO_00031539-RA, ATP6V0B: V-type proton ATPase 21 kDa proteolipid subunit (*Bos taurus*)**
**2**	**LG21:4201054**	**21**	**4201054**	**7.688**	**Gene, GAMO_00067189, NEK2: Serine/threonine-protein kinase Nek2 (*Homo sapiens*)**
2	LG09:24151414	9	24151414	7.660	Gene, GAMO_00047059, CPT1A: Carnitine O-palmitoyltransferase 1, liver isoform (*Homo sapiens*)
2	LG18:17204468	18	17204468	7.647	Match, LG18: hit:121025:4.5.0.172, genemark-LG18-abinit-gene-172.29-mRNA-1
2	LG11:23665796	11	23665796	7.535	Match, LG11: hit:152139:4.5.0.237, snap_masked-LG11-abinit-gene-237.52-mRNA-1
3	LG20:18730188	20	18730188	7.621	Protein, sp|Q69566|U88_HHV6U
**3**	**LG20:18717595**	**20**	**18717595**	**7.594**	**Protein, sp|Q69566|U88_HHV6U**
**3**	**LG18:17157756**	**18**	**17157756**	**6.384**	**mRNA, GAMO_00065789-RA, PEMT: Phosphatidylethanolamine N-methyltransferase (*Bos taurus*)**
**3**	**LG20:18720288**	**20**	**18720288**	**6.258**	**Protein, sp|Q69566|U88_HHV6U**
**3**	**LG11:9655819**	**11**	**9655819**	**5.789**	**mRNA, GAMO_00020456-RA, MTMR10: Myotubularin-related protein 10 (*Homo sapiens*)**
**3**	**LG20:18712505**	**20**	**18712505**	**5.173**	**Protein, sp|Q6ZRP5|YD019_HUMAN**
3	LG18:17118405	18	17118405	5.060	Expressed sequence match, LG18: hit:100405:3.2.0.171
3	LG18:17165658	18	17165658	4.547	Match, LG18: hit:121036:4.5.0.172, snap_masked-LG18-abinit-gene-172.40-mRNA-1
3	LG22:15335073	22	15335073	4.310	mRNA, GAMO_00071809-RA, PTPN2: Tyrosine-protein phosphatase non-receptor type 2 (*Homo sapiens*)
3	LG04:23127169	4	23127169	4.041	Match, LG04: hit:192088:4.5.0.231, genemark-LG04-abinit-gene-231.13-mRNA-1

Table S4. Tracy-Widom statistics for *reduced* WGS dataset for significant eigenvectors. Statistics obtained from Eigensoft Smartpca. N showing the number of samples and n the number of sites.

	**Tracy-Widom statistics:**	**N:**	192	**n:**	141549
**Eigenvector**	**Eigenvalue**	**Difference**	**TWstat**	***P* value**	**effect.n**
1	6.472447	NA	188.019	0	896.734
2	3.992223	-2.480224	255.637	0	3127.286
3	1.233829	-2.758395	21.580	6.17007e-31	18019.550

Table S5. ANOVA statistics for *reduced* WGS dataset for significant eigenvectors. Non-significant NEAC vs NEAC comparisons marked in bold grey text, significant NEAC vs NEAC comparisons in bold black text. Non-significant NCC vs NCC comparisons marked in bold grey italic text. Significance level given as less than 0.000001 +++ and between 0.000001 and 0.001 ***.

**ANOVA statistics for population differences along each eigenvector**			
**Eigenvector**	**Comp**	***P* value**	
1	overall	0	+++
1	1907 NEAC-2012 BOR	0	+++
1	1907 NEAC-2009 GOM	0	+++
1	**1907 NEAC-2011 NEAC**	**0.242127**	
1	1907 NEAC-2011 NCC	1.46549e-14	+++
1	1907 NEAC-2014 NCC	2.77572e-11	+++
1	**1907 NEAC-2014 NEAC**	**0.00201059**	
1	1907 NEAC-2002 NOR	5.23639e-05	***
1	**1907 NEAC-1999 NEAC**	**0.107289**	
1	**1907 NEAC-1975 NEAC**	**0.0565277**	
1	2012 BOR-2009 GOM	0	+++
1	2012 BOR-2011 NEAC	0	+++
1	2012 BOR-2011 NCC	0	+++
1	2012 BOR-2014 NCC	1.11022e-16	+++
1	2012 BOR-2014 NEAC	0	+++
1	2012 BOR-2002 NOR	0	+++
1	2012 BOR-1999 NEAC	1.11022e-16	+++
1	2012 BOR-1975 NEAC	1.11022e-16	+++
1	2009 GOM-2011 NEAC	0	+++
1	2009 GOM-2011 NCC	0	+++
1	2009 GOM-2014 NCC	0	+++
1	2009 GOM-2014 NEAC	0	+++
1	2009 GOM-2002 NOR	0	+++
1	2009 GOM-1999 NEAC	0	+++
1	2009 GOM-1975 NEAC	0	+++
1	2011 NEAC-2011 NCC	8.90399e-14	+++
1	2011 NEAC-2014 NCC	4.41114e-10	+++
1	**2011 NEAC-2014 NEAC**	**0.0281048**	
1	2011 NEAC-2002 NOR	0.000163009	***
1	**2011 NEAC-1999 NEAC**	**0.0358509**	
1	**2011 NEAC-1975 NEAC**	**0.00421292**	
1	***2011 NCC-2014 NCC***	***0.224603***	
1	2011 NCC-2014 NEAC	2.32578e-10	+++
1	2011 NCC-2002 NOR	1.80872e-06	***
1	2011 NCC-1999 NEAC	5.52e-08	+++
1	2011 NCC-1975 NEAC	6.86484e-12	+++
1	2014 NCC-2014 NEAC	4.14597e-07	+++
1	2014 NCC-2002 NOR	0.000754429	***
1	2014 NCC-1999 NEAC	1.31301e-06	***
1	2014 NCC-1975 NEAC	2.07505e-09	+++
1	2014 NEAC-2002 NOR	0.0766395	
1	**2014 NEAC-1999 NEAC**	**0.00226765**	
1	**2014 NEAC-1975 NEAC**	**6.82408e-05**	***
1	2002 NOR-1999 NEAC	0.00211805	
1	2002 NOR-1975 NEAC	1.00023e-05	***
1	**1999 NEAC-1975 NEAC**	**0.864196**	

Table S5 continued on next page

Table S5 continued from previous page

Eigenvector	Comp	*P* value	
2	overall	0	+++
2	1907 NEAC-2012 BOR	0	+++
2	1907 NEAC-2009 GOM	0	+++
2	**1907 NEAC-2011 NEAC**	**0.795326**	
2	1907 NEAC-2011 NCC	2.41135e-11	+++
2	1907 NEAC-2014 NCC	2.80892e-09	+++
2	**1907 NEAC-2014 NEAC**	**0.707956**	
2	1907 NEAC-2002 NOR	0.00222083	
2	**1907 NEAC-1999 NEAC**	**0.54164**	
2	**1907 NEAC-1975 NEAC**	**0.0816575**	
2	2012 BOR-2009 GOM	0	+++
2	2012 BOR-2011 NEAC	0	+++
2	2012 BOR-2011 NCC	0	+++
2	2012 BOR-2014 NCC	1.11022e-16	+++
2	2012 BOR-2014 NEAC	0	+++
2	2012 BOR-2002 NOR	0	+++
2	2012 BOR-1999 NEAC	0	+++
2	2012 BOR-1975 NEAC	0	+++
2	2009 GOM-2011 NEAC	0	+++
2	2009 GOM-2011 NCC	0	+++
2	2009 GOM-2014 NCC	0	+++
2	2009 GOM-2014 NEAC	0	+++
2	2009 GOM-2002 NOR	0	+++
2	2009 GOM-1999 NEAC	0	+++
2	2009 GOM-1975 NEAC	0	+++
2	2011 NEAC-2011 NCC	4.27991e-12	+++
2	2011 NEAC-2014 NCC	2.08965e-10	+++
2	**2011 NEAC-2014 NEAC**	**0.506854**	
2	2011 NEAC-2002 NOR	0.000150964	***
2	2011 NEAC-1999 NEAC	0.294687	
2	**2011 NEAC-1975 NEAC**	**0.0185656**	
2	***2011 NCC-2014 NCC***	***0.132724***	
2	2011 NCC-2014 NEAC	2.20402e-10	+++
2	2011 NCC-2002 NOR	0.000140485	***
2	2011 NCC-1999 NEAC	1.14083e-06	***
2	2011 NCC-1975 NEAC	4.78443e-05	***
2	2014 NCC-2014 NEAC	2.38727e-08	+++
2	2014 NCC-2002 NOR	0.00395604	
2	2014 NCC-1999 NEAC	1.578e-05	***
2	2014 NCC-1975 NEAC	0.000738046	***
2	2014 NEAC-2002 NOR	0.00723053	
2	**2014 NEAC-1999 NEAC**	**0.768091**	
2	**2014 NEAC-1975 NEAC**	**0.157644**	
2	2002 NOR-1999 NEAC	0.0336172	
2	2002 NOR-1975 NEAC	0.328848	
2	**1999 NEAC-1975 NEAC**	**0.296776**	
3	overall	3.33067e-16	+++
3	1907 NEAC-2012 BOR	0.0004446	***
3	1907 NEAC-2009 GOM	1.96949e-07	+++
3	**1907 NEAC-2011 NEAC**	**0.0533918**	
3	1907 NEAC-2011 NCC	0	+++
3	1907 NEAC-2014 NCC	7.77156e-15	+++
3	**1907 NEAC-2014 NEAC**	**0.14352**	
3	1907 NEAC-2002 NOR	4.73386e-07	+++

Table S5 continued on next page

Table S5 continued from previous page

Eigenvector	Comp	*P* value	
3	**1907 NEAC-1999 NEAC**	**0.260028**	
3	**1907 NEAC-1975 NEAC**	**0.253241**	
3	2012 BOR-2009 GOM	7.46027e-06	***
3	2012 BOR-2011 NEAC	0.00533628	
3	2012 BOR-2011 NCC	6.75481e-10	+++
3	2012 BOR-2014 NCC	2.00326e-08	+++
3	2012 BOR-2014 NEAC	0.160595	
3	2012 BOR-2002 NOR	0.000279264	***
3	2012 BOR-1999 NEAC	1.72467e-06	***
3	2012 BOR-1975 NEAC	0.00135588	
3	2009 GOM-2011 NEAC	8.29797e-08	+++
3	2009 GOM-2011 NCC	4.85099e-07	+++
3	2009 GOM-2014 NCC	6.14132e-06	***
3	2009 GOM-2014 NEAC	0.00248239	
3	2009 GOM-2002 NOR	0.469221	
3	2009 GOM-1999 NEAC	2.5043e-09	+++
3	2009 GOM-1975 NEAC	9.1808e-08	+++
3	2011 NEAC-2011 NCC	2.26485e-14	+++
3	2011 NEAC-2014 NCC	1.64069e-12	+++
3	**2011 NEAC-2014 NEAC**	**0.978109**	
3	2011 NEAC-2002 NOR	6.15967e-07	+++
3	**2011 NEAC-1999 NEAC**	**0.00154985**	
3	**2011 NEAC-1975 NEAC**	**0.617705**	
3	***2011 NCC-2014 NCC***	***0.994324***	
3	2011 NCC-2014 NEAC	2.82629e-12	+++
3	2011 NCC-2002 NOR	1.15296e-07	+++
3	2011 NCC-1999 NEAC	1.67117e-11	+++
3	2011 NCC-1975 NEAC	8.77372e-10	+++
3	2014 NCC-2014 NEAC	4.53968e-11	+++
3	2014 NCC-2002 NOR	1.7359e-06	***
3	2014 NCC-1999 NEAC	5.52344e-10	+++
3	2014 NCC-1975 NEAC	2.18698e-08	+++
3	2014 NEAC-2002 NOR	0.00425784	
3	**2014 NEAC-1999 NEAC**	**0.0592881**	
3	**2014 NEAC-1975 NEAC**	**0.781912**	
3	2002 NOR-1999 NEAC	9.52838e-09	+++
3	2002 NOR-1975 NEAC	1.03887e-06	***
3	**1999 NEAC-1975 NEAC**	**0.0152957**	

Table S6. Eigbest SNPs for *reduced* WGS dataset for significant eigenvectors. Statistics obtained from Eigensoft Smartpca and matches obtained in IGV (*109*). The 21 sites that are in common with the *full* dataset as contributing most to PCA differentiation are marked in bold. Matches are given in relation to genetic elements in the annotated gadMor2 genome, however not separating into introns, exons or UTRs unless stating otherwise.

Eigenvector	Site	LG	Position	n	Matches in relation to genetic elements in IGV
**1**	**LG12:23672124**	**12**	**23672124**	**7.163**	**mRNA, GAMO_00029794-RA, EEF2: Elongation factor 2 (*Gallus gallus*)**
**1**	**LG22:14539341**	**22**	**14539341**	**7.095**	**Protein, sp|Q6ZRP5|YD019_HUMAN**
**1**	**LG21:8608827**	**21**	**8608827**	**7.051**	**Gene, GAMO_00067756, RBM25: RNA-binding protein 25 (*Homo sapiens*)**
**1**	**LG20:14383915**	**20**	**14383915**	**6.882**	**No match**
**1**	**LG23:4341139**	**23**	**4341139**	**6.871**	**Match, LG23: hit:109464:4.5.0.43, genemark-LG23-abinit-gene-43.15-mRNA-1**
**1**	**LG14:21632665**	**14**	**21632665**	**6.766**	**Protein, sp|Q69566|U88_HHV6U**
**1**	**LG09:9086716**	**9**	**9086716**	**6.733**	**Protein, sp|Q69566|U88_HHV6U**
**1**	**LG09:20786400**	**9**	**20786400**	**6.712**	**No match**
**1**	**LG16:25991457**	**16**	**25991457**	**6.705**	**mRNA, GAMO_00011131-RA, yap1: Transcriptional coactivator YAP1 (*Danio rerio*)**
1	LG03:25146860	3	25146860	6.658	Gene, GAMO_00014973, KRT222: Keratin-like protein KRT222 (*Bos taurus*)
**2**	**LG07:23819790**	**7**	**23819790**	**8.415**	**Gene, GAMO_00007041, Pigment epithelium-derived factor (*Mus musculus*)**
**2**	**LG21:8590042**	**21**	**8590042**	**8.291**	**No match**
2	LG02:3761996	2	3761996	8.043	Expressed sequence match, LG02: hit:100271:3.2.0.37, c283378_g1_i4
2	LG11:20349462	11	20349462	7.791	No match
**2**	**LG21:4089335**	**21**	**4089335**	**7.771**	**No match**
**2**	**LG20:15065251**	**20**	**15065251**	**7.701**	**Protein, sp|Q69566|U88_HHV6U**
**2**	**LG11:20291131**	**11**	**20291131**	**7.678**	**Match, LG11: hit:150416:4.5.0.203, genemark-LG11-abinit-gene-203.8-mRNA-1**
**2**	**LG08:9865422**	**8**	**9865422**	**7.637**	**mRNA, GAMO_00031539-RA, ATP6V0B: V-type proton ATPase 21 kDa proteolipid subunit (*Bos taurus*)**
**2**	**LG21:4201054**	**21**	**4201054**	**7.500**	**Gene, GAMO_00067189, NEK2: Serine/threonine-protein kinase Nek2 (*Homo sapiens*)**
2	LG18:17239074	18	17239074	7.482	mRNA, GAMO_00065806-RA, ADAP1: Arf-GAP with dual PH domain-containing protein 1 *(Homo sapiens*)
3	LG20:18717008	20	18717008	10.921	Protein, sp|Q69566|U88_HHV6U
**3**	**LG20:18717595**	**20**	**18717595**	**9.209**	**Protein, sp|Q69566|U88_HHV6U**
**3**	**LG18:17157756**	**18**	**17157756**	**7.658**	**mRNA, GAMO_00065789-RA, PEMT: Phosphatidylethanolamine N-methyltransferase (*Bos taurus*)**
**3**	**LG11:9655819**	**11**	**9655819**	**6.877**	**mRNA, GAMO_00020456-RA, MTMR10: Myotubularin-related protein 10 (*Homo sapiens*)**
**3**	**LG20:18712505**	**20**	**18712505**	**5.652**	**Protein, sp|Q6ZRP5|YD019_HUMAN**
**3**	**LG20:18720288**	**20**	**18720288**	**5.437**	**Protein, sp|Q69566|U88_HHV6U**
3	LG11:9633621	11	9633621	5.382	Match, LG11: hit:145209:4.5.0.96, genemark-LG11-abinit-gene-96.16-mRNA-1
3	LG15:5623002	15	5623002	5.217	No match
3	LG10:16564746	10	16564746	5.142	No match
3	LG11:14935961	11	14935961	5.105	mRNA, GAMO_00021092-RA, Znf516: Zinc finger protein 516 (*Mus musculus*)

Table S7. Coastal fraction *full* and *reduced* WGS dataset split per sampling year and population. Coastal fraction in admixture at k=5, without straying individuals (NEAC) and bycatch individuals (coastal cod during the spawning season). Observe that *full dataset* is given with proportions when removing individuals with >30% missing data.

Dataset	Year/population	Average NCC	SD	N
full <30% miss	1907 NEAC	0.09	0.12	26
	1940 NEAC	0.05	0.07	16
	1958 NEAC	0.02	0.04	13
	1975 NEAC	0.05	0.06	10
	1999 NEAC	0.06	0.10	10
	2011 NEAC	0.14	0.11	21
	2014 NEAC	0.12	0.12	22
	2011 NCC	0.78	0.26	24
	2014 NCC	0.65	0.25	23
	2002 NOR	0.17	0.26	15
	2012 BOR	0.00	0.00	15
	2009 GOM	0.00	0.00	14
reduced	1907 NEAC	0.07	0.12	27
	1975 NEAC	0.03	0.07	11
	1999 NEAC	0.03	0.05	11
	2011 NEAC	0.12	0.10	22
	2014 NEAC	0.10	0.11	22
	2011 NCC	0.77	0.26	24
	2014 NCC	0.67	0.26	23
	2002 NOR	0.06	0.11	15
	2012 BOR	0.00	0.00	15
	2009 GOM	0.00	0.00	14

Table S8. Coastal fraction for NEAC in *full* and *reduced* WGS dataset split per birth year classes with more restrictive filtering in *full*. Coastal fraction in admixture at k=5 for year classes (3 years pooled). Note that statistics for full is given when removing individuals with >30% missing data.

Dataset	Year classes	Mean	Max	Min	Median	Std
full <30% miss	1895-1897	0.0537	0.147	0.00001	0.0350	0.0574
	1898-1900	0.0701	0.150	0.00001	0.0652	0.0767
	1901-1903	0.156	0.493	0.00001	0.0923	0.169
	1904-1906	0.00001	0.00001	0.00001	0.00001	NA
	1918-1920	0.00001	0.00001	0.00001	0.00001	NA
	1928-1930	0.0508	0.256	0.00001	0.0358	0.0729
	1931-1933	0.0698	0.103	0.0369	0.0698	0.0466
	1943-1945	0.0426	0.0852	0.00001	0.0426	0.0602
	1946-1948	0.0183	0.128	0.00001	0.00001	0.0485
	1949-1951	0.00001	0.00001	0.00001	0.00001	0
	1967-1969	0.0522	0.141	0.00001	0.0203	0.0622
	1990-1992	0.0568	0.260	0.00001	0.00001	0.0968
	1998-2000	0.0619	0.107	0.00001	0.0791	0.0553
	2001-2003	0.158	0.418	0.00001	0.168	0.127
	2004-2006	0.118	0.320	0.00001	0.117	0.0986
reduced	1895-1897	0.0226	0.137	0.00001	0.00001	0.0490
	1898-1900	0.0388	0.108	0.00001	0.0238	0.0511
	1901-1903	0.151	0.448	0.00001	0.106	0.163
	1904-1906	0.00001	0.00001	0.00001	0.00001	NA
	1967-1969	0.0258	0.214	0.00001	0.00001	0.0659
	1990-1992	0.0343	0.139	0.00001	0.00001	0.0480
	1998-2000	0.0372	0.0601	0.00001	0.0516	0.0325
	2001-2003	0.167	0.405	0.00001	0.154	0.109
	2004-2006	0.0727	0.273	0.00001	0.00001	0.0922

Table S9. Post-hoc test *P* values of Coastal fraction in NEAC for *full* WGS dataset split per birth year classes with more restrictive filtering. Note that statistics for full is given when removing individuals with >30% missing data. The lowest *P* values are marked on brown.

Year classes	1895-1897	1898-1900	1901-1903	1904-1906	1918-1920	1928-1930	1931-1933	1943-1945	1946-1948	1949-1951	1967-1969	1990-1992	1998-2000	2001-2003
1898-1900	0.92	-	-	-	-	-	-	-	-	-	-	-	-	-
1901-1903	0.57	0.78	-	-	-	-	-	-	-	-	-	-	-	-
1904-1906	0.77	0.79	0.66	-	-	-	-	-	-	-	-	-	-	-
1918-1920	0.77	0.79	0.66	-	-	-	-	-	-	-	-	-	-	-
1928-1930	0.89	0.79	0.46	0.79	0.79	-	-	-	-	-	-	-	-	-
1931-1933	0.91	1.00	1.00	0.89	0.89	0.79	-	-	-	-	-	-	-	-
1943-1945	0.97	0.89	0.78	1.00	1.00	1.00	0.89	-	-	-	-	-	-	-
1946-1948	0.41	0.46	0.31	1.00	1.00	0.61	0.48	0.86	-	-	-	-	-	-
1949-1951	0.38	0.41	0.31	-	-	0.46	0.40	0.72	0.87	-	-	-	-	-
1967-1969	0.91	0.86	0.46	0.79	0.79	0.99	0.91	1.00	0.57	0.48	-	-	-	-
1990-1992	0.79	0.79	0.40	0.89	0.89	0.89	0.79	1.00	0.79	0.71	0.89	-	-	-
1998-2000	1.00	0.91	0.82	0.89	0.89	0.79	1.00	1.00	0.69	0.48	0.99	0.89	-	-
2001-2003	0.36	0.57	0.99	0.68	0.68	0.34	0.79	0.65	0.31	0.31	0.31	0.36	0.65	-
2004-2006	0.46	0.79	0.91	0.68	0.68	0.40	0.89	0.77	0.31	0.31	0.40	0.46	0.79	0.79

Table S10. Post-hoc test *P* values of Coastal fraction in NEAC for *reduced* WGS dataset split per birth year classes. The significant *P* values are marked in red. *P* values trending towards significance marked in brown.

**Year classes**	1895-1897	1898-1900	1901-1903	1904-1906	1967-1969	1990-1992	1998-2000	2001-2003
1898-1900	0.6535	-	-	-	-	-	-	-
1901-1903	0.1681	0.6329	-	-	-	-	-	-
1904-1906	0.9190	0.8905	0.6329	-	-	-	-	-
1967-1969	0.9590	0.6329	0.1384	0.9190	-	-	-	-
1990-1992	0.6329	0.9590	0.3189	0.7176	0.6329	-	-	-
1998-2000	0.6329	0.9190	0.6329	0.8498	0.6329	0.9190	-	-
2001-2003	0.0069	0.1258	0.6535	0.4442	0.0069	0.0069	0.2255	-
2004-2006	0.4442	0.9035	0.5645	0.6535	0.4164	0.6535	0.9190	0.0527

Table S11. Pairwise comparisons inversion genotype proportions at LG01, LG02, LG07 and LG12 for NEAC and NCC. Table showing *P* value (Overall) from Fischer´s exact test. Significance level (Limit) given with Bonferroni correction, as well as limit Significant pairwise differences after Bonferroni correction in bold red and marked with *. Almost significant comparisons marked in bold brown.

LG01 NEAC	***P***	***Overall P*** / ***Limit***	2.02E-05 / 0.00178571
1907-1940	0.683	NS	
1907-1958	0.1599	NS	
1907-1975	0.8479	NS	
1907-1999	0.8157	NS	
**1907-2011**	**0.001214**	*****	
1907-2012	0.4255	NS	
1907-2014	0.1402	NS	
1940-1958	0.4878	NS	
1940-1975	0.4758	NS	
1940-1999	0.5932	NS	
**1940-2011**	**0.001443**	*****	
1940-2012	0.09105	NS	
1940-2014	0.1376	NS	
**1958-1975**	**0.03224**	**NS**	
1958-1999	0.1109	NS	
**1958-2011**	**2.28E-05**	*****	
**1958-2012**	**0.00786**	**NS**	
**1958-2014**	**0.005248**	**NS**	
1975-1999	1	NS	
**1975-2011**	**0.04186**	**NS**	
1975-2012	0.595	NS	
1975-2014	0.553	NS	
1999-2011	0.1636	NS	
1999-2012	0.4813	NS	
1999-2014	0.7673	NS	
**2011-2012**	**0.001251**	*****	
2011-2014	0.08938	NS	
2012-2014	0.06593	NS	
LG01 NCC	***P***	***Overall p*** / ***Limit***	0.1783 / 0.05
LG02 NEAC	***P***	***Overall p*** / ***Limit***	0.06563 / 0.05
LG02 NCC	***P***	***Overall p*** / ***Limit***	0.07493 / 0.05
LG07 NEAC	***P***	***Overall p*** / ***Limit***	0.3081 / 0.05
LG07 NCC	***P***	***Overall p*** / ***Limit***	0.3317 / 0.05
LG12 NEAC	***P***	***Overall p*** / ***Limit***	0.7564 / 0.05
LG12 NCC	***P***	***Overall p*** / ***Limit***	0.2183 / 0.05

Table S12. Chi-square table for inversion genotypes at LG01. Table displaying Genotype counts (O), genotype and allele frequencies (Geno freq and Allele freq), expected genotype frequencies and counts (E, under Hardy-Weinberg-Equilibrium), expected genotype counts as well as the test, degrees of freedom (df) and test statistic χ^2 and resulting *P* value (*P*). Genotypes abbreviated as DERIVED, HET (heterozygote) and ANCESTRAL.

LG01 *1907 NEAC*	*Count (O)*	*Geno freq*	*Allele freq*	*Expected Geno freq (HWE)*	*Expected Geno counts (E)*	*(O-E)^2*	*(O-E)^2 /E*		
**DERIVED**	22	0.81	0.87	0.76	20	2	0.12		
**HET**	3	0.11		0.23	6	10	1.57		
**ANCESTRAL**	2	0.07	0.13	0.02	0	2	5.27	***df***	1
**Tot**	**27**	**1**	**1**	**1**	**27**	χ^2	**6.96**	***P***	**0.008**
1940 NEAC									
**DERIVED**	19	0.90	0.95	0.91	19	0	0.00		
**HET**	2	0.10		0.09	2	0	0.00		
**ANCESTRAL**	0	0.00	0.05	0.00	0	0	0.05	***df***	1
**Tot**	**21**	**1**	**1**	**1**	**21**	χ^2	**0.05**	***P***	**0.819**
1958 NEAC									
**DERIVED**	21	1.00	1.00	1.00	21	0	0.00		
**HET**	0	0.00		0.00	0	0	0.00		
**ANCESTRAL**	0	0.00	0.00	0.00	0	0	0.00	***df***	1
**Tot**	**21**	**1**	**1**	**1**	**21**	χ^2	**0.00**	***P***	**1.00**
1975 NEAC									
**DERIVED**	13	0.76	0.85	0.73	12	0	0.03		
**HET**	3	0.18		0.25	4	2	0.38		
**ANCESTRAL**	1	0.06	0.15	0.02	0	0	1.09	***df***	1
**Tot**	**17**	**1**	**1**	**1**	**17**	χ^2	**1.50**	***P***	**0.22**
1999 NEAC									
**DERIVED**	9	0.82	0.91	0.83	9	0	0.00		
**HET**	2	0.18		0.17	2	0	0.02		
**ANCESTRAL**	0	0.00	0.09	0.01	0	0	0.09	***df***	1
**Tot**	**11**	**1**	**1**	**1**	**11**	χ^2	**0.11**	***P***	**0.74**
2011 NEAC									
**DERIVED**	23	0.48	0.73	0.53	26	6	0.25		
**HET**	24	0.50		0.39	19	25	1.34		
**ANCESTRAL**	1	0.02	0.27	0.07	4	6	1.80	***df***	1
**Tot**	**48**	**1**	**1**	**1**	**48**	χ^2	**3.39**	***P***	**0.07**
2012 NEAC									
**DERIVED**	23	0.66	0.74	0.55	19	14	0.70		
**HET**	6	0.17		0.38	13	54	4.06		
**ANCESTRAL**	6	0.17	0.26	0.07	2	14	5.87	***df***	1
**Tot**	**35**	**1**	**1**	**1**	**35**	χ^2	**10.64**	***P***	**0.001**
2014 NEAC									
**DERIVED**	28	0.68	0.83	0.69	28	0	0.00		
**HET**	12	0.29		0.28	12	0	0.01		
**ANCESTRAL**	1	0.02	0.17	0.03	1	0	0.03	***df***	1
**Tot**	**41**	**1**	**1**	**1**	**41**	χ^2	**0.05**	***P***	**0.830**
2011 NCC									
**DERIVED**	0	0.00	0.07	0.01	0	0	0.26		
**HET**	7	0.15		0.14	6	0	0.04		
**ANCESTRAL**	41	0.85	0.93	0.86	41	0	0.00	***df***	1
**Tot**	**48**	**1**	**1**	**1**	**48**	χ^2	**0.30**	***P***	**0.59**
2014 NCC									
**DERIVED**	0	0.00	0.03	0.00	0	0	0.03		
**HET**	2	0.05		0.05	2	0	0.00		
**ANCESTRAL**	37	0.95	0.97	0.95	37	0	0.00	***df***	1
**Tot**	**39**	**1**	**1**	**1**	**39**	χ^2	**0.03**	***P***	**0.87**

Table S13. Chi-square table for inversion genotypes at LG02. Table displaying Genotype counts (O), genotype and allele frequencies (Geno freq and Allele freq), expected genotype frequencies and counts (E, under Hardy-Weinberg-Equilibrium), expected genotype counts as well as the test, degrees of freedom (df) and test statistic χ^2 and resulting *P* value (*P*). Genotypes abbreviated as ANCESTRAL, HET (heterozygote) and DERIVED.

LG02 1907 NEAC	*Count (O)*	*Geno freq*	*Allele freq*	*Expected Geno freq (HWE)*	*Expected Geno counts (E)*	*(O-E)^2*	*(O-E)^2 /E*		
**ANCESTRAL**	24	0.89	0.94	0.89	24	0	0.00		
**HET**	3	0.11		0.10	3	0	0.01		
**DERIVED**	0	0.00	0.06	0.00	0	0	0.08	*df*	1
**Tot**	**27**	**1**	**1**	**1**	**27**	χ^2	**0.09**	***P***	**0.760**
1940 NEAC									
**ANCESTRAL**	21	1.00	1.00	1.00	21	0	0.00		
**HET**	0	0.00		0.00	0	0	0.00		
**DERIVED**	0	0.00	0.00	0.00	0	0	0.00	*df*	1
**Tot**	**21**	**1**	**1**	**1**	**21**	χ^2	**0.00**	***P***	**1.000**
1958 NEAC									
**ANCESTRAL**	21	1.00	1.00	1.00	21	0	0.00		
**HET**	0	0.00		0.00	0	0	0.00		
**DERIVED**	0	0.00	0.00	0.00	0	0	0.00	*df*	1
**Tot**	**21**	**1**	**1**	**1**	**21**	χ^2	**0.00**	***P***	**1.000**
1975 NEAC									
**ANCESTRAL**	16	0.94	0.97	0.94	16	0	0.00		
**HET**	1	0.06		0.06	1	0	0.00		
**DERIVED**	0	0.00	0.03	0.00	0	0	0.01	*df*	1
**Tot**	**17**	**1**	**1**	**1**	**17**	χ^2	**0.02**	***P***	**0.901**
1999 NEAC									
**ANCESTRAL**	11	1.00	1.00	1.00	11	0	0.00		
**HET**	0	0.00		0.00	0	0	0.00		
**DERIVED**	0	0.00	0.00	0.00	0	0	0.00	*df*	1
**Tot**	**11**	**1**	**1**	**1**	**11**	χ^2	**0.00**	***P***	**1.000**
2011 NEAC									
**ANCESTRAL**	47	0.98	0.99	0.98	47	0	0.00		
**HET**	1	0.02		0.02	1	0	0.00		
**DERIVED**	0	0.00	0.01	0.00	0	0	0.01	*df*	1
**Tot**	**48**	**1**	**1**	**1**	**48**	χ^2	**0.01**	***P***	**0.942**
2012 NEAC									
**ANCESTRAL**	27	0.77	0.84	0.71	25	5	0.18		
**HET**	5	0.14		0.26	9	18	1.97		
**DERIVED**	3	0.09	0.16	0.02	1	5	5.28	*df*	1
**Tot**	**35**	**1**	**1**	**1**	**35**	χ^2	**7.43**	***P***	**0.006**
2014 NEAC									
**ANCESTRAL**	37	0.90	0.95	0.90	37	0	0.00		
**HET**	4	0.10		0.09	4	0	0.01		
**DERIVED**	0	0.00	0.05	0.00	0	0	0.10	*df*	1
**Tot**	**41**	**1**	**1**	**1**	**41**	χ^2	**0.11**	***P***	**0.743**
2011 NCC									
**ANCESTRAL**	9	0.19	0.41	0.17	8	1	0.15		
**HET**	21	0.44		0.48	23	5	0.20		
**DERIVED**	18	0.38	0.59	0.35	17	1	0.07	*df*	1
**Tot**	**48**	**1**	**1**	**1**	**48**	χ^2	**0.42**	***P***	**0.519**
2014 NCC									
**ANCESTRAL**	3	0.08	0.42	0.18	7	14	2.07		
**HET**	26	0.68		0.49	19	56	3.01		
**DERIVED**	9	0.24	0.58	0.34	13	14	1.10	*df*	1
**Tot**	**38**	**1**	**1**	**1**	**38**	χ^2	**6.18**	***P***	**0.013**

Table S14. Chi-square table for inversion genotypes at LG07. Table displaying Genotype counts (O), genotype and allele frequencies (Geno freq and Allele freq), expected genotype frequencies and counts (E, under Hardy-Weinberg-Equilibrium), expected genotype counts as well as the test, degrees of freedom (df) and test statistic χ^2 and resulting P value (P). Genotypes abbreviated as DERIVED, HET (heterozygote) and ANCESTRAL.

LG07 1907 NEAC	*Count (O)*	*Geno freq*	*Allele freq*	*Expected Geno freq (HWE)*	*Expected Geno counts (E)*	*(O-E)^2*	*(O-E)^2 /E*		
**DERIVED**	26	0.96	0.98	0.96	26	0	0.00		
**HET**	1	0.04		0.04	1	0	0.00		
**ANCESTRAL**	0	0.00	0.02	0.00	0	0	0.01	*df*	1
**Tot**	**27**	**1**	**1**	**1**	**27**	χ^2	**0.01**	***P***	**0.922**
1940 NEAC									
**DERIVED**	20	0.95	1.00	0.95	20	0	0.00		
**HET**	1	0.05		0.05	1	0	0.00		
**ANCESTRAL**	0	0.00	0.00	0.00	0	0	0.00	*df*	1
**Tot**	**21**	**1**	**1**	**1**	**21**	χ^2	**0.00**	***P***	**0.998**
1958 NEAC									
**DERIVED**	21	1.00	1.00	1.00	21	0	0.00		
**HET**	0	0.00		0.00	0	0	0.00		
**ANCESTRAL**	0	0.00	0.00	0.00	0	0	0.00	*df*	1
**Tot**	**21**	**1**	**1**	**1**	**21**	χ^2	**0.00**	***P***	**1.000**
1975 NEAC									
**DERIVED**	16	0.94	0.97	0.94	16	0	0.00		
**HET**	1	0.06		0.06	1	0	0.00		
**ANCESTRAL**	0	0.00	0.03	0.00	0	0	0.00	*df*	1
**Tot**	**17**	**1**	**1**	**1**	**17**	χ^2	**0.00**	***P***	**0.997**
1999 NEAC									
**DERIVED**	11	1.00	1.00	1.00	11	0	0.00		
**HET**	0	0.00		0.00	0	0	0.00		
**ANCESTRAL**	0	0.00	0.00	0.00	0	0	0.00	*df*	1
**Tot**	**11**	**1**	**1**	**1**	**11**	χ^2	**0.00**	***P***	**1.000**
2011 NEAC									
**DERIVED**	41	0.85	0.93	0.86	41	0	0.00		
**HET**	7	0.15		0.14	6	0	0.04		
**ANCESTRAL**	0	0.00	0.07	0.01	0	0	0.26	*df*	1
**Tot**	**48**	**1**	**1**	**1**	**48**	χ^2	**0.30**	***P***	**0.586**
2012 NEAC									
**DERIVED**	30	0.86	0.91	0.84	29	1	0.02		
**HET**	4	0.11		0.16	5	2	0.40		
**ANCESTRAL**	1	0.03	0.09	0.01	0	1	2.15	*df*	1
**Tot**	**35**	**1**	**1**	**1**	**35**	χ^2	**2.57**	***p***	**0.109**
2014 NEAC									
**DERIVED**	40	0.98	0.99	0.98	40	0	0.00		
**HET**	1	0.02		0.02	1	0	0.00		
**ANCESTRAL**	0	0.00	0.01	0.00	0	0	0.01	*df*	1
**Tot**	**41**	**1**	**1**	**1**	**41**	χ^2	**0.01**	***P***	**0.937**
2011 NCC									
**DERIVED**	4	0.08	0.29	0.09	4	0	0.00		
**HET**	20	0.42		0.41	20	0	0.00		
**ANCESTRAL**	24	0.50	0.71	0.50	24	0	0.00	*df*	1
**Tot**	**48**	**1**	**1**	**1**	**48**	χ^2	**0.00**	***P***	**0.954**
2014 NCC									
**DERIVED**	3	0.08	0.37	0.14	5	5	0.90		
**HET**	22	0.58		0.47	18	19	1.05		
**ANCESTRAL**	13	0.34	0.63	0.40	15	5	0.31	*df*	1
**Tot**	**38**	**1**	**1**	**1**	**38**	χ^2	**2.26**	***P***	**0.132**

Table S15. Chi-square table for inversion genotypes at LG12. Table displaying Genotype counts (O), genotype and allele frequencies (Geno freq and Allele freq), expected genotype frequencies and counts (E, under Hardy-Weinberg-Equilibrium), expected genotype counts as well as the test, degrees of freedom (df) and test statistic χ^2 and resulting *P* value (*P*). Genotypes abbreviated as ANCESTRAL, HET (heterozygote) and DERIVED.

LG12<br>*1907 NEAC*	*Count (O)*	*Geno freq*	*Allele freq*	*Expected Geno freq (E)*	*Expected Geno counts (E)*	*(O-E)^2*	*(O-E)^2 /E*		
**ANCESTRAL**	26	0.96	0.98	0.96	26	0	0.00		
**HET**	1	0.04		0.04	1	0	0.00		
**DERIVED**	0	0.00	0.02	0.00	0	0	0.01	***df***	1
**Tot**	**27**	**1**	**1**	**1**	**27**	χ^2	**0.01**	***P***	**0.922**
1940 NEAC									
**ANCESTRAL**	21	1.00	1.00	1.00	21	0	0.00		
**HET**	0	0.00		0.00	0	0	0.00		
**DERIVED**	0	0.00	0.00	0.00	0	0	0.00	***df***	1
**Tot**	**21**	**1**	**1**	**1**	**21**	χ^2	**0.00**	***P***	**1.000**
1958 NEAC									
**ANCESTRAL**	21	1.00	1.00	1.00	21	0	0.00		
**HET**	0	0.00		0.00	0	0	0.00		
**DERIVED**	0	0.00	0.00	0.00	0	0	0.00	***df***	1
**Tot**	**21**	**1**	**1**	**1**	**21**	χ^2	**0.00**	***P***	**1.000**
1975 NEAC									
**ANCESTRAL**	17	1.00	1.00	1.00	17	0	0.00		
**HET**	0	0.00		0.00	0	0	0.00		
**DERIVED**	0	0.00	0.00	0.00	0	0	0.00	***df***	1
**Tot**	**17**	**1**	**1**	**1**	**17**	χ^2	**0.00**	***P***	**1.000**
1999 NEAC									
**ANCESTRAL**	11	1.00	1.00	1.00	11	0	0.00		
**HET**	0	0.00		0.00	0	0	0.00		
**DERIVED**	0	0.00	0.00	0.00	0	0	0.00	***df***	1
**Tot**	**11**	**1**	**1**	**1**	**11**	χ^2	**0.00**	***P***	**1.000**
2011 NEAC									
**ANCESTRAL**	47	0.98	0.99	0.98	47	0	0.00		
**HET**	1	0.02		0.02	1	0	0.00		
**DERIVED**	0	0.00	0.01	0.00	0	0	0.01	***df***	1
**Tot**	**48**	**1**	**1**	**1**	**48**	χ^2	**0.01**	***P***	**0.942**
2012 NEAC									
**ANCESTRAL**	31	0.89	0.93	0.86	30	1	0.02		
**HET**	3	0.09		0.13	5	3	0.58		
**DERIVED**	1	0.03	0.07	0.01	0	1	3.78	***df***	1
**Tot**	**35**	**1**	**1**	**1**	**35**	χ^2	**4.38**	***P***	**0.036**
2014 NEAC									
**ANCESTRAL**	39	0.95	0.98	0.95	39	0	0.00		
**HET**	2	0.05		0.05	2	0	0.00		
**DERIVED**	0	0.00	0.02	0.00	0	0	0.02	***df***	1
**Tot**	**41**	**1**	**1**	**1**	**41**	χ^2	**0.03**	***P***	**0.873**
2011 NCC									
**ANCESTRAL**	37	0.77	0.85	0.73	35	4	0.11		
**HET**	8	0.17		0.25	12	16	1.31		
**DERIVED**	3	0.06	0.15	0.02	1	4	3.84	***df***	1
**Tot**	**48**	**1**	**1**	**1**	**48**	χ^2	**5.26**	***P***	**0.022**
2014 NCC									
**ANCESTRAL**	27	0.71	0.86	0.73	28	1	0.02		
**HET**	11	0.29		0.25	9	3	0.27		
**DERIVED**	0	0.00	0.14	0.02	1	1	0.80	***df***	1
**Tot**	**38**	**1**	**1**	**1**	**38**	χ^2	**1.09**	***P***	**0.297**

Table S16. Comparisons genotype proportions at haemoglobin locus *hb* β*1* position 14570979 and 14570998 for NEAC and NCC. Table showing *P* value (Overall *P*) from Fischer´s exact test. Significance level (Limit) given with Bonferroni correction.

***hb* β*1* 14570979**	***NEAC***	***Overall P***	0.4633
		Limit	0.00178571
	NCC	***Overall P***	0.7725
		Limit	0.05
***hb* β*1* 14570998**	***NEAC***	***Overall P***	0.4757
		Limit	0.00178571
	NCC	***Overall P***	0.3336
		Limit	0.05

Table S17. Chi-square table for inversion genotypes at haemoglobin *hb* β*1* position 14570979. Table displaying Genotype counts (O), genotype and allele frequencies (Geno freq and Allele freq), expected genotype frequencies and counts (E, under Hardy-Weinberg-Equilibrium), expected genotype counts as well as the test, degrees of freedom (df) and test statistic χ^2 and resulting *P* value (*P*). Genotypes abbreviated as Val (Valine), HET (heterozygote) and Met (Methionine).

hb 14570979 1907 NEAC	*Count (O)*	*Geno freq*	*Allele freq*	*Expected Geno freq (HWE)*	*Expected Geno counts (E)*	*(O-E)^2*	*(O-E)^2 /E*		
**Val**	21	0.78	0.89	0.79	21	0	0.01		
**HET**	6	0.22		0.20	5	0	0.08		
**Met**	0	0.00	0.11	0.01	0	0	0.33	***df***	1
**Tot**	**27**	**1**	**1**	**1**	**27**	χ^2	**0.42**	***P***	**0.516**
1940 NEAC									
**Val**	17	0.89	0.95	0.90	17	0	0.00		
**HET**	2	0.11		0.10	2	0	0.01		
**Met**	0	0.00	0.05	0.00	0	0	0.05	***df***	1
**Tot**	**19**	**1**	**1**	**1**	**19**	χ^2	**0.06**	***P***	**0.809**
1958 NEAC									
**Val**	19	0.95	0.98	0.95	19	0	0.00		
**HET**	1	0.05		0.05	1	0	0.00		
**Met**	0	0.00	0.03	0.00	0	0	0.01	***df***	1
**Tot**	**20**	**1**	**1**	**1**	**20**	χ^2	**0.01**	***P***	**0.909**
1975 NEAC									
**Val**	15	1.00	1.00	1.00	15	0	0.00		
**HET**	0	0.00		0.00	0	0	0.00		
**Met**	0	0.00	0.00	0.00	0	0	0.00	***df***	1
**Tot**	**15**	**1**	**1**	**1**	**15**	χ^2	**0.00**	***P***	**1.000**
1999 NEAC									
**Val**	10	0.91	0.95	0.91	10	0	0.00		
**HET**	1	0.09		0.09	1	0	0.00		
**Met**	0	0.00	0.05	0.00	0	0	0.02	***df***	1
**Tot**	**11**	**1**	**1**	**1**	**11**	χ^2	**0.02**	***P***	**0.875**
2011 NEAC									
**Val**	20	0.91	0.95	0.91	20	0	0.00		
**HET**	2	0.09		0.09	2	0	0.00		
**Met**	0	0.00	0.05	0.00	0	0	0.05	***df***	1
**Tot**	**22**	**1**	**1**	**1**	**22**	χ^2	**0.05**	***P***	**0.823**
2014 NEAC									
**Val**	19	0.86	0.93	0.87	19	0	0.00		
**HET**	3	0.14		0.13	3	0	0.01		
**Met**	0	0.00	0.07	0.00	0	0	0.10	***df***	1
**Tot**	**22**	**1**	**1**	**1**	**22**	χ^2	**0.12**	***P***	**0.731**
2011 NCC									
**Val**	9	0.38	0.67	0.44	11	3	0.26		
**HET**	14	0.58		0.44	11	11	1.04		
**Met**	1	0.04	0.33	0.11	3	3	1.04	***df***	1
**Tot**	**24**	**1**	**1**	**1**	**24**	χ^2	**2.34**	***P***	**0.126**
2014 NCC									
**Val**	11	0.48	0.72	0.51	12	1	0.06		
**HET**	11	0.48		0.41	9	3	0.30		
**Met**	1	0.04	0.28	0.08	2	1	0.38	***df***	1
**Tot**	**23**	**1**	**1**	**1**	**23**	χ^2	**0.74**	***P***	**0.389**

Table S18. Chi-square table for inversion genotypes at haemoglobin *hb* β*1* position 14570998. Table displaying Genotype counts (O), genotype and allele frequencies (Geno freq and Allele freq), expected genotype frequencies and counts (E, under Hardy-Weinberg-Equilibrium), expected genotype counts as well as the test, degrees of freedom (df) and test statistic χ^2 and resulting *P* value (*P*). Genotypes abbreviated as Ala (Alanine), HET (heterozygote) and Lys (Lysine).

hb 14570998 1907 NEAC	*Count (O)*	*Geno freq*	*Allele freq*	*Expected Geno freq (HWE)*	*Expected Geno counts (E)*	*(O-E)^2*	*(O-E)^2 /E*		
**Ala**	21	0.78	0.89	0.79	21	0	0.01		
**HET**	6	0.22		0.20	5	0	0.08		
**Lys**	0	0.00	0.11	0.01	0	0	0.33	*df*	1
**Tot**	**27**	**1**	**1**	**1**	**27**	χ^2	**0.42**	***P***	**0.516**
1940 NEAC									
**Ala**	20	0.95	0.98	0.95	20	0	0.00		
**HET**	1	0.05		0.05	1	0	0.00		
**Lys**	0	0.00	0.02	0.00	0	0	0.01	*df*	1
**Tot**	**21**	**1**	**1**	**1**	**21**	χ^2	**0.01**	***P***	**0.911**
1958 NEAC									
**Ala**	20	0.95	0.98	0.95	20	0	0.00		
**HET**	1	0.05		0.05	1	0	0.00		
**Lys**	0	0.00	0.02	0.00	0	0	0.01	*df*	1
**Tot**	**21**	**1**	**1**	**1**	**21**	χ^2	**0.01**	***P***	**0.911**
1975 NEAC									
**Ala**	13	0.87	0.90	0.81	12	1	0.06		
**HET**	1	0.07		0.18	3	3	1.07		
**Lys**	1	0.07	0.10	0.01	0	1	4.82	*df*	1
**Tot**	**15**	**1**	**1**	**1**	**15**	χ^2	**5.95**	***P***	**0.015**
1999 NEAC									
**Ala**	10	0.91	0.95	0.91	10	0	0.00		
**HET**	1	0.09		0.09	1	0	0.00		
**Lys**	0	0.00	0.05	0.00	0	0	0.02	*df*	1
**Tot**	**11**	**1**	**1**	**1**	**11**	χ^2	**0.02**	***P***	**0.875**
2011 NEAC									
**Ala**	20	0.91	0.95	0.91	20	0	0.00		
**HET**	2	0.09		0.09	2	0	0.00		
**Lys**	0	0.00	0.05	0.00	0	0	0.05	*df*	1
**Tot**	**22**	**1**	**1**	**1**	**22**	χ^2	**0.05**	***P***	**0.823**
2014 NEAC									
**Ala**	19	0.86	0.93	0.87	19	0	0.00		
**HET**	3	0.14		0.13	3	0	0.01		
**Lys**	0	0.00	0.07	0.00	0	0	0.10	*df*	1
**Tot**	**22**	**1**	**1**	**1**	**22**	χ^2	**0.12**	***P***	**0.731**
2011 NCC									
**Ala**	9	0.38	0.63	0.39	9	0	0.02		
**HET**	12	0.50		0.47	11	1	0.05		
**Lys**	3	0.13	0.38	0.14	3	0	0.04	*df*	1
**Tot**	**24**	**1**	**1**	**1**	**24**	χ^2	**0.11**	***P***	**0.744**
2014 NCC									
**Ala**	9	0.39	0.70	0.48	11	5	0.41		
**HET**	14	0.61		0.42	10	18	1.86		
**Lys**	0	0.00	0.30	0.09	2	5	2.13	*df*	1
**Tot**	**23**	**1**	**1**	**1**	**23**	χ^2	**4.40**	***P***	**0.036**

Table S19. Result of the relationship exploration using GAM model. gam(proportion of different genotype ~ s(variable), weights=count of fish each year, quasibinomial, method="REML", scale=1). 'edf' is the estimated degrees of freedom of the smoothing term. A '–' indicates that no model was found. In bold are indicated the significant models $P<0.05$ and underlined when $P<0.10$. qAIC is an estimated quasi-Akaike Information Criterion calculated on reduced time series (i.e., n=29) using the formula qAIC = model deviance/ĉ + 2 ·k · ĉ, with k the effective degrees of freedom, ĉ the dispersion parameter. Competitive models have a ΔqAIC ≤2, with ΔqAIC = qAIC - min(qAIC). A qAIC of '–' indicates that no model was found using the reduced time series.

$F_{5.10}$: Fishing mortality, μ: generation time, wNAO: winter North Atlantic Oscillation, Recruits: number of recruits, SSB: spawning stock biomass, and ST: Kola section sea temperature.

Variable			LG01						LG02			LG07			LG12			Val-Ala		
			DERIVED			ANCESTRAL			ANCESTRAL			DERIVED			ANCESTRAL					
		n	edf	R^2	qAIC	edf	R^2	qAIC	edf	R^2	qAIC	edf	R^2	qAIC	edf	R^2	qAIC	edf	R^2	qAIC
$F_{5.10}$		29	1	**0.48**	31	1	**0.22**	29	1	**0.31**	29	1.34	0.26	29	1	**0.34**	28	–	–	–
μ		29	1	**0.35**	32	1	0.03	30	1	<u>0.13</u>	29	1	0.05	30	1	0.08	29	–	–	–
mean wNAO	before	38	–	–	–	1	0.03	–	1	0.01	–	–	–	–	1.72	0.03	29	–	–	–
max wNAO	before	38	1.74	<u>0.07</u>	36	1.05	<u>0.12</u>	30	1	**0.37**	30	–	–	–	1.17	0.01	28	1.83	0.14	–
mean wNAO	after	38	–	–	–	–	–	–	–	–	–	–	–	–	–	–	–	–	–	–
max wNAO	after	38	1.82	0.04	37	1.6	**0.10**	30	1.28	<u>0.19</u>	30	–	–	–	1	0.09	29	1.84	**0.36**	–
Recruits		29	–	–	–	–	–	–	1	0.01	30	–	–	–	1	0.03	29	1	0.12	–
SSB		33	1.84	**0.10**	36	1	<u>0.03</u>	30	1.71	0.03	–	1.6	0.11	30	1.72	0.02	29	1.77	0.07	–
mean ST	before	38	1	**0.08**	34	1.67	0.03	–	1.66	0.04	30	–	–	–	1	0.01	29	–	–	–
max ST	before	38	1.47	**0.09**	34	1.85	<u>0.15</u>	31	1.83	<u>0.18</u>	30	–	–	–	–	–	–	1.19	0.08	–
mean ST	after	38	1.29	**0.24**	33	1.54	0.01	30	1.72	0.18	29	–	–	–	1.09	0.10	28	–	–	–
max ST	after	38	1.43	**0.11**	34	–	–	–	1.57	0.05	30	–	–	–	1.23	0.04	29	1.46	0.05	–

Table S20. F_{ST} outliers in common between 3 or more comparisons. Table showing F_{ST} outlier window with chromosome and window span, number of comparisons in common, which comparisons that are common, the genetic element corresponding to AFD quantile or F_{ST} window as from gadMor2 annotated and filtered gff-file, with minor conversions of gene names. Positions of corresponding AFD and $_{XP}$EHH outlier if present, as well as comments regarding matches.

CHROM	START	STOP	COMP	COMMON BETWEEN	GENETIC ELEMENT	AFD	COMMENT	POS $_{XP}$EHH	COMMENT
LG04	10050001	10080000	3	1907-2011, 1999-2011, 2011-2014	*zeb2*: Zinc finger E-box-binding homeobox 2 (*Homo sapiens*)	10056163, 10055937	each site only in one comp	NA	
LG05	3420001	3450000	3	1907-2014, 1975-2014, 1999-2014	Uncharacterized protein U88	3448087, 3448172	3448087 in two AFD comps	NA	
LG05	3435001	3465000	3	1907-2014, 1975-2014, 1999-2014	U88_HHV6U Uncharacterized protein U88	3448087, 3448172	3448087 in two AFD comps	NA	
LG06	5310001	5340000	3	1907-1975, 1975-1999, 1975-2011	U88_HHV6U Uncharacterized protein U88	5324216	only one comp corresponds to outlier	NA	
LG12	6735001	6765000	3	1907-1975, 1975-2011, 1975-2014	sp|Q06666|T2_MOUSE	6735805	same position all AFD comps, inside inversion	6735805	only for two comps
LG13	21675001	21705000	4	1907-1999, 1975-1999, 1999-2011, 1999-2014	Immunoglobulin Superfamily Member 22 (*Homo sapiens*)	21701268, 21701294	21702294 common between 3 comps, 21701268 between 2	NA	
LG13	21690001	21720000	4	1907-1999, 1975-1999, 1999-2011, 1999-2014	Immunoglobulin Superfamily Member 22 (*Homo sapiens*)	21701268, 21701294	21702294 common between 3 comps, 21701268 between 2	NA	
LG15	14430001	14460000	3	1907-1975, 1975-2011, 1975-2014	Protocadherin-15 (*Gallus gallus*)	14441532, 14459829	14459829 common 3 comp, also close site found at 14427464 for 1 comp and within 30kb window for one comp 14441532	NA	
LG17	4575001	4605000	3	1975-2014, 1999-2011, 1999-2014	*tkfc*: Triokinase/FMN cyclase (*Rattus norvegicus*) and *cyp26b1*: Cytochrome P450 26B1 (*Danio rerio*)	NA	Not common AFD and $_{XP}$EHH	NA	

Table S20 continued on next page

Table S20 continued from previous page

CHROM	START	STOP	COMP	COMMON BETWEEN	GENETIC ELEMENT	AFD	COMMENT	POS $_{XP}$EHH	COMMENT
LG22	17925001	17955000	3	1975-2011, 1975-1999, 1975-2014	*cdca8*: Borealin (*Danio rerio*)	17942454, 17942483	sites in 2 comps	NA	
LG22	17940001	17970000	3	1975-2011, 1975-1999, 1975-2014	*cdca8*: Borealin (*Danio rerio*)	17942454, 17942483	sites in 2 comps	NA	
LG22	9435001	9465000	3	1975-2011, 1975-1999, 1975-2014	sp|Q6ZRP5|YD019_HUMAN, Uncharacterized protein KIAA1522 homolog (*Danio rerio*)	9435078, 9558423	9435078 common between all 3, 9558423 between 2	NA	closest at 9557038 for 1 comp and 9557247 for 2 comps
LG23	5865001	5895000	3	1907-1975, 1975-1999, 1975-2011	*tfrc*: Transferrin receptor 1 (*Pongo abelii*)	5890304, 5890539	5890539 between 3 comps	NA	
LG23	5880001	5910000	3	1907-1975, 1975-1999, 1975-2011	*tfrc*: Transferrin receptor 1 (*Pongo abelii*)	5890304, 5890539	5890539 between 3 comps	NA	

Table S21. F_{ST} outliers in common between 2 comparisons. Table showing F_{ST} outlier window with chromosome and window span, number of comparisons in common, which comparisons that are common, the genetic element corresponding to AFD quantile or F_{ST} window as from gadMor2 annotated and filtered gff-file, with minor conversions of gene names. Positions of corresponding AFD and $_{XP}$EHH outlier if present, as well as comments regarding matches.

CHROM	START	STOP	COMP	COMMON BETWEEN	GENETIC ELEMENT	AFD	COMMENT	POS $_{XP}$EHH	COMMENT
LG01	300001	330000	2	1975-2014, 1999-2014	expressed_sequence_match, c3660/f1p0/1589	NA	NA	313291	313291 only 1 comp
LG01	3390001	3420000	2	1907-1975, 1975-1999	*dnah1*: Dynein heavy chain 1, axonemal (*Rattus norvegicus*)	NA	NA	NA	closest match at 3483483
LG01	4395001	4425000	2	1907-1975, 1975-1999	augustus_masked-LG01-abinit-gene-44.0-mRNA-1	NA	NA	NA	NA
LG01	4410001	4440000	2	1907-1975, 1975-1999	*spryd3*: SPRY domain-containing protein 3 (*Homo sapiens*)	NA	NA	NA	closest match at 4452909
LG03	8610001	8640000	2	1975-2011, 2011-2014	*suclg1*: Succinyl-CoA ligase subunit alpha, mitochondrial (Fragment) (*Columba livia*)	8615363	AFD only for 1 comp	NA	closest match at 8586151
LG03	22140001	22170000	2	1999-2011, 1999-2014	No match	22147214, 22160122	both sites in 2 comps	NA	close matches at 22125593 and 22132586
LG04	13140001	13170000	2	1907-2014, 2011-2014	*slc2a9:* Solute carrier family 2, facilitated glucose transporter member 9 *(Pongo abelii)*	13144189	AFD for 1 comp	NA	NA
LG05	10080001	10110000	2	1907-1975, 1975-1999	*lin52*: Protein lin-52 homolog (*Oncorhynchus mykiss*)	10103621	10103621 for 2 comps	NA	NA
LG05	10095001	10125000	2	1907-1975, 1975-1999	*lin52*: Protein lin-52 homolog (*Oncorhynchus mykiss*)	10103621	10103621 for 2 comps	NA	NA
LG06	6 480 001	6510000	2	1975-1999, 1975-2011	*rab11fip1*: Rab11 family-interacting protein 1 (*Mus musculus*)	NA	NA	6501233	6501233 only 1 comp
LG06	14730001	14760000	2	1907-2014, 1975-2014	*edil3*: EGF-like repeat and discoidin I-like domain-containing protein 3 (*Homo sapiens*)	14751288	14751288 for both comps	NA	NA
LG06	14745001	14775000	2	1907-2014, 1975-2014	*edil3*: EGF-like repeat and discoidin I-like domain-containing protein 3 (*Homo sapiens*)	14751288	14751288 for both comps	NA	NA

Table S21 continued on next page

Table S21 continued from previous page

CHROM	START	STOP	COMP	COMMON BETWEEN	GENETIC ELEMENT	AFD	COMMENT	POS $_{XP}$EHH	COMMENT
LG06	17730001	17760000	2	1907-2011, 1999-2011	sp|Q6ZRP5|YD019_HUMAN	17732471	17732471 only 1 comp, 1 site outside for other comp 17761253	NA	closest matches 17720068 and 17720212
LG07	14760001	14790000	2	1907-1975, 1975-1999	sp|Q6ZRP5|YD019_HUMAN	14772935	14772935 for 1 comp	NA	close match at 14752338
LG07	15375001	15405000	2	1975-1999, 1975-2011	sp|Q69566|U88_HHV6U	15383178	15383178 for both comps	NA	NA
LG07	15630001	15660000	2	1907-2011, 2011-2014	sp|Q69566|U88_HHV6U	15647412	15647412 only 1 comp	NA	closest match at 15707214
LG07	20520001	20550000	2	1999-2014, 2011-2014	*vps37d*: Vacuolar protein sorting-associated protein 37D (*Mus musculus*)	20537448, 20536772	AFD different between 2 comps	NA	NA
LG08	9255001	9285000	2	1907-2014, 1975-2014	snap_masked-LG08-abinit-gene-93.52-mRNA-1	9276360, 9278668	9276360 for both comps	NA	NA
LG08	13005001	13035000	2	1907-2011, 1999-2011	augustus_masked-LG08-abinit-gene-130.36-mRNA-1	NA	NA	NA	NA
LG08	26490001	26520000	2	1907-1975, 1975-2011	*kcnh2*: Potassium voltage-gated channel subfamily H member 2 (*Homo sapiens*)	26493564	26493564 only 1 comp	NA	closest match at 26475841, 26475958 and 26342881, 26342885
LG09	5055001	5085000	2	1999-2011, 2011-2014	*sema4b*: Semaphorin-4B (*Mus musculus*)	NA	NA	NA	NA
LG09	5070001	5100000	2	1907-1999, 1907-2014	*sema4b*: Semaphorin-4B (*Mus musculus*)	5089420	5089420 only 1 comp, inside codon for F	NA	closest match closest 5112279
LG09	16800001	16830000	2	1975-2011, 1975-2014	snap_masked-LG09-abinit-gene-168.94-mRNA-1	NA	NA	NA	close match at 16832411 1 comp
LG11	7335001	7365000	2	1907-1999, 1975-1999	genemark-LG11-abinit-gene-73.67-mRNA-1	NA	NA	NA	NA
LG11	21915001	21945000	2	1999-2011, 1999-2014	*tbc1* domain family member 31 (*Xenopus tropicalis*)	21928585, 21934748	21934748 for 2 comps	NA	NA
LG12	2925001	2955000	2	1907-1999, 1999-2014	genemark-LG12-abinit-gene-30.5-mRNA-1	2941425	2941425 for 2 comps	NA	NA

Table S21 continued on next page

Table S21 continued from previous page

CHROM	START	STOP	COMP	COMMON BETWEEN	GENETIC ELEMENT	AFD	COMMENT	POS xpEHH	COMMENT
LG12	7725001	7755000	2	1907-1999, 1975-1999	*pex5l*: PEX5-related protein (*Homo sapiens*)	NA	NA	NA	NA
LG12	4395001	4425000	2	1999-2011, 1999-2014	sp|Q8JFR5|KITA_DANRE	4417627	4417627 only for 1 comp	4397045, 4397102, 4397197	all 3 only for 1 comp
LG13	3765001	3795000	2	1907-1975, 1975-2014	Vitamin D3 receptor B (*Danio rerio*)	3773664	3773664 only 1 comp	NA	NA
LG13	8295001	8325000	2	1975-2011, 1975-2014	No match	8322002	8322002 for 2 comps	NA	closest match at 8204402 or 8038391
LG14	2820001	2850000	2	1907-1999, 1999-2011	sp|Q69566|U88_HHV6U	NA	NA	NA	NA
LG14	7635001	7665000	2	1907-1975, 1975-2011	*mrpl46*: 39S ribosomal protein L46, mitochondrial (*Bos taurus*)	7642083, 7642090	both sites in 2 comps	NA	NA
LG14	9870001	9900000	2	1907-2014, 2011_2014	sp|Q6ZRP5|YD019_HUMAN	9888810	9888810 between 2 comps	9888231, 9888721, 9888728	only 1 comp
LG14	27975001	28005000	2	1907-1975, 1975-2011	genemark-LG14-abinit-gene-280.16-mRNA-1	NA	NA	NA	NA
LG15	14415001	14445000	2	1907-1975, 1975-2011	*pcdh15*: Protocadherin-15 (*Gallus gallus*)	14427464, 14441532	1 site for each comp	NA	NA
LG16	4665001	4695000	2	1907-2011, 2011-2014	mRNA, GAMO_00008680-RA, *tsg101*: Tumour susceptibility gene 101 protein (*Homo sapiens*)	4687372	4687372 between 2 comps	NA	NA
LG16	4680001	4710000	2	1907-2011, 2011-2014	mRNA, GAMO_00008680-RA, *tsg101*: Tumour susceptibility gene 101 protein (*Homo sapiens*)	4687372	4687372 between 2 comps	NA	NA
LG16	8175001	8205000	2	1975-2011, 1999-2011	*fat3*: Protocadherin Fat 3 (*Homo sapiens*)	8192469	8192469 only 1 comp	NA	NA
LG17	8490001	8520000	2	1975-1999, 1975-2011	*rbm4*: RNA-binding protein 4 (*Bos taurus*)	8517172	8517172 only 1 comp	NA	NA
LG17	8505001	8535000	2	1975-1999, 1975-2011	*rbm4*: RNA-binding protein 4 (*Bos taurus*)	8517172	8517172 only 1 comp	NA	NA

Table S21 continued on next page

Table S21 continued from previous page

CHROM	START	STOP	COMP	COMMON BETWEEN	GENETIC ELEMENT	AFD	COMMENT	POS $_{XP}$EHH	COMMENT
LG17	8550001	8580000	2	1907-1975, 1975-2011	*tmem88*: Transmembrane protein 88 (*Mus musculus*)	8577844, 8578155	8577844 and 8578155 1 comp, close 8517172 and 8623226 for another comp	NA	NA
LG17	8565001	8595000	2	1907-1975, 1975-2011	*tmem88*: Transmembrane protein 88 (*Mus musculus*)	8577844, 8578155	8577844 and 8578155 1 comp, close 8517172 and 8623226 for the other comp	NA	NA
LG17	9360001	9390000	2	1907-2011, 1999-2011	augustus_masked-LG17-abinit-gene-93.2-mRNA-1	9360892	9360892 for 1 comp, the other comp closest 9402153	NA	NA
LG18	9930001	9960000	2	1907-1999, 1999-2011	genemark-LG18-abinit-gene-99.35-mRNA-1	NA	NA	9930047, 9930221	only 1 comp
LG18	10260001	10290000	2	1907-2011, 1907-2014	sp|Q6ZRP5|YD019_HUMAN	NA	NA	NA	NA
LG18	12015001	12045000	2	1907-1999, 1999-2011	*ppa1*: Inorganic pyrophosphatase (*Bos taurus*)	NA	NA	NA	NA
LG19	3465001	3495000	2	1907-1975, 1975-1999	LG19: hit: 72394:3.2.0.35	NA	NA	NA	closest matches at close at 3536698 and 3536923
LG19	6480001	6510000	2	1907-1975, 1975-2014	LG19: hit: 76812:3.2.0.65 (expressed seq match)	6501269, 6501271	both sites in 2 comps	NA	NA
LG20	8130001	8160000	2	1907-1975, 1975-2014	E3 ubiquitin-protein ligase *mycbp2* (*Homo sapiens*)	8141673	8141673 for 2 comps	NA	NA
LG20	13680001	13710000	2	1907-1975, 1975-2014	No match	13703614	13703614 only 1 comp	NA	NA
LG20	19020001	19050000	2	1907-1975, 1975-2014	snap_masked-LG20-abinit-gene-190.64-mRNA-1	19046571	19046571 only 1 comp	NA	NA
LG20	19035001	19065000	2	1907-1975, 1975-2014	snap_masked-LG20-abinit-gene-190.64-mRNA-1	19046571, 19072071	19046571 and 19072071 only 1 comp	NA	NA
LG21	6510001	6540000	2	1999-2011, 2011-2014	sp|Q6NY98|MCE1_DANRE	6525827	6525827 only for 1 comp	NA	closest match at Closest at 6555512

Table S21 continued on next page

Table S21 continued from previous page

CHROM	START	STOP	COMP	COMMON BETWEEN	GENETIC ELEMENT	AFD	COMMENT	POS $_{XP}$EHH	COMMENT
LG22	615001	645000	2	1907-1975, 1975-1999	*pex1*: Peroxisome biogenesis factor 1 (Homo sapiens)	634584	634584 for 2 comps	634584	634584 for both comp
LG22	1515001	1545000	2	1999-2011, 2011-2014	*atp6v1c1a*: V-type proton ATPase subunit C 1-A (*Danio rerio*)	NA	NA	NA	NA
LG22	8055001	8085000	2	1975-2011, 1975-2014	No match	8056273	8056273 in 2 comps	NA	NA
LG22	9420001	9450000	2	1975-1999, 1975-2014	sp|Q6ZRP5|YD019_HUMAN	9435078	9435078 in 2 comps	NA	NA
LG23	1935001	1965000	2	1975-2011, 1999-2011	augustus_masked-LG23-abinit-gene-19.9-mRNA-1	NA	NA	NA	NA
LG23	2970001	3000000	2	1999-2011, 1999-2014	*clec10a*: C-type lectin domain family 10 member A (*Homo sapiens*)	NA	NA	NA	NA

Table S22. BayPass outlier statistics per LG. Number of outlier sites with BF > 20 and passing simulation threshold for LG01-LG23 per variable, followed by summary statistics in the form of total number of outlier sites, average number of sites per LG, standard deviation of number of sites per LG.

	mean ST 3 years before birth	max ST 3 years before birth	ST year of birth	mean ST 3 years after birth	max ST 3 years after birth	max wNAO 3 years before birth	wNAO year of birth	max wNAO 3 years after birth	F5.10 year of birth	Genera-tion time
**LG01**	0	0	0	2	1	0	0	1	3	1
**LG02**	0	1	1	2	2	0	0	0	5	6
**LG03**	0	0	0	0	0	2	1	2	6	2
**LG04**	1	2	1	0	2	0	1	0	5	3
**LG05**	0	1	0	0	0	0	1	0	4	3
**LG06**	2	1	1	1	1	0	0	2	2	1
**LG07**	0	1	4	1	2	0	0	0	4	1
**LG08**	1	0	0	1	0	1	0	0	2	2
**LG09**	1	2	2	2	1	0	0	1	3	6
**LG10**	0	0	0	1	0	0	0	0	5	2
**LG11**	0	1	3	1	1	0	0	2	2	2
**LG12**	0	0	1	0	2	2	1	1	2	3
**LG13**	1	1	1	1	1	1	0	0	1	1
**LG14**	1	1	2	3	1	0	1	0	4	1
**LG15**	0	0	0	1	0	0	0	0	3	7
**LG16**	0	1	1	0	2	0	0	1	4	4
**LG17**	0	0	0	0	0	2	1	0	7	5
**LG18**	0	0	0	0	0	0	0	0	4	4
**LG19**	1	0	1	0	0	0	2	1	1	0
**LG20**	0	0	0	0	0	0	1	0	1	3
**LG21**	0	0	0	0	0	0	1	0	2	1
**LG22**	1	1	1	0	0	0	2	1	1	1
**LG23**	1	0	0	0	0	1	0	1	1	1
**Total number of outliers**	10	13	19	16	16	9	12	13	72	60
**Number of LGs with outliers**	9	11	12	11	11	6	10	10	23	22
**Average number sites per LG**	0.4	0.6	0.8	0.7	0.7	0.4	0.5	0.6	3.1	2.6
**SD sites per LG**	0.6	0.7	1.1	0.9	0.8	0.7	0.7	0.7	1.7	1.9

Table S23. BayPass outlier statistics per LG at inversions. Number of outliers within inversions (LG01, LG02, LG07 and LG12) and fraction of outliers within inversions.

	mean ST 3 years before birth	max ST 3 years before birth	ST year of birth	mean ST 3 years after birth	max ST 3 years after birth	max wNAO 3 before birth	wNAO year of birth	max wNAO 3 years after birth	F5.10 year of birth	Genera-tion time
**LG01**	0	0	0	2	1	0	0	0	0	1
**LG02**	0	1	0	0	1	0	0	0	0	1
**LG07**	0	1	3	1	2	0	0	0	2	1
**LG12**	0	0	1	0	1	1	0	1	1	2
**Number of outliers within inversions**	0	2	4	3	5	1	0	1	3	5
**Fraction of outliers within inversions**	0.00	0.15	0.21	0.19	0.31	0.11	0.00	0.08	0,04	0.08

Table S24. BayPass hits within exons and UTRs. Table showing all hits within exons and UTRs, ordered by BayPass variable. $F_{5.10}$ (fishing mortality at year of birth), μ (generation time at year of birth), and different variables of ST and wNAO. CHROM= Chromosome, linkage group, POS=position, SNPID=Site id as linkage group:position (LG:position), HIT=hits described as in annotated gff, REF= Reference allele, if single letter denoting nucleotide, if three letters denoting codon. Within brackets denoting amino acid, and when opposite stand coding for amino acid original sequence is given second within brackets, ALT= Alternate allele, if single letter denoting nucleotide, if three letters denoting codon. Within brackets denoting amino acid, and when opposite stand coding for amino acid original sequence is given second.

VAR	CHROM	POS	SNPID	HIT	REF	ALT	COMMENT
F5.10	LG17	17162218	LG17:17162218	Exon, Similar to ADAMTSL1: ADAMTS-like protein 1 (*Homo sapiens*)	GAA (Glu)	GGG (Gly)	Non-synonymous, combo with following site
F5.10	LG17	17162219	LG17:17162219	Exon, Similar to ADAMTSL1: ADAMTS-like protein 1 (*Homo sapiens*)	GAA (Glu)	GGG (Gly)	Non-synonymous, combo with previous site
F5.10	LG14	8304041	LG14:8304041	Exon, Similar to Casein kinase II subunit alpha' (Gallus gallus)	A	G	No readframe
F5.10, μ	LG18	6755204	LG18:6755204	Exon, Similar to *kif19*: Kinesin-like protein KIF19 (*Xenopus laevis*)	ATT (Ile) (AAT)	ATC (Ile) (GAT)	Synonymous
wNAO	LG21	16811704	LG21:16811704	Exon, Similar to *otx2*: Homeobox protein OTX2 (*Danio rerio*)	TCA (Ser)	TCG (Ser)	Synonymous
mean ST 3years after birth	LG08	18121728	LG08:18121728	Exon, Similar to *sirt5*: NAD-dependent protein deacylase sirtuin-5, mitochondrial (*Xenopus tropicalis*)	T	C	No readframe
max wNAO 3 years after birth	LG01	2781710	LG01:2781710	Exon, Similar to *slc2a10*: Solute carrier family 2, facilitated glucose transporter member 10 (*Xenopus laevis*)	CAG (Gln) (CTG)	CGG (Arg) (CCG)	Non-synonymous
μ	LG20	21814300	LG20:21814300	Exon, Similar to *slitrk5*: SLIT and NTRK-like protein 5 (*Homo sapiens*)	TTA (Leu)	TTG (Leu)	Synonymous
F5.10	LG10	2204380	LG10:2204380	UTR, Similar to *dnd*: Dead end protein 1 (Danio rerio)	T	G	UTR
mean ST 3 years after birth	LG15	23964214	LG15:23964214	UTR, Similar to *scrib*: Protein scribble homolog (*Homo sapiens*)	T	G	UTR
mean ST 3 years after birth	LG14	22513110	LG14:22513110	UTR, Similar to *tmc3*: Transmembrane channel-like protein 3 (*Gallus gallus*)	T	C	UTR
max wNAO 3 years after birth	LG22	9435078	LG22:9435078	UTR, Similar to *txnl4a*: Thioredoxin-like protein 4A (*Mus musculus*)	A	G	UTR

Table S25. Sample size overview. Number of individual samples in the different datasets shown as total, split by sex, sampling year and ecotype/outgroup. Sex split as: N_F (number of females), N_M (number of males) and N_{NA} (number of undetermined sex). Six datasets shown: i) WGS (Whole Genome Sequencing) dataset *full* (including Northeast Arctic cod (NEAC) 1940 and 1958) – sample size including straying cod (NEAC) and bycatch coastal cod (NCC) is shown within brackets, ii) WGS reduced (excluding 1940 and 1958) – sample size including straying cod and bycatch cod shown within brackets, iii) 12K Illumina SNP chip (*29*, *32*, *164*) or the iv) 48 Fluidigm SNP chip (*25*) , v) INV (inversion) dataset (including individuals with scored genotype excluded from WGS due to missingness). vi) mt dataset with additional historical coastal cod (including straying cod and bycatch). Ecotypes are shown as NEAC and NCC (Norwegian coastal cod). Outgroups are shown as NOR (North Sea), BOR (Bornholm) and GOM (Gulf of Maine).

Ecotype/ outgroup	Year	WGS *full*		WGS *reduced*		12k chip		48 chip		INV		mt	
NEAC	1907	27	N_F 16; N_M 10; N_{NA} 1	27	N_F 16; N_M 10; N_{NA} 1					27	N_F 16; N_M 10; N_{NA} 1	27	N_F 16; N_M 10; N_{NA} 1
	1940	21	N_F 10; N_M 11	0	N_F 0; N_M 0					21	N_F 10; N_M 11	21	N_F 10; N_M 11
	1958	15	N_F 8; N_M 7	0	N_F 0; N_M 0					21	N_F 10; N_M 11	15	N_F 8; N_M 7
	1975	11	N_F 8; N_M 3	11	N_F 8; N_M 3					17	N_F 8; N_M 9	11	N_F 8; N_M 3
	1999	11	N_F 3; N_M 8	11	N_F 3; N_M 8					11	N_F 3; N_M 8	11	N_F 3; N_M 8
	2011	22	N_F 15; N_M 7	22	N_F 15; N_M 7	26	N_F 17; N_M 9			48	N_F 32; N_M 16	22	N_F 15; N_M 7
	2012					35	N_F 14; N_M 20; N_{NA} 1			35	N_F 14; N_M 20; N_{NA} 1		
	2014	22 (26)	N_F 11 (14); N_M 11 (12)	22 (26)	N_F 11 (14); N_M 11 (12)			19	N_F 15; N_M 4	41	N_F 26; N_M 15	26	N_F 14; N_M 12
NCC	1958											9	N_F 3; N_M 6
	1975											9	N_F 6; N_M 3
	2011	24 (26)	N_F 11 (12); N_M 13 (14)	24 (26)	N_F 11 (12); N_M 13 (14)	24	N_F 16; N_M 8			48	N_F 27; N_M 21	26	N_F 12; N_M 14
	2014	23 (25)	N_F 11 (13); N_M 11 (11); N_{NA} 1 (1)	23 (25)	N_F 11 (13); N_M 11 (11); N_{NA} 1 (1)			16	N_F 5; N_M 11; N_{NA} 0	39	N_F 16; N_M 22; N_{NA} 1	25	N_F 13; N_M 11; N_{NA} 1
NOR	2002	15	N_F 6; N_M 9	15	N_F 6; N_M 9					15	N_F 6; N_M 9	15	N_F 6; N_M 9
BOR	2012	15	N_F 7; N_M 8	15	N_F 7; N_M 8					15	N_F 7; N_M 8	15	N_F 7; N_M 8
GOM	2009	14	N_F NA; N_M NA	14	N_F NA; N_M NA					14	N_F NA; N_M NA	14	N_F NA; N_M NA

Table S26. Historical WGS samples with sampling and sequencing information. Sample ID given as in file or in abbreviated form. Samples marked with RA indicating that sequencing libraries have been Re-Amplified. Place of landing given as place in Lofoten, Norway where samples were landed or showing the sampling location. Bag ID given as stated on scale- or otolith convolutes. Sex provided as Female =F, Male = M or NA (Non applicable, missing or not determined). Sequencing statistics are followed by statement if samples are present in final datasets. Statements given as yes (3) (samples are present in both WGS datasets and inversion-analysis), yes (2) (are present in one WGS dataset/dataset without 1940 and 1958 and inversion analysis, yes (1) (are present in only the inversion dataset). Reason for exclusion given as mt HET (excluded based on mitochondrial heterozygosity), YE (Year exclusion = 1940 and 1958), DD (data deficiency, missing data) and Coverage (<1x nuclear coverage).

Sample ID	Year of capture	Place of landing	Bag ID	Tissue-type	Broken otolith	Sex	Paired reads (millions)	Average read length (NT or bp)	Coverage nuclear DNA	Endo-genous nuclear DNA	Clonality	Coverage mt	Hetero-zygosity mt	In dataset	Reason if excluded
BM_209	1907	Mortsund	1687	Scale	NA	M	136	84	10.2	58%	2%	107	0.2%	yes (3)	
BM_211	1907	Mortsund	1579	Scale	NA	F	199	77	6.0	25%	2%	100	0.3%	yes (3)	
BM_213	1907	Mortsund	1439	Scale	NA	F	261	66	5.0	18%	2%	55	1.3%	yes (3)	
BM_214	1907	Mortsund	1387	Scale	NA	F	103	98	9.6	61%	2%	85	0.1%	yes (3)	
BM_216	1907	Mortsund	1472	Scale	NA	F	134	83	9.4	54%	2%	48	0.7%	yes (3)	
BM_217	1907	Mortsund	1344	Scale	NA	F	57	96	5.5	65%	2%	64	0.3%	yes (3)	
BM_218	1907	Mortsund	1412	Scale	NA	F	101	109	11.3	65%	2%	106	0.6%	yes (3)	
BM_219	1907	Mortsund	1686	Scale	NA	M	114	84	9.5	63%	1%	95	0.1%	yes (3)	
BM_220	1907	Mortsund	1685	Scale	NA	M	247	78	13.1	44%	3%	208	0.0%	yes (3)	
BM_221	1907	Mortsund	1411	Scale	NA	F	124	81	10.1	64%	2%	138	0.3%	yes (3)	
BM_222	1907	Mortsund	1413	Scale	NA	F	92	89	8.3	65%	1%	88	0.0%	yes (3)	
BM_223	1907	Mortsund	1534	Scale	NA	F	134	85	10.7	60%	1%	100	0.0%	yes (3)	
BM_224	1907	Mortsund	1249	Scale	NA	F	110	91	10.3	66%	1%	131	0.1%	yes (3)	
BM_225	1907	Mortsund	1300	Scale	NA	M	132	85	10.8	61%	2%	111	0.1%	yes (3)	
BM_226	1907	Mortsund	861	Scale	NA	M	96	93	8.6	62%	1%	79	0.0%	yes (3)	
BM_227	1907	Mortsund	862	Scale	NA	M	131	86	11.0	63%	1%	124	0.0%	yes (3)	
BM_230	1907	Mortsund	31	Scale	NA	F	80	91	7.4	66%	1%	112	0.1%	yes (3)	
BM_231	1907	Mortsund	21	Scale	NA	M	97	91	8.5	62%	1%	146	0.1%	yes (3)	
BM_232	1907	Mortsund	182	Scale	NA	F	98	89	8.8	64%	1%	130	0.0%	yes (3)	
BM_234	1907	Mortsund	185	Scale	NA	NA	84	84	6.9	63%	1%	122	1.2%	yes (3)	
BM_236	1907	Mortsund	2	Scale	NA	F	108	85	9.3	64%	2%	152	0.1%	yes (3)	
BM_237	1907	Mortsund	4	Scale	NA	M	97	88	8.3	62%	2%	139	4.0%	no	mt HET
BM_239	1907	Mortsund	6	Scale	NA	M	164	85	12.2	56%	2%	158	0.1%	yes (3)	
BM_240	1907	Mortsund	5	Scale	NA	M	160	70	10.2	59%	2%	145	0.0%	yes (3)	
BM_241	1907	Mortsund	12	Scale	NA	F	157	73	8.9	50%	2%	120	0.1%	yes (3)	
BM_242	1907	Mortsund	183	Scale	NA	F	100	95	9.1	60%	2%	226	0.0%	yes (3)	
BM_243	1907	Mortsund	11	Scale	NA	M	92	87	7.8	62%	1%	125	0.2%	yes (3)	

Table S26 continued on next page

Table S26 continued from previous page

Sample ID	Year of capture	Place of landing	Bag ID	Tissue-type	Broken otolith	Sex	Paired reads (millions)	Average read length (NT or bp)	Coverage nuclear DNA	Endoge nous nuclear DNA	Clonality	Coverage mt	Hetero-zygosity mt	In dataset	Reason if excluded
BM_244	1907	Mortsund	14	Scale	NA	F	282	79	10.9	31%	3%	206	0.0%	yes (3)	
CHC002	1940	Ballstad	2	Otolith	no	M	205	62	4.1	21%	49%	446	0.0%	yes (2)	YE
CHC004	1940	Ballstad	4	Otolith	no	F	138	60	5.3	45%	12%	69	4.6%	no	mt HET
OTL001	1940	Ballstad	8	Otolith	yes	F	130	62	5.9	48%	11%	368	0.0%	yes (2)	YE
OTL004	1940	Ballstad	48	Otolith	no	M	151	60	6.2	46%	20%	273	0.0%	yes (2)	YE
OTL005	1940	Ballstad	58	Otolith	no	M	147	57	4.6	39%	29%	204	0.4%	yes (2)	YE
OTL006	1940	Ballstad	61	Otolith	yes	F	130	60	5.8	49%	23%	146	0.4%	yes (2)	YE
OTL007	1940	Ballstad	73	Otolith	no	F	127	57	4.4	41%	33%	189	0.7%	yes (2)	YE
OTL008	1940	Ballstad	87	Otolith	no	F	140	54	3.8	33%	42%	180	3.2%	no	mt HET
OTL009	1940	Ballstad	88	Otolith	yes	M	112	64	5.2	48%	19%	933	0.0%	yes (2)	YE
OTL010	1940	Ballstad	95	Otolith	no	F	120	57	5.6	56%	4%	55	2.9%	yes (2)	YE
OTL011	1940	Ballstad	98	Otolith	no	M	166	59	5.0	36%	20%	411	0.3%	yes (2)	YE
OTL013	1940	Ballstad	104	Otolith	no	M	113	58	4.6	49%	14%	226	0.9%	yes (2)	YE
OTL014	1940	Ballstad	109	Otolith	no	F	127	59	4.8	46%	13%	833	0.3%	yes (2)	YE
OTL015	1940	Ballstad	113	Otolith	no	M	116	58	6.2	61%	1%	18	0.7%	yes (2)	YE
OTL016	1940	Ballstad	118	Otolith	no	F	133	67	6.5	48%	12%	469	0.1%	yes (2)	YE
OTL017	1940	Ballstad	128	Otolith	yes	M	125	70	7.0	52%	8%	467	0.1%	yes (2)	YE
OTL018	1940	Ballstad	134	Otolith	no	M	162	66	5.6	36%	25%	809	0.1%	yes (2)	YE
OTL019	1940	Ballstad	135	Otolith	no	F	126	57	5.6	52%	9%	239	0.0%	yes (2)	YE
OTL020	1940	Ballstad	142	Otolith	yes	M	178	59	5.8	39%	14%	539	0.0%	yes (2)	YE
OTL021	1940	Ballstad	17	Otolith	no	F	168	67	6.9	41%	14%	922	0.0%	yes (2)	YE
OTL022	1940	Ballstad	59	Otolith	no	F	121	63	6.4	56%	12%	687	0.1%	yes (2)	YE
OTL023	1940	Ballstad	78	Otolith	no	M	119	59	6.1	58%	7%	265	0.0%	yes (2)	YE
OTL024	1940	Ballstad	105	Otolith	no	F	124	62	6.9	60%	3%	90	1.6%	yes (2)	YE
OTL026	1958	Røstegga	2	Otolith	no	M	153	42	4.5	47%	17%	93	3.4%	no	mt HET
OTL027	1958	Røstegga	6	Otolith	yes	F	138	43	4.0	45%	22%	376	0.0%	yes (1)	DD
OTL028	1958	Røstegga	7	Otolith	no	M	80	46	2.9	52%	16%	114	0.1%	yes (1)	DD
OTL029	1958	Røstegga	15	Otolith	no	M	219	43	6.8	48%	18%	99	0.2%	yes (2)	YE
OTL030	1958	Røstegga	18	Otolith	yes	M	269	40	6.5	43%	20%	47	2.8%	yes (2)	YE
OTL031	1958	Røstegga	20	Otolith	yes	M	204	41	5.2	42%	15%	187	1.8%	yes (2)	YE
OTL032	1958	Røstegga	26	Otolith	yes	M	168	40	3.8	39%	29%	270	0.2%	yes (1)	DD
OTL033	1958	Røstegga	29	Otolith	yes	F	207	59	8.2	43%	31%	912	0.1%	yes (2)	YE
OTL034	1958	Røstegga	31	Otolith	yes	M	114	46	3.6	47%	2%	12	2.1%	yes (1)	DD
OTL035	1958	Røstegga	33	Otolith	yes	F	148	54	6.4	53%	7%	253	0.4%	yes (2)	YE
OTL036	1958	Røstegga	35	Otolith	yes	M	130	58	6.7	58%	5%	308	0.0%	yes (2)	YE
OTL037	1958	Røstegga	36	Otolith	no	F	165	58	6.5	44%	12%	198	1.8%	yes (2)	YE

Table S26 continued on next page

Table S26 continued from previous page

Sample ID	Year of capture	Place of landing	Bag ID	Tissue-type	Broken otolith	Sex	Paired reads (millions)	Average read length (NT or bp)	Coverage nuclear DNA	Endo-genous nuclear DNA	Clonality	Coverage mt	Hetero-zygosity mt	In dataset	Reason if excluded
OTL038	1958	Røstegga	43	Otolith	yes	F	151	58	7.1	53%	2%	31	4.5%	no	mt HET
OTL039	1958	Røstegga	44	Otolith	no	M	77	62	1.9	26%	49%	113	0.3%	yes (1)	DD
OTL040	1958	Røstegga	48	Otolith	yes	F	118	61	6.2	56%	5%	890	0.1%	yes (2)	YE
OTL041	1958	Røstegga	50	Otolith	no	F	135	57	7.2	60%	4%	134	0.1%	yes (2)	YE
OTL042	1958	Røstegga	54	Otolith	no	F	107	54	5.3	61%	4%	326	0.1%	yes (2)	YE
OTL043	1958	Røstegga	56	Otolith	yes	M	112	64	6.5	58%	9%	316	0.1%	yes (2)	YE
OTL044	1958	Røstegga	65	Otolith	yes	M	116	63	6.4	57%	7%	219	0.4%	yes (2)	YE
OTL045	1958	Røstegga	66	Otolith	yes	M	108	61	5.9	59%	5%	300	0.1%	yes (2)	YE
OTL046	1958	Røstegga	69	Otolith	yes	F	128	66	4.4	34%	38%	334	0.0%	yes (2)	YE
OTL047	1958	Røstegga	73	Otolith	yes	F	132	57	6.6	58%	5%	374	0.1%	yes (2)	YE
OTL048	1958	Røstegga	77	Otolith	yes	F	93	65	1.6	18%	63%	128	0.0%	yes (1)	DD
OTL050	1975	Værøy	5	Otolith	no	M	99	79	7.1	58%	14%	2035	0.0%	yes (3)	
OTL051	1975	Værøy	13	Otolith	no	M	105	75	6.9	56%	16%	1071	0.0%	yes (3)	
OTL053RA	1975	Værøy	16	Otolith	yes	M	163	103	3.2	12%	76%	328	0.1%	yes (1)	DD
OTL054	1975	Værøy	25	Otolith	no	F	101	87	6.5	47%	29%	398	0.0%	yes (3)	
OTL055RA	1975	Værøy	26	Otolith	no	F	30	89	0.8	22%	64%	67	NA	no	Coverage
OTL056	1975	Værøy	29	Otolith	no	M	111	77	4.6	34%	40%	228	0.0%	yes (3)	
OTL057RA	1975	Værøy	30	Otolith	no	M	197	95	2.5	8%	86%	35	0.2%	yes (1)	DD
OTL058RA	1975	Værøy	32	Otolith	no	F	115	87	4.1	26%	57%	375	0.1%	yes (3)	
OTL059RA	1975	Værøy	36	Otolith	no	M	210	83	2.2	9%	84%	117	0.0%	yes (1)	DD
OTL060RA	1975	Værøy	44	Otolith	no	F	139	81	5.2	30%	53%	442	0.1%	yes (3)	
OTL062RA	1975	Værøy	49	Otolith	no	M	230	65	2.2	9%	82%	525	0.0%	yes (1)	DD
OTL064	1975	Værøy	53	Otolith	yes	F	93	70	5.6	55%	17%	288	0.0%	yes (3)	
OTL066	1975	Værøy	58	Otolith	no	F	103	55	4.1	47%	27%	274	0.0%	yes (3)	
OTL067	1975	Værøy	67	Otolith	yes	M	100	75	2.0	17%	72%	176	0.0%	yes (1)	DD
OTL068	1975	Værøy	68	Otolith	no	F	97	66	4.8	48%	27%	375	0.0%	yes (3)	
OTL069	1975	Værøy	71	Otolith	no	F	100	69	4.2	39%	26%	78	0.0%	yes (3)	
OTL070	1975	Værøy	72	Otolith	no	F	6	76	0.3	39%	25%	34	NA	no	Coverage
OTL071	1975	Værøy	74	Otolith	no	F	117	83	3.6	23%	55%	114	0.1%	yes (3)	
OTL072	1975	Værøy	75	Otolith	no	M	136	66	2.1	15%	66%	72	0.7%	yes (1)	DD
OTL092	1999	80717	49	Otolith	no	F	66	107	6.7	59%	2%	527	0.0%	yes (3)	
OTL093	1999	80717	23	Otolith	yes	F	47	110	5.1	62%	1%	323	0.0%	yes (3)	
OTL094	1999	80717	43	Otolith	yes	F	220	90	19.1	62%	6%	892	0.0%	yes (3)	
OTL095RA	1999	80717	22	Otolith	yes	F	32	47	0.7	30%	45%	35	NA	no	Coverage
OTL096	1999	80717	21	Otolith	yes	F	164	54	6.2	45%	16%	80	6.6%	no	mt HET
OTL098	1999	80717	50	Otolith	yes	M	143	72	8.3	51%	14%	226	0.1%	yes (3)	

Table S26 continued on next page

Table S26 continued from previous page

Sample ID	Year of capture	Place of landing	Bag ID	Tissue-type	Broken otolith	Sex	Paired reads (millions)	Average read length (NT or bp)	Coverage nuclear DNA	Endo-genous nuclear DNA	Clonality	Coverage mt	Hetero-zygosity mt	In dataset	Reason if excluded
OTL099	1999	80717	3	Otolith	yes	M	156	85	10.1	48%	14%	397	0.1%	yes (3)	
OTL100	1999	80717	34	Otolith	yes	M	118	85	10.1	63%	5%	395	0.1%	yes (3)	
OTL101RA	1999	80717	24	Otolith	yes	M	141	91	5.2	27%	56%	514	0.9%	yes (3)	
OTL102RA	1999	80717	28	Otolith	yes	M	131	103	11.7	54%	17%	937	0.1%	yes (3)	
OTL103	1999	80717	12	Otolith	no	M	251	74	17.3	59%	7%	490	0.0%	yes (3)	
OTL104RA	1999	80717	33	Otolith	yes	M	139	77	3.8	23%	59%	138	2.4%	yes (3)	
OTL105RA	1999	80717	55	Otolith	yes	M	121	79	8.7	57%	11%	234	3.8%	no	mt HET
OTL106RA	1999	80717	40	Otolith	yes	M	146	82	5,7	30%	49%	249	2.4%	yes (3)	

Table S27. Historical coastal samples with sampling and sequencing information. Sample ID given as in file or in abbreviated form. Samples marked with RA indicating that sequencing libraries have been Re-Amplified. Place of landing given as place in Lofoten, Norway where samples were landed or showing the sampling location. Bag ID given as stated on scale- or otolith convolutes. Sex provided as Female =F, Male = M or NA (Non applicable, missing or not determined). Sequencing statistics are followed by statement if samples are present in final datasets. Statements given as yes (3) (samples are present in both WGS datasets and inversion-analysis), yes (2) (are present in one WGS dataset/dataset without 1940 and 1958 and inversion analysis, yes (1) (are present in only the inversion dataset). Reason for exclusion given as mt HET (excluded based on mitochondrial heterozygosity), YE (Year exclusion = 1940 and 1958), DD (data deficiency, missing data) and Coverage (<1x nuclear coverage).

Sample ID	Year of capture	Place of landing	Bag ID	Tissue-type	Broken otolith	Sex	Paired reads (millions)	Coverage nuclear DNA	Endo-genous nuclear DNA	Clonality	Coverage mt	Endo-genous mt DNA	Hetero-zygosity mt	In WGS dataset	Reason if excluded
OTL_117_R	1958	Røstegga	16	Otolith	yes	F	118	0.7	7%	76%	59	0.02%	0.2%	no	Coverage
OTL_118_R	1958	Røstegga	25	Otolith	yes	F	8	0.1	14%	4%	5	0.02%	0.9%	no	Coverage
OTL_119	1958	Røstegga	28	Otolith	no	M	3	0.2	60%	1%	21	0.17%	0.3%	no	Coverage
OTL_120_R	1958	Røstegga	30	Otolith	yes	F	9	0.4	49%	12%	24	0.07%	0.2%	no	Coverage
OTL_122_R	1958	Røstegga	37	Otolith	no	M	4	0.2	55%	5%	30	0.20%	0.1%	no	Coverage
OTL_123_R	1958	Røstegga	40	Otolith	yes	M	7	0.1	15%	4%	2	0.01%	0.4%	no	Coverage
OTL_124_R	1958	Røstegga	49	Otolith	no	M	10	0.4	42%	28%	27	0.08%	0.1%	no	Coverage
OTL_125_R	1958	Røstegga	70	Otolith	no	M	12	0.8	59%	3%	54	0.13%	0.0%	no	Coverage
OTL_127_R	1958	Røstegga	88	Otolith	no	M	6	0.3	45%	17%	13	0.05%	1.0%	no	Coverage
OTL_128_R	1975	Værøy	9	Otolith	no	F	8	0.5	54%	12%	56	0.19%	0.1%	no	Coverage
OTL_129	1975	Værøy	10	Otolith	no	M	4	0.3	60%	2%	17	0.11%	0.1%	no	Coverage
OTL_130_R	1975	Værøy	20	Otolith	no	F	12	0.5	29%	53%	104	0.19%	0.0%	no	Coverage
OTL_132_R	1975	Værøy	24	Otolith	no	F	7	0.5	61%	4%	20	0.07%	0.0%	no	Coverage
OTL_133_R	1975	Værøy	35	Otolith	no	F	9	0.7	63%	3%	11	0.03%	0.6%	no	Coverage
OTL_134_R	1975	Værøy	40	Otolith	no	M	1	0.1	58%	5%	7	0.16%	0.1%	no	Coverage
OTL_135	1975	Værøy	45	Otolith	no	F	1	4.4E-02	62%	1%	8	0.32%	0.1%	no	Coverage
OTL_136	1975	Værøy	48	Otolith	no	F	2	0.1	64%	1%	18	0.22%	0.0%	no	Coverage
OTL_137_R	1975	Værøy	60	Otolith	no	M	5	0.3	52%	15%	34	0.18%	0.0%	no	Coverage

Table S28. Inversion boundaries. Inversion boundaries (*29*) showing linkage group (LG), SNP position and name for initial borders, and corresponding positions in gadMor2 (*115*).

**Linkage group**	**SNP** *Position*	**Name**	**Linkage group**	**Position in gadMor2** *bp*
LG1	134–417	ss1712303910-ss1712300855	LG01	8615637-26153351
LG2	749–835	ss1712300970-ss1712298100	LG02	18327047-24034565
LG7	2537–2720	ss1712298657-ss1712300745	LG07	13526494-23089445
LG12	4248–4444	ss1712298449-ss1712300081	LG12	467893-13345985

Table S29. Inversion boundaries. Inversion boundaries showing linkage group (LG), positions (start and stop) for boundaries excluding inversions and for looking within inversions. Number of sites excluded and within inversions given as well for the dataset without 1940 and 1958 (N=192) and dataset with 1940 and 1958 (N=228). Data for dataset without 1940 and 1958 given first in column, data for with given second). Confidence given as well.

**Linkage group**	***Excluding*** **Start** *Position*	**Stop** *Position*	***Within*** **Start** *Position*	**Stop** *Position*	**Number of sites** *Excluded*	**Number of sites** *Within*	**Confidence Size** *Bp*
LG01	9015000	26325000	9165000	26130000	6848<br>3324	6767<br>3289	±75kb and<br>-120kb/+75kb
LG02	18405000	24054406	18555000<br>23220000	22035000<br>23969406	1655<br>915	1425<br>800	±75kb, ±75kb<br>-85kb
LG07	13515000	23115000	13665000	22965000	4837<br>2936	4764<br>2905	±75kb
LG12	1	13920000	925001	13320000	3841<br>2129	3798<br>2113	+925.001kb,<br>±300kb

Table S30. Results of the relationship exploration using GAM model with a smooth or a linear formulation. quasi-AIC (qAIC) calculated for the models presented in the paper: gam(proportion of different genotype ~ s(x), weights=count of fish each year, quasibinomial, method="REML", scale=1) and the linear version of the same model: gam(proportion of different genotype ~ x, weights=count of fish each year, quasibinomial, method="REML", scale=1). 'edf' is the estimated degrees of freedom of the smoothing term for the first formulation. A "–" indicates that no model was found (see **table S19**). A red "–" indicates that no linear model was found and thus no qAIC was calculated. In italic are indicated the comparisons where no difference was expected, the GAM having shrinked the smooth toward linear (edf=1). qAIC is an estimated quasi-Akaike Information Criterion using the formula qAIC = model deviance/ĉ + 2 ·k · ĉ, with k the effective degrees of freedom, ĉ the dispersion parameter. $F_{5.10}$: Fishing mortality, μ: generation time, NAO: North Atlantic Oscillation, Recruits: number of recruits, SSB: spawning stock biomass, and ST: Kola section sea temperature.

Variable		LG01						LG02			LG07			LG12			Val-Ala		
		DERIVED			ANCESTRAL			ANCESTRAL			DERIVED			ANCESTRAL					
		~s(x)		~x	~s(x)		~x	~s(x)		~x	~s(x)		~x	~s(x)		~x	~s(x)		~x
		edf	qAIC	qAIC	edf	qAIC	qAIC	edf	qAIC	qAIC	edf	qAIC	qAIC	edf	qAIC	qAIC	edf	qAIC	qAIC
$F_{5.10}$		1	*31*	*31*	1	*29*	*29*	1	*29*	*29*	1.34	29	30	1	*28*	*28*	–	–	–
μ		1	*32*	*32*	1	*30*	*30*	1	*29*	*29*	1	*30*	*30*	1	*29*	*29*	–	–	–
mean NAO	before	–	–	–	1	*39*	*39*	1	*39*	*39*	–	–	–	1.72	38	–	–	–	–
max NAO		1.74	44	43	1.05	39	39	1	*38*	*38*	–	–	–	1.17	38	38	1.83	39	39
mean NAO	after	–	–	–	–	–	–	–	–	–	–	–	–	–	–	–	–	–	–
max NAO		1.82	44	–	1.6	39	39	1.28	39	39	–	–	–	1	*38*	*38*	1.84	38	39
Recruits		–	–	–	–	–	–	1	*30*	*30*	–	30	–	1	*29*	*29*	1	28	28
SSB		1.84	39	38	1	34	34	1.71	35	–	1.6	34	34	1.72	33	–	1.77	34	–
mean ST	before	1	*42*	*42*	1.67	40	–	1.66	39	–	–	–	–	1	*38*	*38*	–	–	–
max ST		1.47	43	42	1.85	39	–	1.83	39	–	–	–	–	–	–	–	1.19	39	39
mean ST	after	1.29	42	41	1.54	39	–	1.72	39	39	–	–	–	1.09	38	38	–	–	–
max ST		1.43	43	42	–	–	–	1.57	39	39	–	–	–	1.23	38	38	1.46	39	39

REFERENCES AND NOTES

1. S. C. Doney, M. Ruckelshaus, J. E. Duffy, J. P. Barry, F. Chan, C. A. English, H. M. Galindo, J. M. Grebmeier, A. B. Hollowed, N. Knowlton, J. Polovina, N. N. Rabalais, W. J. Sydeman, L. D. Talley, Climate change impacts on marine ecosystems. *Ann. Rev. Mar. Sci.* **4**, 11–37 (2012).

2. P. E. Renaud, J. Berge, Ø. Varpe, O. J. Lønne, J. Nahrgang, C. Ottesen, I. Hallanger, Is the poleward expansion by Atlantic cod and haddock threatening native polar cod, *Boreogadus saida*? *Polar Biol* **35**, 401–412 (2012).

3. M. L. Pinsky, B. Worm, M. J. Fogarty, J. L. Sarmiento, S. A. Levin, Marine taxa track local climate velocities. *Science* **341**, 1239–1242 (2013).

4. M. Heino, B. D. Pauli, U. Dieckmann, Fisheries-induced evolution. *Annu. Rev. Ecol. Evol. Syst.* **46**, 461–480 (2014).

5. A. Kuparinen, J. A. Hutchings, Genetic architecture of age at maturity can generate divergent and disruptive harvest-induced evolution. *Philos. Trans. R Soc. Lond. B Biol. Sci.* **372**, 20160035 (2017).

6. M. L. Pinsky, A. M. Eikeset, C. Helmerson, I. R. Bradbury, P. Bentzen, C. Morris, A. T. Gondek-Wyrozemska, H. T. Baalsrud, M. S. O. Brieuc, O. S. Kjesbu, J. A. Godiksen, J. M. I. Barth, M. Matschiner, N. C. Stenseth, K. S. Jakobsen, S. Jentoft, B. Star, Genomic stability through time despite decades of exploitation in cod on both sides of the Atlantic. *Proc. Natl. Acad. Sci. U.S.A.* **118**, e2025453118 (2021).

7. J. A. Hutchings, A. Kuparinen, Throwing down a genomic gauntlet on fisheries-induced evolution. *Proc. Natl. Acad. Sci. U.S.A.* **118**, e2105319118 (2021).

8. N. O. Therkildsen, A. P. Wilder, D. O. Conover, S. B. Munch, H. Baumann, S. R. Palumbi, Contrasting genomic shifts underlie parallel phenotypic evolution in response to fishing. *Science* **365**, 487–490 (2019).

9. A. Kuparinen, J. A. Hutchings, Consequences of fisheries-induced evolution for population productivity and recovery potential. *Proc. Biol. Sci.* **279**, 2571–2579 (2012).

10. G. A. Rose, *Atlantic Cod: A Bio-Ecology* (Wiley, Hoboken, NJ, USA, 2019).

11. O. S. Kjesbu, B. Bogstad, J. A. Devine, H. Gjøsæter, D. Howell, R. B. Ingvaldsen, R. D. M. Nash, J. E. Skjæraasen, Synergies between climate and management for Atlantic cod fisheries at high latitudes. *Proc. Natl. Acad. Sci. U.S.A.* **111**, 3478–3483 (2014).

12. G. Ottersen, B. Bogstad, N. A. Yaragina, L. C. Stige, F. B. Vikebø, P. Dalpadado, A review of early life history dynamics of Barents Sea cod (*Gadus morhua*). *ICES J. Mar. Sci.* **71**, 2064–2087 (2014).

13. K. M. Brander, The role of growth changes in the decline and recovery of North Atlantic cod stocks since 1970. *ICES J. Mar. Sci.* **64**, 211–217 (2007).

14. M. Heino, U. Dieckmann, O. R. Godø, "Reaction norm analysis of fisheries-induced adaptive change and the case of the Northeast Arctic cod" (2002). ICES CM Documents 2002 – ICES Annual Science Conference, 1–5 October 2002.

15. G. Ottersen, R. E. Holt, Long-term variability in spawning stock age structure influences climate–recruitment link for Barents Sea cod. *Fish. Oceanogr.* **32**, 91–105 (2023).

16. D. Chu, Technology evolution and advances in fisheries acoustics. *J Mar Sci Technol* **19**, DOI: 10.51400/2709-6998.2188 (2011).

17. P. Holm, World War II and the "great acceleration" of North Atlantic fisheries. *Global Environ.* **10**, 66–91 (2012).

18. P. Greenberg, The Expansion of Global Fishing, *The Expansion of Global Fishing* (2014). https://earthjournalism.net/resources/the-expansion-of-global-fishing.

19. A. Hayden, J. Acheson, M. Kersula, J. Wilson, Spatial and temporal patterns in the cod fisheries of the North Atlantic. *Conserv. Soc.* **13**, 414 (2015).

20. G. Ottersen, Pronounced long-term juvenation in the spawning stock of Arcto-Norwegian cod (*Gadus morhua*) and possible consequences for recruitment. *Can. J. Fish. Aquat. Sci.* **65**, 523–534 (2008).

21. P. Vasilakopoulos, F. G. O'Neill, C. T. Marshall, Misspent youth: Does catching immature fish affect fisheries sustainability? *ICES J. Mar. Sci.* **68**, 1525–1534 (2011).

22. B. N. Reid, B. Star, M. L. Pinsky, Detecting parallel polygenic adaptation to novel evolutionary pressure in wild populations: A case study in Atlantic cod (*Gadus morhua*). *Philos. Trans. R. Soc. B* **378**, 20220190 (2023).

23. G. Dahle, T. Johansen, J.-I. Westgaard, A. Aglen, K. A. Glover, Genetic management of mixed-stock fisheries "real-time": The case of the largest remaining cod fishery operating in the Atlantic in 2007–2017. *Fish. Res.* **205**, 77–85 (2018).

24. T. Johansen, J.-I. Westgaard, B. B. Seliussen, K. Nedreaas, G. Dahle, K. A. Glover, R. Kvalsund, A. Aglen, "Real-time" genetic monitoring of a commercial fishery on the doorstep of an MPA reveals unique insights into the interaction between coastal and migratory forms of the Atlantic cod. *ICES J. Mar. Sci.* **75**, 1093–1104 (2018).

25.. Langangen, L. Färber, L. C. Stige, F. K. Diekert, J. M. I. Barth, M. Matschiner, P. R. Berg, B. Star, N. C. Stenseth, S. Jentoft, J. M. Durant, Ticket to spawn: Combining economic and genetic data to evaluate the effect of climate and demographic structure on spawning distribution in Atlantic cod. *Glob. Chang. Biol.* **25**, 134–143 (2019).

26. S. Sundby, O. Nakken, Spatial shifts in spawning habitats of Arcto-Norwegian cod related to multidecadal climate oscillations and climate change. *ICES J. Mar. Sci.* **65**, 953–962 (2008).

27. J. E. Skjæraasen, R. D. M. Nash, K. Korsbrekke, M. Fonn, T. Nilsen, J. Kennedy, K. H. Nedreaas, A. Thorsen, P. R. Witthames, A. J. Geffen, H. Høie, O. S. Kjesbu, Frequent skipped spawning in the world's largest cod population. *Proc. Natl. Acad. Sci. U.S.A.* **109**, 8995–8999 (2012).

28. H. Höffle, P. Solemdal, K. Korsbrekke, M. Johannessen, K. Bakkeplass, O. S. Kjesbu, Variability of northeast Arctic cod (*Gadus morhua*) distribution on the main spawning grounds in relation to biophysical factors. *ICES J. Mar. Sci.* **71**, 1317–1331 (2014).

29. P. R. Berg, B. Star, C. Pampoulie, M. Sodeland, J. M. I. Barth, H. Knutsen, K. S. Jakobsen, S. Jentoft, Three chromosomal rearrangements promote genomic divergence between migratory and stationary ecotypes of Atlantic cod. *Sci. Rep.* **6**, 23246 (2016).

30. J. M. I. Barth, D. Villegas-Ríos, C. Freitas, E. Moland, B. Star, C. André, H. Knutsen, I. Bradbury, J. Dierking, C. Petereit, D. Righton, J. Metcalfe, K. S. Jakobsen, E. M. Olsen, S. Jentoft, Disentangling structural genomic and behavioural barriers in a sea of connectivity. *Mol. Ecol.* **28**, 1394–1411 (2019).

31. M. Matschiner, J. M. I. Barth, O. K. Tørresen, B. Star, H. T. Baalsrud, M. S. O. Brieuc, C. Pampoulie, I. Bradbury, K. S. Jakobsen, S. Jentoft, Supergene origin and maintenance in Atlantic cod. *Nat. Ecol. Evol.* **6**, 469–481 (2022).

32. P. R. Berg, S. Jentoft, B. Star, K. H. Ring, H. Knutsen, S. Lien, K. S. Jakobsen, C. André, Adaptation to low salinity promotes genomic divergence in Atlantic cod (*Gadus morhua* L.). *Genome Biol. Evol.* **7**, 1644–1663 (2015).

33. J. M. I. Barth, P. R. Berg, P. R. Jonsson, S. Bonanomi, H. Corell, J. Hemmer-Hansen, K. S. Jakobsen, K. Johannesson, P. E. Jorde, H. Knutsen, P. Moksnes, B. Star, N. C. Stenseth, H. Svedäng, S. Jentoft, C. André, Genome architecture enables local adaptation of Atlantic cod despite high connectivity. *Mol. Ecol.* **26**, 4452–4466 (2017).

34. K. Sick, Hæmoglobin polymorphism in fishes. *Nature* **192**, 894–896 (1961).

35.. Andersen, O. F. Wetten, M. C. D. Rosa, C. Andre, C. C. Alinovi, M. Colafranceschi, O. Brix, A. Colosimo, Haemoglobin polymorphisms affect the oxygen-binding properties in Atlantic cod populations. *Proc. Biol. Sci.* **276**, 833–841 (2009).

36. S. D. Ross, J. W. Behrens, K. Brander, C. Methling, J. Mork, Haemoglobin genotypes in cod (*Gadus morhua* L): Their geographic distribution and physiological significance. *Comp. Biochem. Physiol. Part A: Mol. Integr. Physiol.* **166**, 158–168 (2013).

37. V. D. Boitsov, A. L. Karsakov, A. G. Trofimov, Atlantic water temperature and climate in the Barents Sea, 2000–2009. *ICES J. Mar. Sci.* **69**, 833–840 (2012).

38. T. G. Kirubakaran, H. Grove, M. P. Kent, S. R. Sandve, M. Baranski, T. Nome, M. C. D. Rosa, B. Righino, T. Johansen, H. Otterå, A. Sonesson, S. Lien, Ø. Andersen, Two adjacent inversions maintain genomic differentiation between migratory and stationary ecotypes of Atlantic cod. *Mol. Ecol.* **25**, 2130–2143 (2016).

39. T. Kess, P. Bentzen, S. J. Lehnert, E. V. A. Sylvester, S. Lien, M. P. Kent, M. Sinclair-Waters, C. J. Morris, P. Regular, R. Fairweather, I. R. Bradbury, A migration-associated supergene reveals loss of biocomplexity in Atlantic cod. *Sci. Adv.* **5**, eaav2461 (2019).

40. N. Patterson, A. L. Price, D. Reich, Population structure and eigenanalysis. *Plos Genet.* **2**, e190 (2006).

41. A. L. Price, N. J. Patterson, R. M. Plenge, M. E. Weinblatt, N. A. Shadick, D. Reich, Principal components analysis corrects for stratification in genome-wide association studies. *Nat. Genet.* **38**, 904–909 (2006).

42. D. H. Alexander, J. Novembre, K. Lange, Fast model-based estimation of ancestry in unrelated individuals. *Genome Res.* **19**, 1655–1664 (2009).

43. P. E. Jorde, N. Ryman, Unbiased estimator for genetic drift and effective population size. *Genetics* **177**, 927–935 (2007).

44. R. S. Waples, A generalized approach for estimating effective population size from temporal changes in allele frequency. *Genetics* **121**, 379–391 (1989).

45. S. N. Wood, Generalized Additive Models: An introduction with R. *Texts in Statistical Sciences Series* (2006).

46. R. Ingvaldsen, H. Loeng, B. Ådlandsvik, G. Ottersen, Climate variability in the Barents Sea during the 20th century with focus on the 1990s. *CES Mar. Sci. Symp.* **219**, 160–168 (2003).

47. J. W. Hurrell, C. Deser, North Atlantic climate variability: The role of the North Atlantic Oscillation. *J. Mar. Syst.* **78**, 28–41 (2009).

48. W. E. Ricker, Computation and interpretation of biological statistics of fish populations. *Bulletin of the Fisheries Research Board of Canada*, 1–382 (1975).

49. F. Tajima, Statistical method for testing the neutral mutation hypothesis by DNA polymorphism. *Genetics* **123**, 585–595 (1989).

50. B. F. Voight, S. Kudaravalli, X. Wen, J. K. Pritchard, A map of recent positive selection in the human genome. *PLoS Biol.* **4**, e72 (2006).

51. B. S. Weir, C. C. Cockerham, Estimating F-statistics for the analysis of population structure. *Evolution* **38**, 1358–1370 (1984).

52. D. Berner, Allele frequency difference AFD – An intuitive alternative to FST for quantifying genetic population differentiation. *Genes* **10**, 308 (2019).

53. P. C. Sabeti, P. Varilly, B. Fry, J. Lohmueller, E. Hostetter, C. Cotsapas, X. Xie, E. H. Byrne, S. A. McCarroll, R. Gaudet, S. F. Schaffner, E. S. Lander, International Hap Map Consortium, K. A. Frazer, D. G. Ballinger, D. R. Cox, D. A. Hinds, L. L. Stuve, R. A. Gibbs, J. W. Belmont, A. Boudreau, P. Hardenbol, S. M. Leal, S. Pasternak, D. A. Wheeler, T. D. Willis, F. Yu, H. Yang, C. Zeng, Y. Gao, H. Hu, W. Hu, C. Li, W. Lin, S. Liu, H. Pan, X. Tang, J. Wang, W. Wang, J. Yu, B. Zhang, Q. Zhang, H. Zhao, H. Zhao, J. Zhou, S. B. Gabriel, R. Barry, B. Blumenstiel, A. Camargo, M. Defelice, M. Faggart, M. Goyette, S. Gupta, J. Moore, H. Nguyen, R. C. Onofrio, M. Parkin, J. Roy, E. Stahl, E. Winchester, L. Ziaugra, D. Altshuler, Y. Shen, Z. Yao, W. Huang, X. Chu, Y. He, L. Jin, Y. Liu, Y. Shen, W. Sun, H. Wang, Y. Wang, Y. Wang, X. Xiong, L. Xu, M. M. Y. Waye, S. K. W. Tsui, H. Xue, J. T.-F. Wong, L. M. Galver, J.-B. Fan, K. Gunderson, S. S. Murray, A. R. Oliphant, M. S. Chee, A. Montpetit, F. Chagnon, V. Ferretti, M. Leboeuf, J.-F. Olivier, M. S. Phillips, S. Roumy, C. Sallée, A. Verner, T. J. Hudson, P.-Y. Kwok, D. Cai, D. C. Koboldt, R. D. Miller, L. Pawlikowska, P.

Taillon-Miller, M. Xiao, L.-C. Tsui, W. Mak, Y. Q. Song, P. K. H. Tam, Y. Nakamura, T. Kawaguchi, T. Kitamoto, T. Morizono, A. Nagashima, Y. Ohnishi, A. Sekine, T. Tanaka, T. Tsunoda, P. Deloukas, C. P. Bird, M. Delgado, E. T. Dermitzakis, R. Gwilliam, S. Hunt, J. Morrison, D. Powell, B. E. Stranger, P. Whittaker, D. R. Bentley, M. J. Daly, P. I. W. de Bakker, J. Barrett, Y. R. Chretien, J. Maller, S. M. Carroll, N. Patterson, I. Pe'er, A. Price, S. Purcell, D. J. Richter, P. Sabeti, R. Saxena, S. F. Schaffner, P. C. Sham, P. Varilly, D. Altshuler, L. D. Stein, L. Krishnan, A. V. Smith, M. K. Tello-Ruiz, G. A. Thorisson, A. Chakravarti, P. E. Chen, D. J. Cutler, C. S. Kashuk, S. Lin, G. R. Abecasis, W. Guan, Y. Li, H. M. Munro, Z. S. Qin, D. J. Thomas, G. M. Vean, A. Auton, L. Bottolo, N. Cardin, S. Eyheramendy, C. Freeman, J. Marchini, S. Myers, C. Spencer, M. Stephens, P. Donnelly, L. R. Cardon, G. Clarke, D. M. Evans, A. P. Morris, B. S. Weir, T. Tsunoda, T. A. Johnson, J. C. Mullikin, S. T. Sherry, M. Feolo, A. Skol, H. Zhang, C. Zeng, H. Zhao, I. Matsuda, Y. Fukushima, D. R. Macer, E. Suda, C. N. Rotimi, C. A. Adebamowo, I. Ajayi, T. Aniagwu, P. A. Marshall, C. Nkwodimmah, C. D. M. Royal, M. F. Leppert, M. Dixon, A. Peiffer, R. Qiu, A. Kent, K. Kato, N. Niikawa, I. F. Adewole, B. M. Knoppers, M. W. Foster, E. W. Clayton, J. Watkin, R. A. Gibbs, J. W. Belmont, D. Muzny, L. Nazareth, E. Sodergren, G. M. Weinstock, D. A. Wheeler, I. Yakub, S. B. Gabriel, R. C. Onofrio, D. J. Richter, L. Ziaugra, B. W. Birren, M. J. Daly, D. Altshuler, R. K. Wilson, L. L. Fulton, J. Rogers, J. Burton, N. P. Carter, C. M. Clee, M. Griffiths, M. C. Jones, K. M. Lay, R. W. Plumb, M. T. Ross, S. K. Sims, D. L. Willey, Z. Chen, H. Han, L. Kang, M. Godbout, J. C. Wallenburg, P. L'Archevêque, G. Bellemare, K. Saeki, H. Wang, D. An, H. Fu, Q. Li, Z. Wang, R. Wang, A. L. Holden, L. D. Brooks, J. E. M. Ewen, M. S. Guyer, V. O. Wang, J. L. Peterson, M. Shi, J. Spiegel, L. M. Sung, L. F. Zacharia, F. S. Collins, K. Kennedy, R. Jamieson, J. Stewart, Genome-wide detection and characterization of positive selection in human populations. *Nature* **449**, 913–918 (2007).

54. M. Fardi, M. Alivand, B. Baradaran, M. F. Hagh, S. Solali, The crucial role of ZEB2: From development to epithelial-to-mesenchymal transition and cancer complexity. *J. Cell. Physiol.* **234**, 14783–14799 (2019).

55. B. L. Nelms, P. A. Labosky, *Transcriptional Control of Neural Crest Development* (Morgan & Claypool Life Sciences, San Rafael (CA), 2010).

56. A. A. Brandon, D. Almeida, K. E. Powder, Neural crest cells as a source of microevolutionary variation. *Semin. Cell Dev. Biol.* **145**, 42–51 (2023).

57. Y. Nakagawa, H. Takamatsu, T. Okuno, S. Kang, S. Nojima, T. Kimura, T. R. Kataoka, M. Ikawa, T. Toyofuku, I. Katayama, A. Kumanogoh, Identification of Semaphorin 4B as a negative regulator of basophil-mediated immune responses. *J. Immunol.* **186**, 2881–2888 (2011).

58. R.-M. Ferraiuolo, K. C. Manthey, M. J. Stanton, A. A. Triplett, K.-U. Wagner, The multifaceted roles of the tumor susceptibility gene 101 (TSG101) in normal development and disease. *Cancers* **12**, 450 (2020).

59. R. M. Judy, C. J. Sheedy, B. M. Gardner, Insights into the structure and function of the Pex1/Pex6 AAA-ATPase in peroxisome homeostasis. *Cells* **11**, 2067 (2022).

60. P. V. Candelaria, L. S. Leoh, M. L. Penichet, T. R. Daniels-Wells, Antibodies targeting the transferrin receptor 1 (TfR1) as direct anti-cancer agents. *Front. Immunol.* **12**, 607692 (2021).

61. P. Aisen, Transferrin receptor 1. *Int. J. Biochem. Cell Biol.* **36**, 2137–2143 (2004).

62. L. Olazcuaga, A. Loiseau, H. Parrinello, M. Paris, A. Fraimout, C. Guedot, L. M. Diepenbrock, M. Kenis, J. Zhang, X. Chen, N. Borowiec, B. Facon, H. Vogt, D. K. Price, H. Vogel, B. Prud'homme, A. Estoup, M. Gautier, A whole-genome scan for association with invasion success in the fruit fly *Drosophila suzukii* using contrasts of allele frequencies corrected for population structure. *Mol. Biol. Evol.* **37**, 2369–2385 (2020).

63. M. Gautier, Genome-wide scan for adaptive divergence and association with population-specific covariates. *Genetics* **201**, 1555–1579 (2015).

64. S. Hirohata, L. W. Wang, M. Miyagi, L. Yan, M. F. Seldin, D. R. Keene, J. W. Crabb, S. S. Apte, Punctin, a novel ADAMTS-like molecule, ADAMTSL-1, in extracellular matrix*. *J. Biol. Chem.* **277**, 12182–12189 (2002).

65. B. Pinan-Lucarré, H. Tu, M. Pierron, P. I. Cruceyra, H. Zhan, C. Stigloher, J. E. Richmond, J.-L. Bessereau, *C. elegans* punctin specifies cholinergic versus GABAergic identity of postsynaptic domains. *Nature* **511**, 466–470 (2014).

66. A. Yildiz, Mechanism and regulation of kinesin motors. *Nat. Rev. Mol. Cell Biol.* **26**, 86–103 (2025).

67. H. Voss, U. Wirkner, R. Jakobi, N. A. Hewitt, C. Schwager, J. Zimmermann, W. Ansorge, W. Pyerin, structure of the gene encoding human casein kinase II subunit beta. *J. Biol. Chem.* **266**, 13706–13711 (1991).

68. J. Aruga, N. Yokota, K. Mikoshiba, Human SLITRK family genes: Genomic organization and expression profiling in normal brain and brain tumor tissue. *Gene* **315**, 87–94 (2003).

69. N. Zhang, C. Zhao, X. Zhang, X. Cui, Y. Zhao, J. Yang, X. Gao, Growth arrest–specific 2 protein family: Structure and function. *Cell Prolif.* **54**, e12934 (2021).

70. C. Yang, F. Wu, X. Lu, M. Jiang, W. Liu, L. Yu, J. Tian, H. Wen, Growth arrest specific gene 2 in tilapia (*Oreochromis niloticus*): Molecular characterization and functional analysis under low-temperature stress. *BMC Mol. Biol.* **18**, 18 (2017).

71. P. Simó-Mirabet, F. Naya-Català, J. A. Calduch-Giner, J. Pérez-Sánchez, The expansion of sirtuin gene family in gilthead sea bream (*Sparus aurata*) – Phylogenetic, syntenic, and functional insights across the vertebrate/fish lineage. *Int. J. Mol. Sci.* **25**, 6273 (2024).

72. A. J. McVie-Wylie, D. R. Lamson, Y. T. Chen, Molecular cloning of a novel member of the GLUT family of transporters, SLC2A10 (GLUT10), localized on chromosome 20q13.1: A candidate gene for NIDDM susceptibility. *Genomics* **72**, 113–117 (2001).

73. F. Beby, T. Lamonerie, The homeobox gene Otx2 in development and disease. *Exp. Eye Res.* **111**, 9–16 (2013).

74. Q. Zhang, M. Geng, K. Li, H. Gao, X. Jiao, K. Ai, X. Wei, J. Yang, TGF-β1 suppresses the T-cell response in teleost fish by initiating Smad3- and Foxp3-mediated transcriptional networks. *J. Biol. Chem.* **299**, 102843 (2023).

75. S. T. Rodríguez-Ramilo, M. Baranski, H. Moghadam, H. Grove, S. Lien, M. E. Goddard, T. H. E. Meuwissen, A. K. Sonesson, Strong selection pressures maintain divergence on genomic islands in Atlantic cod (*Gadus morhua* L.) populations. *Genet. Sel. Evol.* **51**, 61 (2019).

76. T. Johansen, F. Besnier, M. Quintela, P. E. Jorde, K. A. Glover, J. Westgaard, G. Dahle, S. Lien, M. P. Kent, Genomic analysis reveals neutral and adaptive patterns that challenge the current management regime for East Atlantic cod *Gadus morhua* L. *Evol. Appl.* **13**, 2673–2688 (2020).

77. P. Gullestad, S. Sundby, O. S. Kjesbu, Management of transboundary and straddling fish stocks in the Northeast Atlantic in view of climate-induced shifts in spatial distribution. *Fish Fish.* **21**, 1008–1026 (2020).

78. J. T. Nordeide, U. Båmstedt, Coastal cod and north-east Arctic cod – Do they mingle at the spawning grounds in Lofoten? *Sarsia* **83**, 373–379 (2012).

79. T. Zhang, B. Dayanandan, I. Rouiller, E. J. Lawrence, C. A. Mandato, Growth-arrest-specific protein 2 inhibits cell division in *Xenopus* embryos. *PLOS ONE* **6**, e24698 (2011).

80. J. Goodall, M. E. Pettersson, U. Bergström, A. Cocco, B. Delling, Y. Heimbrand, O. M. Karlsson, J. Larsson, H. Waldetoft, A. Wallberg, L. Wennerström, L. Andersson, Evolution of fast-growing piscivorous herring in the young Baltic Sea. *Nat. Commun.* **15**, 10707 (2024).

81. A. S. Burrell, T. R. Disotell, C. M. Bergey, The use of museum specimens with high-throughput DNA sequencers. *J. Hum. Evol.* **79**, 35–44 (2015).

82. N. H. Barton, P. D. Keightley, Understanding quantitative genetic variation. *Nat. Rev. Genet.* **3**, 11–21 (2002).

83. M. Akopyan, A. Tigano, A. Jacobs, A. P. Wilder, H. Baumann, N. O. Therkildsen, Comparative linkage mapping uncovers recombination suppression across massive chromosomal inversions associated with local adaptation in Atlantic silversides. *Mol. Ecol.* **31**, 3323–3341 (2022).

84. D. Righton, K. Andersen, F. Neat, V. Thorsteinsson, P. Steingrund, H. Svedäng, K. Michalsen, H. Hinrichsen, V. Bendall, S. Neuenfeldt, P. Wright, P. Jonsson, G. Huse, J. van der Kooij, H. Mosegaard, K. Hüssy, J. Metcalfe, Thermal niche of Atlantic cod *Gadus morhua*: Limits, tolerance and optima. *Mar. Ecol. Prog. Ser.* **420**, 1–13 (2010).

85. O. S. Kjesbu, D. Righton, M. Krger-Johnsen, A. Thorsen, K. Michalsen, M. Fonn, P. R. Witthames, Thermal dynamics of ovarian maturation in Atlantic cod (*Gadus morhua*). *Can. J. Fish. Aquat. Sci.* **67**, 605–625 (2010).

86. A. J. Geffen, C. J. Fox, R. D. M. Nash, Temperature-dependent development rates of cod *Gadus morhua* eggs. *J. Fish Biol.* **69**, 1060–1080 (2006).

87. G. Ottersen, H. Loeng, Covariability in early growth and year-class strength of Barents Sea cod, haddock, and herring: The environmental link. *ICES J. Mar. Sci.* **57**, 339–348 (2000).

88. G. Beaugrand, K. M. Brander, J. A. Lindley, S. Souissi, P. C. Reid, Plankton effect on cod recruitment in the North Sea. *Nature* **426**, 661–664 (2003).

89. B. Ellertsen, P. Fossum, P. Solemdal, S. Sundby, Relation between temperature and survival of eggs and first-feeding larvae of northeast Arctic cod (*Gadus morhua* L.). *Rapp. p.-v. réun.* **191**, 209–219 (1989).

90. A. Hylen, O. Nakken, K. Nedreaas, "Northeast Arctic cod: Fisheries, life history, stock fluctuations and management" in *Norwegian Spring-Spawning Herring & Northeast Arctic Cod. 100 Years of Research and Management* (Tapir Academic Press, 2008), pp. 83–118.

91. D. Standal, B. Hersoug, Shaping technology, building society; the industrialization of the Norwegian cod fisheries. *Mar. Polic.* **51**, 66–74 (2015).

92. B. Charlesworth, T. Flatt, On the fixation or nonfixation of inversions under epistatic selection. *Mol. Ecol.* **30**, 3896–3897 (2021).

93. R. A. J. Case, W. F. Hutchinson, L. Hauser, V. Buehler, C. Clemmesen, G. Dahle, O. S. Kjesbu, E. Moksness, H. Otterå, H. Paulsen, T. Svåsand, A. Thorsen, G. R. Carvalho,

Association between growth and Pan I* genotype within Atlantic cod full-sibling families. *Trans. Am. Fish. Soc.* **135**, 241–250 (2006).

94. C. Pampoulie, D. E. Ruzzante, V. Chosson, T. D. Jrundsdttir, L. Taylor, V. Thorsteinsson, A. K. Danelsdttir, G. Marteinsdttir, The genetic structure of Atlantic cod (*Gadus morhua*) around Iceland: Insight from microsatellites, the Pan I locus, and tagging experiments. *Can. J. Fish. Aquat. Sci.* **63**, 2660–2674 (2006).

95. J. Hurrell, Y. Kushnir, G. Ottersen, M. Visbeck, *The North Atlantic Oscillation: Climate Significance and Environmental Impact* (American Geophysical Union, Washington, DC, 2003).

96. C. A. K. Endo, L. C. Stige, M. D. Skogen, L. Ciannelli, F. V. Vikebø, Two decades of match-mismatch in Northeast Arctic cod – Feeding conditions and survival. *Front. Mar. Sci.* **9**, doi.org/10.3389/fmars.2022.767290 (2022).

97. N. V. Mukhina, C. T. Marshall, N. A. Yaragina, Tracking the signal in year-class strength of Northeast Arctic cod through multiple survey estimates of egg, larval and juvenile abundance. *J. Sea Res.* **50**, 57–75 (2003).

98. A. Ferreira, L. Stige, A. Neuheimer, B. Bogstad, N. Yaragina, I. Prokopchuk, J. Durant, Match-mismatch dynamics in the Norwegian-Barents Sea system. *Mar. Ecol. Prog. Ser.* **650**, 81–94 (2020).

99. J. A. Castillo, S. N. Agathos, A genome-wide scan for genes under balancing selection in the plant pathogen *Ralstonia solanacearum*. *BMC Evol. Biol.* **19**, 123 (2019).

100. N. Eckshtain-Levi, A. J. Weisberg, B. A. Vinatzer, The population genetic test Tajima's D identifies genes encoding pathogen-associated molecular patterns and other virulence-related genes in *Ralstonia solanacearum*. *Mol. Plant Pathol.* **19**, 2187–2192 (2018).

101. H. Mjanger, K. Nedreaas, H. Senneset, P. Ågotnes, "*Procedure for Age Estimation of Cod, Haddock and Saithe*." (Institute of Marine Research, Bergen, Norway, 2000).

102. M.-H. S. Sinding, M. T. P. Gilbert, B. Grønnow, H. C. Gulløv, P. A. Toft, A. D. Foote, Minimally destructive DNA extraction from archaeological artefacts made from whale baleen. *J. Archaeol. Sci.* **39**, 3750–3753 (2012).

103. P. Danecek, J. K. Bonfield, J. Liddle, J. Marshall, V. Ohan, M. O. Pollard, A. Whitwham, T. Keane, S. A. McCarthy, R. M. Davies, H. Li, Twelve years of SAMtools and BCFtools. *Gigascience* **10**, giab008 (2021).

104. P. Danecek, A. Auton, G. Abecasis, C. A. Albers, E. Banks, M. A. DePristo, R. E. Handsaker, G. Lunter, G. T. Marth, S. T. Sherry, G. McVean, R. Durbin, 1000 Genomes Project Analysis Group, 1000 Genomes Project Analysis Group, The variant call format and VCFtools. *Bioinformatics* **27**, 2156–2158 (2011).

105. T. Derrien, J. Estellé, S. M. Sola, D. G. Knowles, E. Raineri, R. Guigó, P. Ribeca, Fast computation and applications of genome mappability. *PLOS ONE* **7**, e30377 (2012).

106. D. Sims, I. Sudbery, N. E. Ilott, A. Heger, C. P. Ponting, Sequencing depth and coverage: Key considerations in genomic analyses. *Nat. Rev. Genet.* **15**, 121–132 (2014).

107. C. Mérot, Making the most of population genomic data to understand the importance of chromosomal inversions for adaptation and speciation. *Mol. Ecol.* **29**, 2513–2516 (2020).

108. J. R. Whiting, *JimWhiting91/Genotype_plot: Genotype Plot* (Zenodo, 2022; https://doi.org/10.5281/zenodo.5913504).

109. J. T. Robinson, H. Thorvaldsdóttir, W. Winckler, M. Guttman, E. S. Lander, G. Getz, J. P. Mesirov, Integrative genomics viewer. *Nat. Biotechnol.* **29**, 24–26 (2011).

110. M. Gautier, A. Klassmann, R., Vitalis, rehh 2.0: A reimplementation of the R package rehh to detect positive selection from haplotype structure. *Mol. Ecol. Resour.* **17**, 78–90 (2017).

111. O. Delaneau, J. Marchini, J.-F. Zagury, A linear complexity phasing method for thousands of genomes. *Nat. Methods* **9**, 179–181 (2012).

112. A. Dinno, *Dunn.Test: Dunn's Test of Multiple Comparisons Using Rank Sums* (2017; https://CRAN.R-project.org/package=dunn.test).

113. E. Garrison, Z. N. Kronenberg, E. T. Dawson, B. S. Pedersen, P. Prins, A spectrum of free software tools for processing the VCF variant call format: vcflib, bio-vcf, cyvcf2, hts-nim and slivar. *PLoS Comput. Biol.* **18**, e1009123 (2022).

114. J. D. Storey, A. J. Bass, Q-value estimation for false discovery rate control. R package version 2.30.0, 2022 (2022). http://github.com/jdstorey/qvalue.

115. O. K. Tørresen, B. Star, S. Jentoft, W. B. Reinar, H. Grove, J. R. Miller, B. P. Walenz, J. Knight, J. M. Ekholm, P. Peluso, R. B. Edvardsen, A. Tooming-Klunderud, M. Skage, S. Lien, K. S. Jakobsen, A. J. Nederbragt, An improved genome assembly uncovers prolific tandem repeats in Atlantic cod. *BMC Genomics* **18**, 95 (2017).

116. K. Stuart, *Outlier Analysis Workshop* (2024). https://github.com/katarinastuart/Ev1_SelectionMetaAnalysis?tab=readme-ov-file.

117. T. Günther, G. Coop, Robust identification of local adaptation from allele frequencies. *Genetics* **195**, 205–220 (2013).

118. M. Vihtakari, ggOceanMaps: Plot Data on Oceanographic Maps using ggplot2 R package version 1.3.7, 2022 (2022). https://mikkovihtakari.github.io/ggOceanMaps/.

119. FAO, Aquatic Species Distribution Map Viewer (2022). https://fao.org/fishery/geoserver/factsheets/species.html.

120. IMR, GeoServer, Layer Preview (2022). https://kart.hi.no/data/web/wicket/bookmarkable/org.geoserver.web.demo.MapPreviewPage?2&filter=false.

121. G. Stefánsson, Analysis of groundfish survey abundance data: Combining the GLM and delta approaches. *ICES J. Mar. Sci.* **53**, 577–588 (1996).

122. D. Bates, M. Mächler, B. Bolker, S. Walker, Fitting linear mixed-effects models using lme4. *J. Stat. Softw.* **67**, (2015).

123. B. Efron, T. R. J, *An Introduction to the Bootstrap* (Boca Raton, Fla, 1993) *Monographs on Statistics and Applied Probability*.

124. A. Webb, J. Knoblauch, N. Sabankar, A. S. Kallur, J. Hey, A. Sethuraman, The Pop-Gen Pipeline Platform: A software platform for population genomic analyses. *Mol. Biol. Evol.* **38**, 3478–3485 (2021).

125. W. Iwasaki, T. Fukunaga, R. Isagozawa, K. Yamada, Y. Maeda, T. P. Satoh, T. Sado, K. Mabuchi, H. Takeshima, M. Miya, M. Nishida, MitoFish and MitoAnnotator: A mitochondrial genome database of fish with an accurate and automatic annotation pipeline. *Mol. Biol. Evol.* **30**, 2531–2540 (2013).

126. Y. Sato, M. Miya, T. Fukunaga, T. Sado, W. Iwasaki, MitoFish and MiFish Pipeline: A mitochondrial genome database of fish with an analysis pipeline for environmental DNA metabarcoding. *Mol. Biol. Evol.* **35**, 1553–1555 (2018).

127. C. Camacho, G. Coulouris, V. Avagyan, N. Ma, J. Papadopoulos, K. Bealer, T. L. Madden, BLAST+: Architecture and applications. *BMC Bioinformatics* **10**, 421–421 (2009).

128. K. Katoh, D. M. Standley, MAFFT Multiple Sequence Alignment Software Version 7: Improvements in performance and usability. *Mol. Biol. Evol.* **30**, 772–780 (2013).

129. B. Q. Minh, H. A. Schmidt, O. Chernomor, D. Schrempf, M. D. Woodhams, A. von Haeseler, R. Lanfear, IQ-TREE 2: New models and efficient methods for phylogenetic inference in the genomic era. *Mol. Biol. Evol.* **37**, 1530–1534 (2020).

130. S. Kalyaanamoorthy, B. Q. Minh, T. K. Wong, A. von Haeseler, L. S. Jermiin, ModelFinder: Fast model selection for accurate phylogenetic estimates. *Nat. Methods* **14**, 587–589 (2017).

131. M. Matschiner, Fitchi: Haplotype genealogy graphs based on the Fitch algorithm. *Bioinformatics* **32**, 1250–1252 (2016).

132. L. Martínez-García, G. Ferrari, T. Oosting, R. Ballantyne, I. van der Jagt, I. Ystgaard, J. Harland, R. Nicholson, S. Hamilton-Dyer, H. T. Baalsrud, M. S. O. Brieuc, L. M. Atmore, F. Burns, U. Schmölcke, K. S. Jakobsen, S. Jentoft, D. Orton, A. K. Hufthammer, J. H. Barrett,

B. Star, Historical demographic processes dominate genetic variation in ancient Atlantic cod mitogenomes. *Front. Ecol. Evol.* **9**, 671281 (2021).

133. T. B. Hallett, T. Coulson, J. G. Pilkington, T. H. Clutton-Brock, J. M. Pemberton, B. T. Grenfell, Why large-scale climate indices seem to predict ecological processes better than local weather. *Nature* **430**, 71–75 (2004).

134. N. C. Stenseth, G. Ottersen, J. W. Hurrell, A. Mysterud, M. Lima, K. S. Chan, N. G. Yoccoz, B. Adlandsvik, Studying climate effects on ecology through the use of climate indices: The North Atlantic Oscillation, El Niño Southern Oscillation and beyond. *Proc. Biol. Sci.* **270**, 2087–2096 (2003).

135. D. Ø. Hjermann, B. Bogstad, A. M. Eikeset, G. Ottersen, H. Gjosaeter, N. C. Stenseth, Food web dynamics affect Northeast Arctic cod recruitment. *Proc. Biol. Sci.* **274**, 661–669 (2007).

136. G. Ottersen, N. C. Stenseth, Atlantic climate governs oceanographic and ecological variability in the Barents Sea. *Limnol. Oceanogr.* **46**, 1774–1780 (2001).

137. L. C. Stige, G. Ottersen, K. Brander, K. S. Chan, N. C. Stenseth, Cod and climate: Effect of the North Atlantic Oscillation on recruitment in the North Atlantic. *Mar. Ecol. Prog. Ser.* **325**, 227–241 (2006).

138. B. Ådlandsvik, H. Loeng, A study of the climatic system in the Barents Sea. *Polar Res* **10**, 45–50 (1991).

139. R. R. Dickson, T. J. Osborn, J. W. Hurrell, J. Meincke, J. Blindheim, B. Adlandsvik, T. Vinje, G. Alekseev, W. Maslowski, The Arctic Ocean response to the North Atlantic Oscillation. *J. Clim.* **13**, 2671–2696 (2000).

140. G. E. Dingsør, L. Ciannelli, K. S. Chan, G. Ottersen, N. C. Stenseth, Density dependence and density independence during the early life stages of four marine fish stocks. *Ecology* **88**, 625–634 (2007).

141. M. Hidalgo, Y. Gusdal, G. E. Dingsor, D. Hjermann, G. Ottersen, L. C. Stige, A. Melsom, N. C. Stenseth, A combination of hydrodynamical and statistical modelling reveals non-stationary climate effects on fish larvae distributions. *Proc. Biol. Sci.* **279**, 275–283 (2012).

142. G. Ottersen, L. C. Stige, J. M. Durant, K. S. Chan, T. A. Rouyer, K. F. Drinkwater, N. C. Stenseth, Temporal shifts in recruitment dynamics of North Atlantic fish stocks: Effects of spawning stock and temperature. *Mar. Ecol. Prog. Ser.* **480**, 205–225 (2013).

143. D. Ø. Hjermann, N. C. Stenseth, G. Ottersen, The population dynamics of Northeast Arctic cod (*Gadus morhua*) through two decades: An analysis based on survey data. *Can. J. Fish. Aquat. Sci.* **61**, 1747–1755 (2004).

144. J. Fall, L. Ciannelli, G. Skaret, E. Johannesen, Seasonal dynamics of spatial distributions and overlap between Northeast Arctic cod (*Gadus morhua*) and capelin (*Mallotus villosus*) in the Barents Sea. *PLOS ONE* **13**, e0205921 (2018).

145. J. M. Durant, N. Dupont, K. Ono, Ø. Langangen, Interaction between three key species in the sea ice-reduced Arctic Barents Sea system. *Proc. R. Soc. B* **291**, 20241408 (2024).

146. M. A. Giorgetta, J. H. Jungclaus, C. H. Reick, S. Legutke, V. Brovkin, T. Crueger, M. Esch, K. Fieg, K. Glushak, V. Gayler, H. Haak, H.-D. Hollweg, T. Ilyina, S. Kinne, L. Kornblueh, D. Matei, T. Mauritsen, U. Mikolajewicz, W. A. Mueller, D. Notz, T. Raddatz, S. Rast, R. Redler, E. Roeckner, H. Schmidt, R. Schnur, J. Segschneider, K. Six, M. Stockhause, J. Wegner, H. Widmann, K.-H. Wieners, M. Claussen, J. Marotzke, B. Stevens, Climate change from 1850 to 2100 in MPI-ESM simulations for the Coupled Model Intercomparison Project 5. *J. Adv. Model. Earth Syst.* **5**, 572–597 (2013).

147. A. Hylen, Fluctuations in abundance of Northeast Arctic cod during the 20th century. *ICES Mar. Sci. Symp.* **215**, 543–550 (2002).

148. ICES, Report of the Arctic Fisheries Working Group (AFWG) 18–24 April 2018, Ispra, Italy. *ICES CM 2018/ACOM* **6**, 859 pp (2018).

149. J. M. Durant, D. Ø. Hjermann, Age-structure, harvesting and climate effects on population growth of Arcto-boreal fish stocks. *Mar. Ecol. Prog. Ser.* **577**, 177–188 (2017).

150. M. Meyer, M. Kircher, Illumina sequencing library preparation for highly multiplexed target capture and sequencing. *Cold Spring Harb. Protoc.* **2010**, pdb.prot5448 (2010).

151. M. Schubert, S. Lindgreen, L. Orlando, AdapterRemoval v2: Rapid adapter trimming, identification, and read merging. *BMC. Res. Notes* **9**, 88 (2016).

152. B. Star, A. J. Nederbragt, S. Jentoft, U. Grimholt, M. Malmstrøm, T. F. Gregers, T. B. Rounge, J. Paulsen, M. H. Solbakken, A. Sharma, O. F. Wetten, A. Lanzén, R. Winer, J. Knight, J.-H. Vogel, B. Aken, Ø. Andersen, K. Lagesen, A. Tooming-Klunderud, R. B. Edvardsen, K. G. Tina, M. Espelund, C. Nepal, C. Previti, B. O. Karlsen, T. Moum, M. Skage, P. R. Berg, T. Gjøen, H. Kuhl, J. Thorsen, K. Malde, R. Reinhardt, L. Du, S. D. Johansen, S. Searle, S. Lien, F. Nilsen, I. Jonassen, S. W. Omholt, N. C. Stenseth, K. S. Jakobsen, The genome sequence of Atlantic cod reveals a unique immune system. *Nature* **477**, 207–210 (2011).

153. H. Li, R. Durbin, Fast and accurate short read alignment with Burrows–Wheeler transform. *Bioinformatics* **25**, 1754–1760 (2009).

154. H. Li, Aligning sequence reads, clone sequences and assembly contigs with BWA-MEM. arXiv:1303.3997 (2013).

155. A. McKenna, M. Hanna, E. Banks, A. Sivachenko, K. Cibulskis, A. Kernytsky, K. Garimella, D. Altshuler, S. Gabriel, M. Daly, M. A. DePristo, The Genome Analysis Toolkit: A MapReduce framework for analyzing next-generation DNA sequencing data. *Genome Res.* **20**, 1297–1303 (2010).

156. H. Jónsson, A. Ginolhac, M. Schubert, P. L. F. Johnson, L. Orlando, mapDamage2.0: Fast approximate Bayesian estimates of ancient DNA damage parameters. *Bioinformatics* **29**, 1682–1684 (2013).

157. M. Schubert, L. Ermini, C. D. Sarkissian, H. Jónsson, A. Ginolhac, R. Schaefer, M. D. Martin, R. Fernández, M. Kircher, M. McCue, E. Willerslev, L. Orlando, Characterization of ancient and modern genomes by SNP detection and phylogenomic and metagenomic analysis using PALEOMIX. *Nat. Protoc.* **9**, 1056–1082 (2014).

158. H. Li, B. Handsaker, A. Wysoker, T. Fennell, J. Ruan, N. Homer, G. Marth, G. Abecasis, R. Durbin, 1000 Genome Project Data Processing Subgroup, 1000 Genome Project Data Processing Subgroup, The Sequence Alignment/Map format and SAMtools. *Bioinformatics* **25**, 2078–2079 (2009).

159. A. Kijewska, A. Burzynski, R. Wenne, Variation in the copy number of tandem repeats of mitochondrial DNA in the North-East Atlantic cod populations. *Mar. Biol. Res.* **5**, 186–192 (2009).

160. S. Purcell, B. Neale, K. Todd-Brown, L. Thomas, M. A. R. Ferreira, D. Bender, J. Maller, P. Sklar, P. I. W. de Bakker, M. J. Daly, P. C. Sham, PLINK: A tool set for whole-genome association and population-based linkage analyses. *Am. J. Hum. Genet.* **81**, 559–575 (2007).

161. T. O. Elgvin, C. N. Trier, O. K. Tørresen, I. J. Hagen, S. Lien, A. J. Nederbragt, M. Ravinet, H. Jensen, G.-P. Sætre, The genomic mosaicism of hybrid speciation. *Sci. Adv.* **3**, e1602996 (2017).

162. P. Nilsson, M. Ravinet, Y. Cui, P. R. Berg, Y. Zhang, R. Guo, T. Luo, Y. Song, E. Trucchi, S. N. K. Hoff, R. Lv, B. V. Schmid, W. R. Easterday, K. S. Jakobsen, N. C. Stenseth, R. Yang, S. Jentoft, Polygenic plague resistance in the great gerbil uncovered by population sequencing. *PNAS Nexus* **1**, pgac211 (2022).

163. B. Star, S. Boessenkool, A. T. Gondek, E. A. Nikulina, A. K. Hufthammer, C. Pampoulie, H. Knutsen, C. André, H. M. Nistelberger, J. Dierking, C. Petereit, D. Heinrich, K. S. Jakobsen, N. C. Stenseth, S. Jentoft, J. H. Barrett, Ancient DNA reveals the Arctic origin of Viking Age cod from Haithabu, Germany. *Proc. Natl. Acad. Sci. U.S.A.* **114**, 9152–9157 (2017).

164. P. R. Berg, B. Star, C. Pampoulie, I. R. Bradbury, P. Bentzen, J. A. Hutchings, S. Jentoft, K. S. Jakobsen, Trans-oceanic genomic divergence of Atlantic cod ecotypes is associated with large inversions. *Heredity* **119**, 418–428 (2017).
